# Immunopathogenic CSF TCR repertoire signatures in virus-associated neurologic disease

**DOI:** 10.1172/jci.insight.144869

**Published:** 2021-02-22

**Authors:** Satoshi Nozuma, Yoshimi Enose-Akahata, Kory R. Johnson, Maria Chiara Monaco, Nyater Ngouth, Abdel Elkahloun, Joan Ohayon, Jun Zhu, Steven Jacobson

**Affiliations:** 1 Viral Immunology Section, Neuroimmunology Branch and; 2 Bioinformatics Section, National Institute of Neurological Disorders and Stroke, NIH, Bethesda, Maryland, USA.; 3 Comparative Genomics and Cancer Genetics Branch, National Human Genome Research Institute, NIH, Bethesda, Maryland, USA.; 4 Neuroimmunology Clinic, National Institute of Neurological Disorders and Stroke, NIH, Bethesda, Maryland, USA.; 5 Mokobio Biotechnology R&D Center, Rockville, Maryland, USA.

**Keywords:** Immunology, Infectious disease, Neurological disorders, T cell receptor

## Abstract

In this study, we examined and characterized disease-specific TCR signatures in cerebrospinal fluid (CSF) of patients with HTLV-1–associated myelopathy/tropical spastic paraparesis (HAM/TSP). TCR β libraries using unique molecular identifier–based methodologies were sequenced in paired peripheral blood mononuclear cells (PBMCs) and CSF cells from HAM/TSP patients and normal healthy donors (NDs). The sequence analysis demonstrated that TCR β repertoires in CSF of HAM/TSP patients were highly expanded and contained both TCR clonotypes shared with PBMCs and uniquely enriched within the CSF. In addition, we analyzed TCR β repertoires of highly expanded and potentially immunopathologic HTLV-1 Tax11-19–specific CD8^+^ T cells from PBMCs of HLA-A*0201^+^ HAM/TSP and identified a conserved motif (PGLAG) in the CDR3 region. Importantly, TCR β clonotypes of expanded clones in HTLV-1 Tax11-19–specific CD8^+^ T cells were also expanded and enriched in the CSF of the same patient. These results suggest that exploring TCR repertoires of CSF and antigen-specific T cells may provide a TCR repertoire signature in virus-associated neurologic disorders.

## Introduction

Human T-lymphotropic type 1 (HTLV-1) infects 10 million–20 million people worldwide and is the etiologic agent of adult T cell leukemia/lymphoma (1) and an inflammatory neurologic disease, HTLV-1–associated myelopathy/tropical spastic paraparesis (HAM/TSP) (2). HAM/TSP is a neuroinflammatory disease of the spinal cord with progressive spastic paraparesis and bladder dysfunction. HTLV-1 predominantly infects CD4^+^ T cells and causes the activation and proliferation of infected cells, which express viral proteins that may lead to the activation and expansion of HTLV-1–specific CD8^+^ cytotoxic T lymphocytes (CTLs) (3, 4). The HTLV-1 regulatory protein Tax promotes the proliferation of HTLV-1–infected lymphocytes and is an immunodominant antigen recognized by HTLV-1–specific CTLs (5). The frequency of these Tax-specific CTLs is extraordinarily high in peripheral blood and even higher in cerebrospinal fluid (CSF) (6), in which virus-specific CTLs demonstrate degranulation activity and produce proinflammatory cytokines (3, 4). It has been suggested that HTLV-1–infected CD4^+^ T cells and HTLV-1–specific CD8^+^ cytotoxic T cells enter the CNS and release various proinflammatory cytokines, resulting in the neural damage and degeneration in HAM/TSP patients (7).

T cell–mediated antigen recognition depends on the interaction of the T cell receptor (TCR) with antigen–major histocompatibility complex (antigen-MHC) molecules (8). Although the actual diversity of human TCR repertoires remains unknown, recent estimates of the potential number of TCR repertoires have been reported to be highly diverse in the range from 1 × 10^13^ to 1 × 10^20^ in humans (9–11). TCR diversity is generated through random rearrangement of variable regions (V), diversity (D) of the β chain, and joining (J) segments of TCR genes (VDJ rearrangements) and is a central component of the adaptive immune system (10). T cells become activated and clonally expanded after they encounter their cognate antigen (12). Importantly, an individual’s TCR repertoire can be altered in the context of infections, malignancies, or immunological disorders. Analysis of the TCR repertoire can, thus, provide a better understanding of immune-mediated responses in neuroinflammatory diseases. Recent technological advances based on high-throughput sequencing (HTS), bioinformatics software, and unbiased gene amplification allow for the analysis of millions of TCR sequences (13). We have recently reported on an unbiased molecular technique that combines the use of unique molecular identifier (UMI), which is barcoded cDNA TCR transcript, with a previously published UMI-based TCR β data sequence analysis pipeline (14, 15). This robust UMI-based approach has been used successfully to assess the clonal expansion and diversity of the TCR repertoire in peripheral blood of HAM/TSP, multiple sclerosis (MS), and normal healthy donors (NDs), which demonstrated higher clonal T cell expansions in HAM/TSP compared with MS and NDs (14). Disease-associated TCR signatures could, thus, be useful biomarkers for immune-mediated disease diagnosis, prognosis, and response to treatment.

While we have demonstrated higher TCR clonal expansions in the peripheral blood of HAM/TSP compared with controls, no disease-specific TCR signatures have been identified (14). This could be due, in part, to observations that, in patients with neuroinflammatory neurologic disease, immune responses in the periphery may not reflect events in the CNS (16). In the CNS, robust immune responses to various viral infections and autoantigens have been demonstrated to be unique from the immune response observed in peripheral tissues. For example, comparison of TCR repertoires between blood and CSF in patients with MS showed an overlap of expanded clones, but with an intrathecal enrichment of Epstein-Barr virus–reactive (EBV-reactive) CD8^+^ T cells (17, 18), suggesting a role for EBV in the pathogenesis of MS. While the CSF is not the CNS, it is in close proximity to the brain, accessible by lumbar puncture and considered to reflect inflammation within the CNS better than in peripheral blood (19). Therefore, comparing TCR repertoires between the periphery and the CSF might be useful for exploring local TCR signatures in viral-mediated neurologic disease such as HAM/TSP. In addition, previous reports on the TCR repertoire analysis using PCR-based techniques showed higher clonal expansions, especially in CD8^+^ T cell subsets of HAM/TSP patients by complementarity determining region 3 (CDR3) spectratyping (20, 21) and some conserved motifs in the CDR3 region of Tax-specific CTL clones generated from HAM/TSP patients with HLA-A*0201 by cloning and sequencing (22, 23). Since chronically activated HTLV-1–specific CTLs are thought to have key roles in the pathogenesis of HAM/TSP (7), an unbiased, comprehensive analysis based on HTS to investigate TCR repertoire signatures of HTLV-1–specific CTLs would be useful to determine if such sequences can be demonstrated in lymphocytes present in peripheral blood and CSF of HAM/TSP patients.

In this study, we examined TCR β repertoires in paired peripheral blood mononuclear cells (PBMCs) and lymphocytes present in CSF from patients with HAM/TSP compared with NDs. Highly clonally expanded TCR clones were detected in CSF of HAM/TSP patients compared with the CSF of NDs. In addition, we analyzed TCR β repertoires of HTLV-1 Tax11-19–specific CD8^+^ T cells from PBMCs of HLA-A*0201^+^ HAM/TSP patients and identified that the expanded TCR clones in HTLV-1 Tax11-19–specific CD8^+^ T cells were also expanded and enriched in the CSF of HAM/TSP.

## Results

### T cell clones are highly expanded both in blood and CSF of HAM/TSP patients compared with NDs.

A total of 20 subjects consisting of 15 HAM/TSP patients and 5 NDs were examined for sequencing of TCR β chain repertoires in the study. The demographic characteristics of the study population are summarized in Table 1. To study whether the TCR repertoire skewing is present in peripheral blood or CSF of HAM/TSP patients, we compared the TCR repertoire of PBMCs from 9 HAM/TSP patients and 5 NDs in which paired CSF were also obtained. In the 14 subjects, mean age of NDs and HAM/TSP patients was 50.8 and 55.6 years, respectively (no statistically significant differences). CSF cell concentration was increased in the CSF of HAM/TSP patients compared with NDs, consistent with the known CSF pleocytosis in HAM/TSP (24) (Table 1). In HAM/TSP patients, the median HTLV-1 proviral load (PVL) was 10.20% (range, 4.90%–47.17%) in PBMCs and, as reported (25), was significantly higher in CSF (36.27% [range, 19.58%–53.33%]; *P* = 0.0039) (Table 1).

The TCR β sequence data, including the numbers of total HTS sequences, total UMIs, total unique TCR β clonotypes, and Shannon diversity from HAM/TSP patients and NDs are summarized in Table 2. Total numbers of sequence reads generated from HTS were not statistically different between HAM/TSP patients and NDs in either PBMCs or CSF (Supplemental Figure 1A; supplemental material available online with this article; https://doi.org/10.1172/jci.insight.144869DS1). On average, 631,318 and 360,179 reads were sequenced from the PBMCs and the CSF, respectively (Supplemental Figure 1A and Table 2). Comparison of the number of total unique TCR β clonotypes in PBMCs showed no significant differences between HAM/TSP patients and NDs, while in CSF, unique TCR β clonotypes were significantly higher in HAM/TSP patients compared with NDs (Supplemental Figure 1B and Table 2). TCR clonal size was classified into 3 groups based on the number of UMIs: (a) singletons (1 UMI); (b) clones with 2 ≤ UMIs < 8, and (c) expanded clones with ≥ 8 UMIs as previously described (14) (See Methods section for classification criteria).

**Figure 1:**
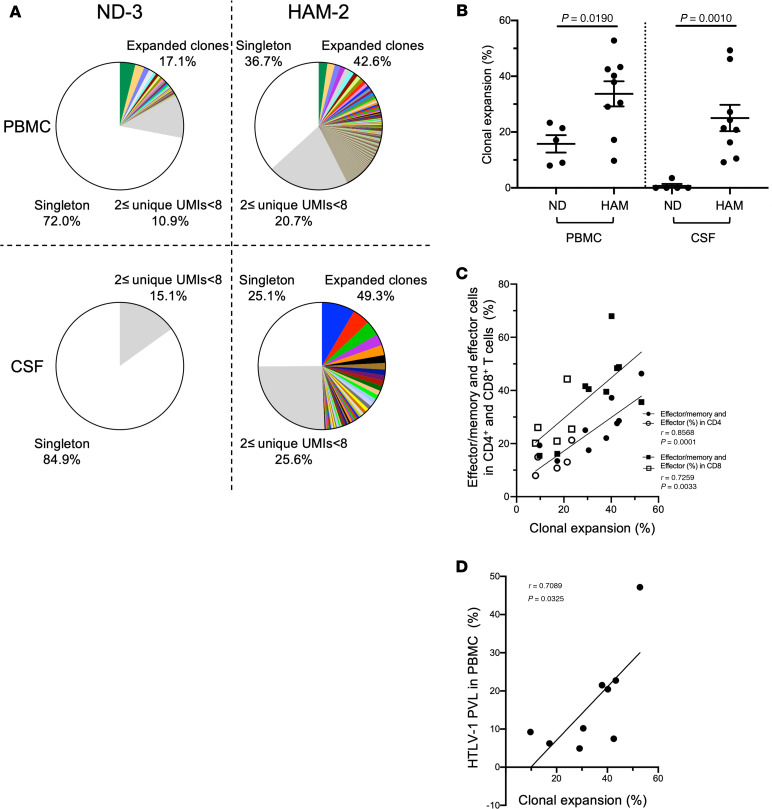
TCR clonal expansion of TCR β repertoire in paired PBMCs and CSF of HAM/TSP patients and NDs. (**A**) Representative analysis of T cell clonal expansion in PBMCs and CSF of a ND (ND-3) and a HAM/TSP patient (HAM-2). TCR β clonal expansion was analyzed by using the frequencies of clones ≥ 8 UMIs (colored wedges), clones with 2 ≤ UMIs < 8 (gray), and singletons (white). In the group of expanded clones, each wedge represents a unique clonotype with a defined CDR3 sequence, and the same clone shared by PBMCs and CSF in each individual is visualized in the same color. (**B**) Comparison of TCR clonal expansion by frequency of clones ≥ 8 UMIs between NDs (*n* = 5) and HAM/TSP patients (*n* = 9) in PBMCs and CSF using Mann-Whitney *U* test. Data represent mean ± SEM. (**C**) Correlation of TCR clonal expansion in PBMCs with frequencies of effector/memory and effector cells in PBMC CD4^+^ T cells (circles) and CD8^+^ T cells (squares) of NDs (opened shapes; *n* = 5) and HAM/TSP patients (closed shapes; *n* = 9) using Spearman’s rank correlation test. (**D**) Correlation of TCR clonal expansion in PBMCs with HTLV-1 PVL in PBMCs of HAM/TSP patients (*n* = 9) using Spearman’s rank correlation test.

The frequencies of expanded TCR clones from a representative HAM/TSP patient (HAM-2) and a ND (ND-3) are shown in Figure 1A. In this pie chart based on our definition of TCR clonal size, expanded clones with ≥ 8 UMIs are shown as colored wedges, while clones with 2 ≤ UMIs < 8 and singletons are visualized in the gray area and in the white area, respectively. Each colored wedge represents a unique clonotype with a defined CDR3 sequence. The same clone shared by PBMCs and CSF in each individual is visualized by the same color. Individual variations of clonal expansion were observed in PBMCs for both the ND and the HAM/TSP patient. By contrast in CSF, individual variations of clonal expansion were detected in the HAM/TSP patient but not in the ND (Figure 1A). Group analysis demonstrated that all HAM/TSP patients showed individual variations of clonal expansion in the CSF, while ND did not, except for the detection of only 1 expanded clone in 1 ND (ND-2; Supplemental Figure 2). These results are quantitated in Figure 1B, where each dot represents the frequency of expanded clones in PBMCs and CSF of HAM/TSP patients and NDs. HAM/TSP patients showed a higher degree of TCR clonal expansion in both PBMCs (*P* = 0.0190) and CSF (*P* = 0.0010), compared with NDs (Figure 1B). When the TCR clone diversity was estimated using the Shannon diversity index, a significantly lower diversity of TCR clones was observed in the peripheral blood of HAM/TSP patients compared with NDs (*P* = 0.029; Supplemental Figure 1C). In CSF, there was no significant difference in TCR diversity between HAM/TSP patients and NDs (Supplemental Figure 1C).

Since HAM/TSP patients have been reported to demonstrate high frequencies of effector/memory and effector CD8^+^ T cells (26), which suggests that they are driven by chronic antigen (viral) stimulation, we asked if this differentiation of T cells may also be associated with TCR repertoire diversity, clonal expansion, and HTLV-1 PVL. As shown in Figure 1C, the frequency of clonal expansion in PBMCs significantly correlated with T cell phenotype of effector/memory and effector cells both in CD4^+^ and CD8^+^ T cells. In addition, the frequency of TCR clonal expansion in PBMCs also showed significant correlations with HTLV-1 PVL in PBMCs (Figure 1D), but it did not show any correlation with disease duration or severity (data not shown). Collectively, these results demonstrate higher clonally expanded T cell clones in both PBMCs and CSF of HAM/TSP patients compared with NDs, with the degree of TCR clonal expansion particularly in the peripheral blood correlating with differentiated T cell phenotypes and HTLV-1 PVL.

### Overlap of TCR β repertoires of expanded clones between blood and CSF of HAM/TSP patients.

**Figure 2:**
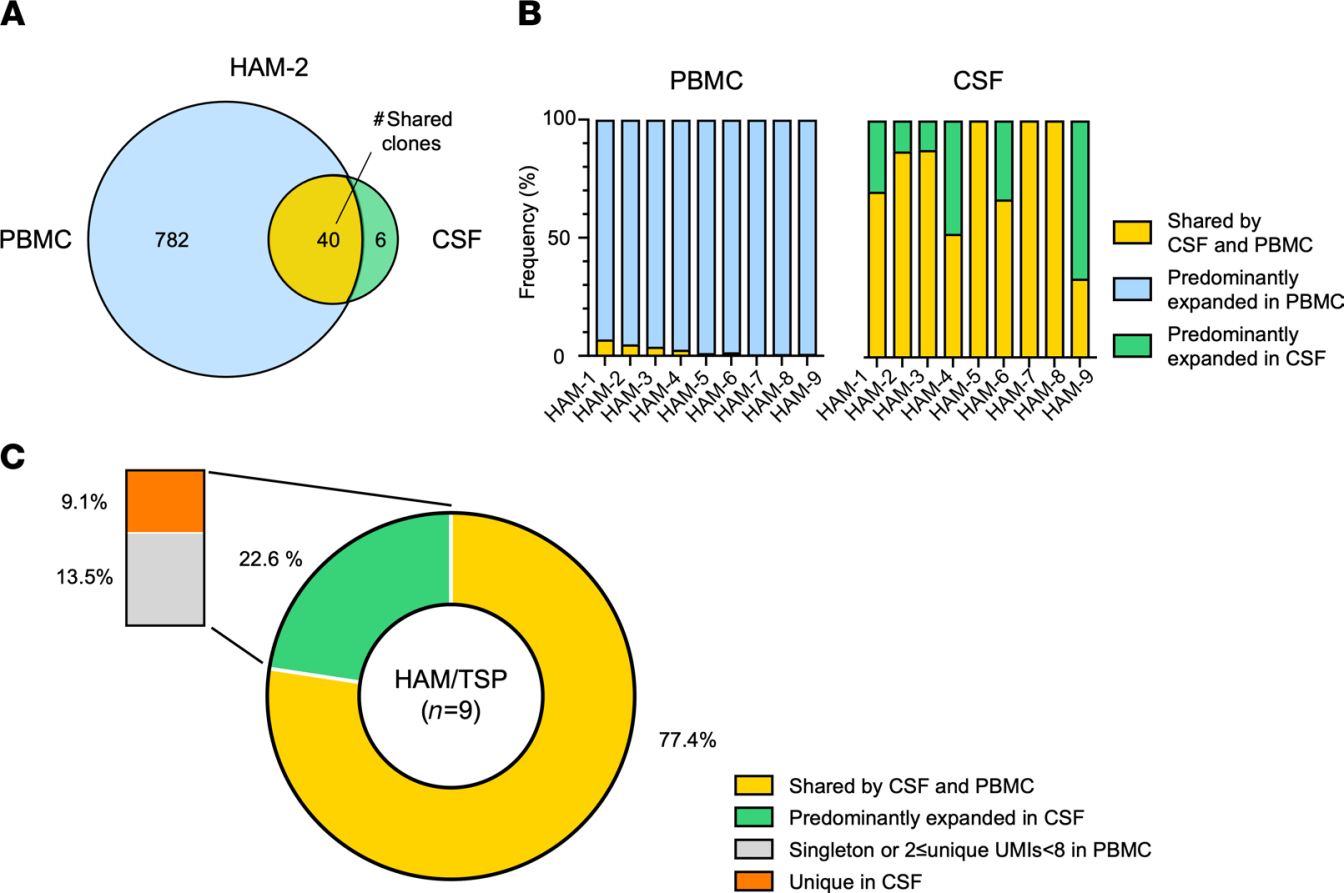
Overlap of TCR β repertoire of expanded clones between paired blood and CSF in patients with HAM/TSP. (**A**) Relationship of expanded TCR β clonotypes detected in PBMCs and CSF in a HAM/TSP patient (HAM-2) using a Venn diagram. The corresponding numbers of unique TCR β clonotype (≥8 UMIs) are given within the diagram. (**B**) Proportion of shared expanded clonotypes and predominantly expanded clonotypes in PBMCs and CSF in each patient with HAM/TSP (*n* = 9). (**C**) An average of the frequencies of shared clonotypes by CSF and PBMCs (yellow area) and predominantly expanded clonotypes in CSF (green area) in the whole TCR β expanded clonotypes of the CSF of HAM/TSP patients (*n* = 9). In predominantly expanded clonotypes in CSF, there are 2 subgroups: clonotypes detected at singleton or 2 ≤ UMIs < 8 in PBMCs (gray area) and unique clonotype in CSF (undetectable in PBMCs; orange area).

To evaluate whether there are any expanded TCR β clonotypes shared between PBMCs and CSF within each HAM/TSP patient, we compared the CDR3 sequences in the expanded clones (defined as unique clones with ≥ 8 UMIs) between PBMCs and CSF of HAM/TSP patients in order to determine if any of the CDR3 sequences were identical between PBMCs and CSF. A representative analysis of the overlap of the expanded TCR β clonotypes between paired PBMCs and CSF of a HAM/TSP patient (HAM-2) is shown in Figure 2A. Of the 822 expanded clonotypes in the PBMCs of HAM-2, only 40 clonotypes (4.9%) were shared with those clonotypes expanded in the CSF (Figure 2A; yellow area). Of the 46 TCR β expanded clonotypes in the CSF of HAM-2, 87.0% (40 of 46) were also found in the expanded clonotypes in the PBMCs (Figure 2A). Six expanded clonotypes were found to be unique in the CSF (Figure 2A; green area) — i.e., no sequences were found in the 822 expanded clonotypes in the PBMCs. Figure 2B shows the frequencies of the shared and unique TCR β expanded clonotypes detected in PBMCs (Figure 2B, left) and CSF (Figure 2B, right) in 9 HAM/TSP patients. In PBMCs of HAM/TSP patients, the expanded clonotypes shared with the CSF were detected at low frequencies (0.31%–6.99%; Figure 2B, left, bars in yellow), while almost all the expanded clonotypes were predominantly detected in the PBMCs (Figure 2B, left, bars in light blue). In the CSF, the expanded clonotypes shared with the PBMCs were detected at higher frequencies (33.33%–100.00%; Figure 2B, right, bars in yellow). Interestingly, of the 9 HAM/TSP patients, TCR β clonotypes predominantly expanded in the CSF were found in 6 patients (HAM-1, -2, -3, -4, -6, and -9; Figure 2B, right, green bars). Overall, 77.4% of expanded clonotypes in the CSF were shared by the expanded clonotypes (≥ 8 UMIs) in PBMCs (Figure 2C, yellow area), and 22.6% were predominantly expanded in CSF of HAM/TSP patients (Figure 2C, green area). A subset of these CSF expanded clonotypes (13.5%) may also have been expanded in PBMCs but did not reach our formal definition of expansion in PBMCs, since they had singleton or 2 ≤ UMIs < 8 (Figure 2C, gray area). Importantly, of the TCR β clonotypes predominantly expanded in the CSF, 9.1% were uniquely expanded in this compartment (Figure 2C, orange area). These results demonstrate that only a small subset of TCR β clonotypes in PBMCs was shared in the CSF, suggesting that most expanded CSF clonotypes were derived from expanded clonotypes in PBMCs in which a small but distinct fraction of these TCR-expanded clonotypes were intrathecally enriched.

### CDR3 relatedness of expanded T cell clonotypes in CSF of HAM/TSP patients.

**Figure 3:**
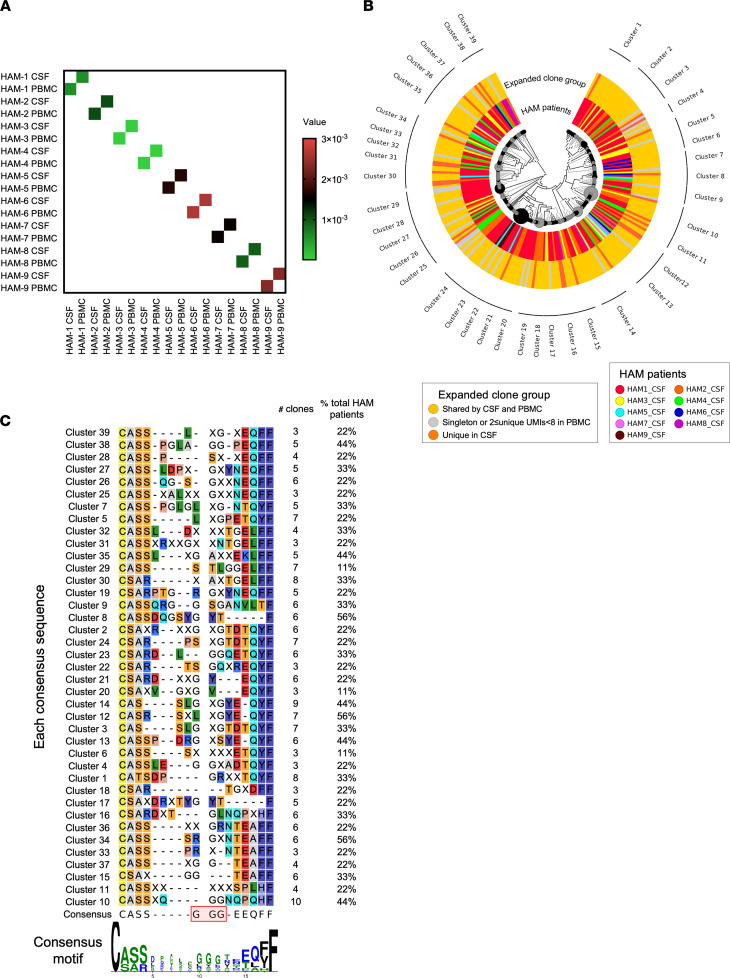
CDR3 sequence relatedness of TCR β clonotypes expanded in CSF of HAM/TSP patients. (**A**) A heatmap analysis represents the TCR β clonal repertoire in paired PBMCs and CSF of HAM/TSP patients (*n* = 9) by comparing the exact CDR3 amino acid sequences. (**B**) Phylogenetic tree analysis representing the similarities on the CDR3 amino acid sequences of TCR β repertoire in CSF of HAM/TSP patients (*n* = 9). Branch lengths represent the distance between repertoires, and node size is shown according to read counts. Inner layer represents 9 HAM/TSP patients with different colors. Outer layer represents 3 expanded clone groups with different colors; (a) expanded clones with ≥ 8 UMIs shared by CSF and PBMCs (in yellow), (b) expanded clones in CSF but detected at singleton or 2 ≤ UMIs < 8 in PBMCs (in gray), and (c) expanded clones unique in CSF (in orange). (**C**) Consensus CDR3 sequences detected in CSF of HAM/TSP patients (*n* = 9). Each consensus sequence in 39 clusters is shown, and a consensus CDR3 motif from exclusively conserved among clusters is visualized with WebLogo.

Since disease-specific TCR clonotypes were not previously identified in PBMCs of HAM/TSP patients (14), we investigated whether there are any specific clonotypes shared across CSF compartments. We first compared the profile of TCR repertoires using identical CDR3 amino acid sequences in paired PBMCs and CSF across HAM/TSP patients. A heatmap analysis shows the frequency of the expanded clonotypes (≥ 8 UMIs) having identical CDR3 amino acid sequences shared across HAM/TSP patients in both PBMCs and CSF (Figure 3A). While shared TCR clonotypes between CSF and PBMCs were observed within each patient, there were no shared identical TCR β clonotypes across both CSF and PBMCs of patients with HAM/TSP (Figure 3A). These results indicate that TCR repertoire signatures can be identified that are unique to individual patients in CSF as well as PBMCs.

Since TCR clonal repertoires in CSF were not shared across patients, we next analyzed the TCR repertoire relatedness in the CSF of the 9 HAM/TSP patients using phylogenetic tree cluster analysis with respect to CDR3 amino acid sequence similarities. Figure 3B shows the phylogenetic tree analysis with 2 layers using expanded TCR β clonotypes with ≥ 8 UMIs (total of 240 clones) in CSF of all HAM/TSP patients. Sequence data for generating the tree are provided in Supplemental Table 1. The phylogenetic tree contains 2 layers to identify HAM/TSP patients (inner layer) and expanded clone groups in CSF of HAM/TSP patients (outer layer). This approach performed an all-versus-all pairwise comparison of clonotypes detected for this group of patients or a group of expanded clones in CSF and computed a set of clonotype frequencies, the diversity of overlap, and the geometric mean for the overlap. These metrics were then used to create hierarchical clustering from samples, producing a sample-level and expanded clone group–level dendrogram (Figure 3B). Upon visualization of this dendrogram, branch lengths described the distance between repertoires (longer branch length indicates greater dissimilarity per repertoire relatedness by CDR3 amino acid sequence) and node size increase in accordance with read counts. The phylogenetic tree demonstrated that a total of 39 clusters that shared common CDR3 amino acid motifs by sequence similarity were identified in the CSF of these 9 HAM/TSP patients (Figure 3B), suggesting the existence of a TCR β repertoire–level signature in HAM/TSP CSF. The expanded TCR β clonotypes in CSF of HAM/TSP patients were distributed widely in the phylogenetic tree, but some clusters — such as clusters 8, 12, and 34 — contained the expanded TCR β clonotypes detected from 5 HAM/TSP patients (inner layer in Figure 3B). These results suggest that there might be relatedness of CDR3 sequences among CSF of some HAM/TSP patients. In addition, the outer layer of the dendrogram represents the expanded clonotypes of CSF classified into 3 groups based on the overlap of expanded TCR β clonotypes between CSF and PBMCs: (a) expanded clones with ≥ 8 UMIs shared by CSF and PBMCs (Figure 3B, yellow colors in the outer layer); (b) clones expanded in CSF but detected at singleton or 2 ≤ UMIs < 8 in PBMCs (Figure 3B, gray colors in the outer layer); and (c) expanded clones unique in CSF (Figure 3B, orange colors in the outer layer). We also examined whether expanded clones shared by CSF and PBMCs or unique clones in CSF form a cluster; however, the expanded clone groups appeared distributed in the dendrogram, and no predominant clusters were detected (outer layer in Figure 3B). In addition, TCR clonotypes expanded in CSF were not distinguishable from the expanded TCR clonotypes shared by PBMCs and CSF by the analysis.

Based on the cluster of the dendrogram, the consensus sequences of the expanded TCR β clonotypes in CSF were aligned and summarized with the number of detected clones and the percentage of detected HAM/TSP patients (Figure 3C). The alignment of CDR3 sequences demonstrated that there were some similarities of CDR3 sequences between some clusters, while various CDR3 amino acid motifs were observed in the consensus sequences of each cluster. In 56% of HAM/TSP patients, the expanded TCR β clonotypes in CSF were detected in cluster 8, 12, or 34 (Figure 3C). Interestingly, cluster 7 and cluster 38 had a same motif PGL at positions 5–7, which were detected in 33% and 44% of HAM/TSP patients, respectively (Figure 3C). Cluster 3 and cluster 14 also had the same SLG motif at positions 8–10 in the center of the CDR3 region, and this similarity was detected in 33% and 44% of HAM/TSP patients, respectively (Figure 3C). When all clusters were compared for relatedness, a highly conserved amino acid glycine (G) at positions 10–12 of the CDR3 region was observed across the CSF repertoires of patients with HAM/TSP, in which 5 clones from 3 patients (33%) were identified. Collectively, these results demonstrate that TCR β clonotypes of expanded clones in CSF were unique to each patient; however, clusters with similar CDR3 motifs across HAM/TSP patients could be identified.

### Comparison of TCR repertoires among Tax11-19–specific CD8^+^ T cells, enriched CD8^+^ T cells, and peripheral blood in HAM/TSP patients.

**Figure 4:**
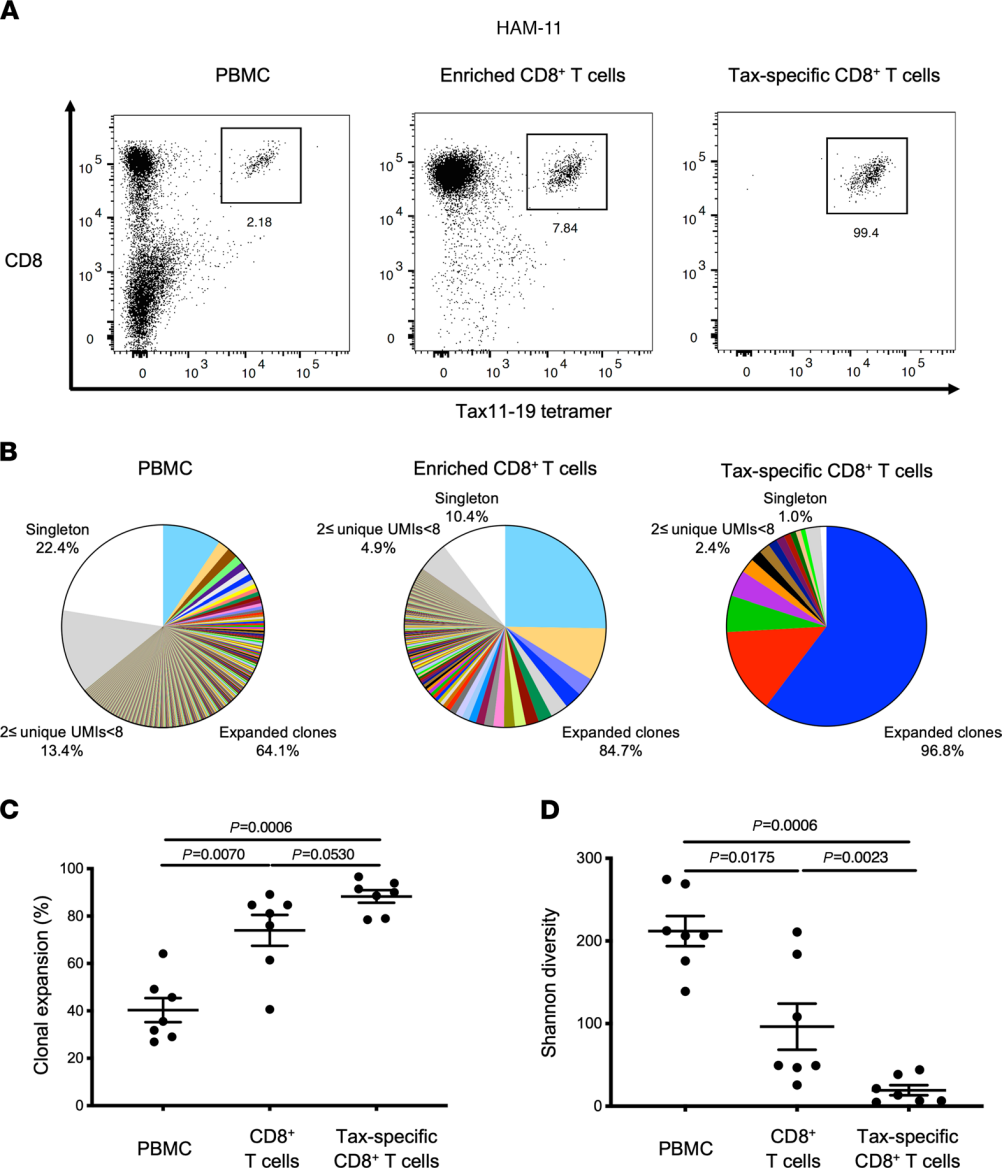
TCR clonal expansion of TCR β repertoire in PBMCs, enriched CD8^+^ T cells, and sorted Tax11-19–specific CD8^+^ T cells of HAM/TSP patients with HLA-A*0201. (**A**) Tax tetramer staining in CD3^+^ T cells of PBMCs (left), enriched CD8^+^ T cells (center), and sorted Tax11-19–specific CD8^+^ T cells (right) in a HAM/TSP patient (HAM-11). (**B**) Representative pie charts of T cell clonal expansion in PBMCs, enriched CD8^+^ T cells, and sorted Tax11-19–specific CD8^+^ T cells of a HAM/TSP patient (HAM-11). TCR β clonal expansion was analyzed by using the frequency of clones ≥ 8 UMIs (colored wedges), clones with 2 ≤ UMIs < 8 (gray), and singletons (white). In the group of expanded clones, each wedge represents a unique clonotype with a defined CDR3 sequence, and the same clone shared among PBMCs, CD8^+^ T cells, and Tax11-19–specific CD8^+^ T cells is visualized in the same color. (**C**) Comparison of TCR clonal expansion by frequency of clones ≥ 8 UMIs among PBMCs, enriched CD8^+^ T cells, and sorted Tax11-19–specific CD8^+^ T cells in HAM/TSP patients with HLA-A*0201 (*n* = 7) using the Friedman test’s post hoc test. Data represent mean ± SEM. (**D**) Comparison of Shannon diversity among PBMCs, enriched CD8^+^ T cells, and sorted Tax11-19–specific CD8^+^ T cells in HAM/TSP patients (*n* = 7) using the Friedman test’s post hoc test. Data represent mean ± SEM.

Since Tax protein has been shown to be an immunodominant HTLV-1 antigen recognized by HTLV-1–specific CTLs (5), we wished to determine if public TCR clonotypes in Tax-specific CTLs could be defined. To identify TCR clonotypes in HTLV-1 Tax11-19–specific CD8^+^ T cells, we examined and compared TCR repertoires in HAM/TSP PBMCs, in enriched CD8^+^ T cells, and sorted Tax11-19–specific CD8^+^ T cells from 7 HLA-A*0201^+^ HAM/TSP patients. Tax11-19–specific CD8^+^ T cells were detected, with Tax11-19/HLA-A*0201–specific tetramers in PBMCs of the 7 HAM/TSP patients at frequencies that ranged from 1.21% to 12.70% in CD8^+^ T cells (Table 1). Figure 4A shows representative dot plots of Tax11-19–specific CD8^+^ T cells of HAM-11 PBMCs, enriched CD8^+^ T cells, and sorted Tax11-19–specific CD8^+^ T cells. After sorting, Tax11-19–specific CD8^+^ T cells were effectively enriched from 2.18% in PBMCs to 7.84% and 99.4% in the enriched CD8^+^ T cells and sorted Tax11-19–specific CD8^+^ T cells, respectively (Figure 4A). TCR repertoire analysis from these 3 populations was performed. Figure 4B shows a pie chart representation of the frequency of expanded TCR clonotypes from all 3 populations of HAM-11. A comparison of the distribution of TCR clonotypes in each group revealed that the most frequently expanded clonotypes in Tax11-19–specific CD8^+^ T cells were not the highest-ranking expanded clonotypes in either PBMCs or purified CD8^+^ T cells (Figure 4B). For example, the unique TCR β expanded clonotype depicted as blue in highly purified Tax11-19–specific CD8^+^ T cells (representing 60.32% of all clonotypes) could only be found as the seventh most expanded clonotype in PBMCs (0.99% of all PBMC clonotypes) and the fourth most expanded clonotype in purified CD8^+^ T cells (2.81% of all CD8^+^ T cell clonotypes) (Figure 4B and Supplemental Table 2). Likewise, the second most expanded clonotype in Tax11-19–specific CD8^+^ T cells (depicted in red, 13.79% of all clones) was rarely detected in PBMCs or purified CD8^+^ T cells at 17th rank (0.37% of all PBMC clonotypes) and at 17th rank (0.80% of all CD8^+^ T cell clonotypes), respectively (Figure 4B). Conversely, the most frequently detected clonotype in PBMCs (9.38% of all PBMC clonotypes; Figure 4B, depicted in light blue) was detected at a higher frequency in purified CD8^+^ T cells (25.27% of all CD8^+^ T cell clonotypes) but was only at the 20th rank of most abundant expanded clonotype in Tax11-19–specific CD8^+^ T cells (0.15% of all Tax11-19–specific CD8^+^ T cell clonotypes; Figure 4B and Supplemental Table 2). The complete analysis of all 7 HLA-A*0201^+^ HAM/TSP patients demonstrated that the most frequently expanded clonotypes in Tax11-19–specific CD8^+^ T cells were not always detected at the higher ranks in PBMCs and CD8^+^ T cells of HAM/TSP patients (Supplemental Figure 3). Figure 4C shows the frequency of expanded T cell clonotypes in PBMCs, enriched CD8^+^ T cells, and sorted Tax11-19–specific CD8^+^ T cells of HAM/TSP patients. As expected, sorted Tax11-19–specific CD8^+^ T cells showed the highest degree of TCR clonal expansion compared with PBMCs (*P* = 0.0006) and CD8^+^ T cells (*P* = 0.0530) (Figure 4C). Applying the Shannon diversity, a significantly lower diversity within the TCR repertoire was observed in sorted Tax11-19–specific CD8^+^ T cells compared with PBMCs (*P* = 0.0006) and CD8^+^ T cells (*P* = 0.0023) (Figure 4D). These results demonstrate that antigen-specific T cells have the most expanded TCR clonal repertoire compared with PBMCs or enriched CD8^+^ T cells, suggesting that multiple clones can recognize HTLV-1 Tax11-19 in association with HLA-A*0201. Importantly, since expanded TCR clonotypes in Tax11-19–specific CD8^+^ T cells were not always detected at the highest rank in PBMCs or CD8^+^ T cells, analysis of antigen-specific TCR clonotypes detectable in peripheral blood of HAM/TSP patients may not always reflect antigen-specific, immunodominant responses.

### TCR β clonotypes of expanded clones in HLA-A*0201–restricted Tax11-19–specific CD8^+^ T cells are unique to each individual but have conserved CDR3 motifs.

**Figure 5:**
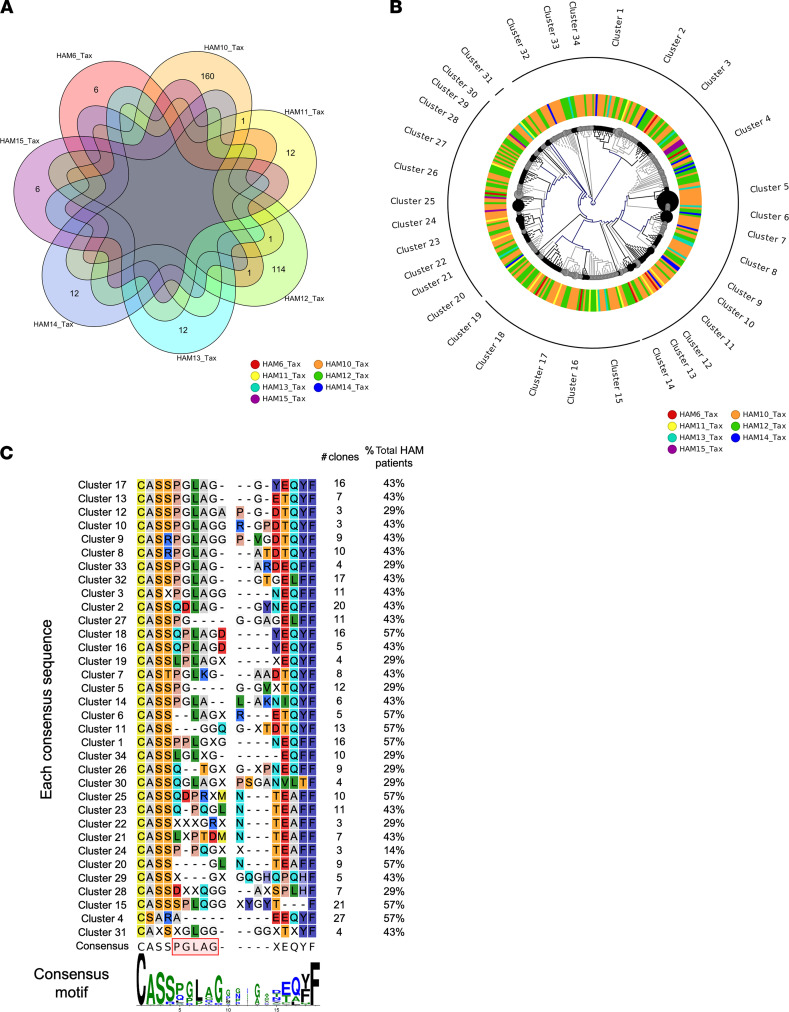
TCR β repertoire relatedness of expanded clonotypes in Tax11-19–specific CD8^+^ T cells of HAM/TSP patients with HLA-A*0201. (**A**) Relationship of the exact CDR3 amino acid sequences of TCR β repertoire in Tax11-19–specific CD8^+^ T cells across 7 HAM/TSP patients with HLA-A*0201 using a Venn diagram. (**B**) Phylogenetic tree analysis of the CDR3 amino acid sequences of TCR β repertoire in Tax11-19–specific CD8^+^ T cells of HAM/TSP patients with HLA-A*0201 (*n* = 7). Branch lengths represent the distance between repertoires, and node size is shown according to read counts. Color-coded layer represents each HAM/TSP patient with HLA-A*0201. (**C**) Consensus CDR3 sequences detected in Tax-specific CD8^+^ T cells of HAM/TSP patients with HLA-A*0201 (*n* = 7). Each consensus sequence in 34 clusters is shown, and a consensus CDR3 motif from predominantly conserved among clusters is visualized with WebLogo.

To determine whether there are public TCR clonotypes in HLA-A*0201–restricted Tax-specific CTLs of HAM/TSP patients, we compared the expanded clones with at least 8 UMIs in Tax11-19–specific CD8^+^ T cells by identical CDR3 amino acid sequences. In Figure 5A, a Venn diagram depicts the overlap between the expanded CDR3 sequences detected by each HAM/TSP patient and demonstrates no common TCR β clonotypes detected in all 7 patients comparing exact CDR3 amino acid sequences. Most TCR β clonotypes were unique to each individual patient, while a few identical CDR3 sequences were shared only between 3 pairs of HAM/TSP patients (HAM-6 and HAM-12, HAM-10 and HAM-11, and HAM-10 and HAM-12; Figure 5A). These results indicate that HAM/TSP patients known to recognize the same immunodominant HTLV-1 Tax11-19 peptide in association with the HLA-A*0201 allele use a private TCR repertoire in which no identical shared TCR β clonotypes could be detected.

We next examined whether there is any CDR3 sequence relatedness in the Tax-specific CTLs. Phylogenetic analysis was performed using expanded TCR β clonotypes with more than 8 UMIs (total of 336 clones) in Tax11-19–specific CD8^+^ T cells of 7 HLA-A*0201^+^ HAM/TSP patients (Figure 5B). Sequence data for constructing the tree are provided in Supplemental Table 3. As described above, this approach performed an all-versus-all pairwise comparison of clonotypes detected for a list of patients with HAM/TSP in Tax11-19–specific CD8^+^ T cells, and this comparison was then used to cluster samples hierarchically, generating a sample-level dendrogram (Figure 5B). The phylogenetic analysis showed the similarities in the TCR β repertoires and the identification of 34 clusters that shared common CDR3 motifs by sequence similarity in Tax11-19–specific CD8^+^ T cells of HAM/TSP patients (Figure 5B). Interestingly, some clusters, such as clusters 1, 4, 6, 11, 15, 18, 20, and 25, contained the expanded TCR β clonotypes detected from 4 of 7 HAM/TSP patients (Figure 5B), suggesting that TCR β repertoire–level signatures and similarities could be identified in Tax-specific CTLs of HAM/TSP patients. The consensus sequences of the expanded TCR β clonotypes in Tax11-19–specific CD8^+^ T cells were aligned and compared among all of the clusters (Figure 5C). The alignment of TCR β CDR3 sequences in Tax11-19–specific CD8^+^ T cells from all of the clusters showed a highly conserved PGLAG amino acid sequence motif at the position 4–8 of the CDR3 region, which was detected in 46 clones (14%) and 4 HAM/TSP patients (57%). Our results demonstrate that patients having the same HLA-A type used private TCR repertoires to recognize HTLV-1 Tax11-19 peptide; however, there was a conserved CDR3 motif (PGLAG) in HTLV-1 Tax–specific CTLs across HLA-A*0201^+^ HAM/TSP patients.

### TCR β expanded clonotypes of HTLV-1–specific CD8^+^ T cells are enriched in CSF of a HAM/TSP patient.

**Figure 6:**
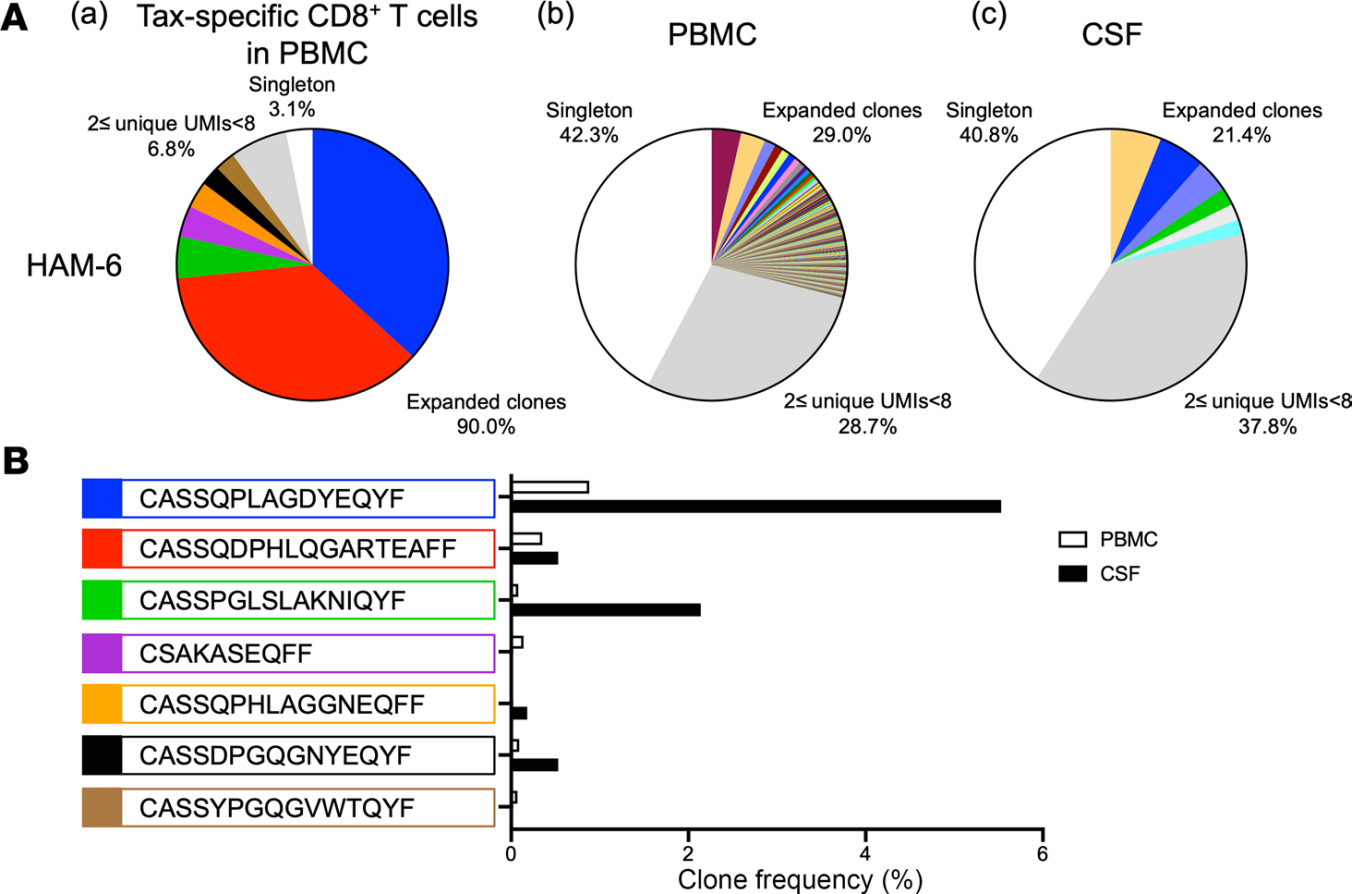
Enrichment of Tax-specific TCR β expanded clonotypes in CSF of a HAM/TSP patient. (**A**) T cell clonal expansion of sorted Tax11-19–specific CD8^+^ T cells from PBMCs (left), PBMCs (middle), and CSF (right) of a HAM/TSP patient (HAM-6). TCR β clonal expansion was analyzed by using the frequency of clones ≥ 8 UMIs (colored wedges), clones with 2 ≤ UMIs < 8 (gray), and singletons (white). In the group of expanded clones, each wedge represents a unique clonotype with a defined CDR3 sequence, and the same clone shared among Tax11-19–specific CD8^+^ T cells, PBMCs, and CSF cells is visualized in the same color. (**B**) Frequencies of 7 TCR β expanded clonotypes of Tax11-19–specific CD8^+^ T cells in PBMCs and CSF of a HAM-TSP patients (HAM-6). Each clonotype is visualized in the same color with the wedge of the pie chart in **A**.

Since it has been reported that Tax-specific CTLs are elevated both in PBMCs and CSF of HAM/TSP patients (6), expanded CTL clones in peripheral blood are thought to cross the blood-brain barrier and cause CNS inflammation (7). However, it remains unclear whether the expanded CTL clones in CSF are derived from the peripheral blood or are expanded compartmentally in the CNS. We had the opportunity to obtain the CSF from 1 HLA-A*0201^+^ HAM/TSP patient (HAM-6) from which we could compare the TCR β clonally expanded repertoires of paired samples of Tax11-19–specific CD8^+^ T cells in PBMCs, total PBMCs, and total CSF cells (Figure 6A; left, middle, and right, respectively). In Tax11-19–specific CD8^+^ T cells isolated from peripheral blood, 7 TCR expanded clonotypes were detected (Figure 6A, left, and Table 3). Two TCR β expanded clonotypes identified at the first (depicted in blue) and third rank (depicted in green) in Tax11-19–specific CD8^+^ T cells (CASSQPLAGDYEQYF and CASSPGLSLAKNIQYF) were also detected at the second and the fourth rank in the expanded clonotypes of CSF, respectively (Figure 6A, right, and Table 3). The second most expanded TCR clonotype (depicted in red) from Tax11-19–specific CD8^+^ T cells (CASSQDPHLQGARTEAFF) was present (2 ≤ UMIs < 8) at the 23rd rank in CSF of HAM-6 (0.53% of all CSF clones) but did not meet our definition of expanded clones ≥ 8 UMIs (Figure 6A, right, and Table 3). The 2 TCR β sequences of Tax11-19–specific CD8^+^ T cells in PBMCs (CASSQPLAGDYEQYF and CASSPGLSLAKNIQYF) were also detected at the sixth (0.88% of all PBMC clones) and the 69th (0.08% of all PBMC clones) rank, respectively, in the expanded clonotypes of PBMCs (Figure 6A, middle, and Table 3). Importantly, when comparing the frequency of the 7 TCR β sequences detected in the PBMCs and the CSF of HAM-6, these 2 TCR β sequences of Tax11-19–specific CD8^+^ T cells (CASSQPLAGDYEQYF and CASSPGLSLAKNIQYF) were detected more in the CSF compared with the peripheral blood (Figure 6B). Collectively, our results suggest that a subset of HTLV-1 Tax–specific TCR β clonotypes was clonally expanded in peripheral blood and was subsequently infiltrated, becoming highly enriched in the CSF compartment.

## Discussion

The TCR repertoire is shaped by antigen engagement and altered in the context of the disease (10). A more complete characterization of TCR repertoires may provide disease-specific signatures, including specific TCR clonotypes or oligoclonal skewed TCR repertoires in immune-mediated disorders, such as chronic viral infection and autoimmune disease. Furthermore, exploring the TCR repertoire signatures focused on expanded clones particularly in the CNS may clarify local antigen-driven T cell activation and pathologically related TCR sequences in neuroinflammatory disorders. CSF contains predominantly T cells (approximately 80%) and has unique immunoregulatory roles including control of T cell entry and migration within the CNS, and it may be associated with both protective and pathological immune responses (16, 27). To explore disease-related TCR repertoire signatures in a neurologic disorder, we investigated the TCR repertoire from CSF cells in patients with HAM/TSP, a chronic inflammatory neurologic disease associated with HTLV-1 infection. TCR β repertoires of HAM/TSP patients in both PBMCs and CSF were more clonally expanded compared with those of NDs. Moreover, an overlap of TCR β clonotypes of expanded clones was identified between peripheral blood and CSF in HAM/TSP patients. By comparison, we also had the opportunity to obtain CSF and matched PBMCs from healthy, HTLV-1–seronegative control individuals in which expanded TCR clonotypes were much less or undetectable in CSF, although — as in HAM/TSP — individual variations of clonal expansion were also observed in PBMCs of NDs. The absence of clonally expanded TCR β clonotypes in NDs might be associated with low number of activated T cells found in CSF of NDs, compared with HAM/TSP patients (28). The degree of TCR β clonal expansions in PBMCs was correlated with effector T cells and HTLV-1 PVL in the periphery of HAM/TSP patients. HAM/TSP patients are known to have a higher PVL compared with asymptomatic carriers, and this comparison is associated with increased phenotypically defined effector CD8^+^ T cell subsets and HTLV-1 Tax–specific CD8^+^ T cells (26, 29) where chronic viral antigen stimulations have been shown to drive clonal expansion in both infected CD4^+^ T cells and HTLV-1–specific CD8^+^ T cells (30). These previous reports support results in this study that demonstrate higher TCR β clonal expansions in peripheral blood, as well as in CSF in HAM/TSP compared with NDs. Collectively, these results strongly suggest that TCR repertoires in CSF of HAM/TSP patients may provide a local disease-specific T cell immune signature.

Although our results show that there was no identical CDR3 sequence in CSF across all HAM/TSP patients, TCR β repertoire clusters were detected using phylogenetic tree cluster analysis. Approximately 80% of the expanded TCR clones in CSF of HAM/TSP patients were derived from expanded clones in PBMCs, while 9% of clones appeared to be uniquely expanded in CSF of these patients. The expanded T cell clones shared by CSF and PBMCs compared with those expanded TCR clones unique to CSF may reflect a variation in the antigens recognized in each compartment and the capacity to clonally expand at each site. Indeed, TCR repertoire analyses in MS patients have shown that expanded T cell clones shared by the CNS and periphery are predominantly observed in the peripheral CD8^+^ T cell compartment (18, 31), while expanded T cell clones unique to the CNS are found in peripheral CD4^+^ T cells (31). In our study, no predominant cluster shared by CSF and PBMCs or unique to CSF was identified in HAM/TSP patients using phylogenetic tree analysis. Since T cell clones exclusively expanded in the CNS are considered to reflect local inflammation (18, 31), particularly in an immunopathologically mediated disease such as HAM/TSP, identification of TCR repertoire signatures that are disease and/or pathogen specific would be important.

Since T cells recognize foreign peptides through engagement with TCR and peptide-MHC (8), defining a pathogen-reactive TCR repertoire signature requires donor HLA haplotype information to filter the majority of TCRs that would not recognize a pathogen in the context of a particular HLA (10, 32). However, public TCR sequences, which are shared among multiple individuals in response to the same antigenic epitopes, have been extensively observed to a variety of viruses, including EBV, cytomegalovirus, herpes simplex virus, and HIV, and have been associated with favorable biological outcomes (33). However, more recently, TCR analyses based on HTS have demonstrated that there can be diversity of TCR sequences that recognize even the same epitopes (34–36). Consistent with these studies, our results show that HLA-A*0210–restricted Tax11-19–specific CD8^+^ T cells from HLA-A*0201^+^ HAM/TSP patients used private TCR β sequences to recognize the same immunodominant HTLV-1 Tax antigen. Public TCR β sequences (as defined by identical CD3 sequences from expanded TCR clonotypes) were rarely identified in Tax11-19–specific CD8^+^ T cells in 7 HAM/TSP patients (Figure 5A). However, phylogenetic tree cluster analysis showed that TCR β repertoires of these Tax11-19–specific CD8^+^ T cells had similar sequences and conserved motifs, such as PGLAG, in the CDR3 region. These observations are consistent with previous reports on the TCR analysis of the CDR3 region of Tax11-19–specific CD8^+^ T cells in HLA-A*0201^+^ HAM/TSP patients that showed conserved motifs of 3 amino acid sequence (PG-G) (23) or 4 sequence (P/G-L-A/R-G) (22). Moreover, crystal structure analysis revealed a corner on the apex of the TCR β CDR3 loop (GLAG) that was shown to insert into a hydrophobic pocket formed by the α-1 helix of HLA-A2 and the side chain of the Tyr residue at position 8 in the Tax11-19 peptide (22). These observations support that the motif of PGLAG is important for recognition of HTLV-1 Tax protein by antigen-specific CD8^+^ T cells of HAM/TSP patients. Since Tax-specific CTLs are considered to play a key role in the pathogenesis of HAM/TSP (7), further research is needed to examine whether CD8^+^ T cells with these specific motifs have protective or deleterious effects against HTLV-1 infection.

In the present study, we also had the opportunity to obtain CSF cells from an HLA-A*0201^+^ HAM/TSP patient from which we identified HTLV-1–specific TCR β repertoires in this compartment. We demonstrated that TCR β clonotypes of Tax11-19–specific CD8^+^ T cells in the periphery were also clonally expanded and enriched in the CSF. This result is a direct demonstration that activated and clonally expanded HTLV-1–specific CD8^+^ T cells in peripheral blood infiltrate and become highly enriched in the CNS of HAM/TSP. Interestingly, the amino acid sequences of the 2 CSF expanded TCR β clonotypes (CASSQPLAGDYEQYF at positions 6–9 and CASSPGLSLAKNIQYF at positions 5–7; Figure 6B and Table 3) were similar to the PGLAG motif of the consensus sequence identified from HLA-A*0201–restricted Tax11-19–specific CD8^+^ T cells in the peripheral blood of HLA-A*0201^+^ HAM/TSP patients (Figure 5C). Collectively, these results suggest that these TCR β clonotypes may be associated with the HTLV-1– or disease-specific TCR repertoire signatures. Analysis of TCR repertoires in other diseases associated with antigen-specific T cell responses has also shown enrichment of antigen-specific CD8^+^ T cells in sites of inflammation that were present in peripheral blood. These include enrichment of EBV-reactive CD8^+^ T cells in CSF of MS patients (17), influenza-specific CD8^+^ T cells in human lungs (37), and islet-reactive CD8^+^ T cells in the pancreas of type 1 diabetes patients (38). Analysis of antigen-specific TCR repertoire signatures in the periphery could therefore be used to track and identify pathogens in sites of local inflammation. Recently, several TCR databases have been developed to annotate individual TCR repertoires to TCR sequences associated with known antigens, pathogens, and pathologies based on published literature (32). We annotated TCR β repertoires of expanded clones obtained from CSF cells and Tax11-19–specific CD8^+^ T cells of HAM/TSP patients to representative TCR databases using VDJdb (36) and McPAS-TCR (39), although identical TCR β clonotypes were rarely identified (4 clonotypes in VDJdb and 0 in McPAS-TCR). The TCR database is presumed to need larger numbers of TCR sequences for clinical applications.

In conclusion, we have shown that TCR β repertoires of HAM/TSP patients are highly expanded in CSF and contain both TCR clonotypes shared with PBMCs and uniquely enriched clonotypes within the CSF. Analysis of the TCR β repertoire of HLA-A*0201–restricted Tax11-19–specific CD8^+^ T cells demonstrated the use of private TCR β sequences for the recognition of antigen and identified conserved motifs in the CDR3 region. Moreover, TCR β clonotypes of expanded clones in HTLV-1–specific CD8^+^ T cells in the periphery were also expanded and enriched in the CSF. Exploring the TCR repertoire of CSF and antigen-specific T cells may provide a TCR repertoire signature in virus-associated neurologic disorders. Further analysis and technologies, such as the paired analysis of the 2 TCR chains (αβ, γδ) and single-cell isolation technologies, can serve to highlight rare and functionally important populations in the immune system of the CNS that may lead to a better understanding of disease pathogenesis.

## Methods

### Subjects.

Twenty subjects consisting of 15 HAM/TSP patients and 5 NDs were included for sequencing of TCR β chain repertoires by HTS. HAM/TSP patients were defined by World Health Organization criteria, and HTLV-1–uninfected healthy volunteers screened at the NIH Clinical Center (Bethesda, Maryland, USA) were evaluated as NDs. Subsets of HAM/TSP patients were used for specific studies. For analysis of paired PBMCs and CSF cells, peripheral blood and CSF samples were collected on the same day from 9 HAM/TSP patients and 5 NDs. For analysis of HTLV-1–specific CD8^+^ T cells, peripheral blood was obtained from 7 HLA-A*0201^+^ HAM/TSP patients. Subject characteristics including sex, age, ethnicity, disease status, and HLA-A type are listed in Table 1. PBMCs were isolated from the peripheral blood according to the Ficoll-Hypaque density gradient centrifugation method according to the manufacturer’s instructions and then cryopreserved in liquid nitrogen until the date of use. CSF samples were obtained by nontraumatic lumbar puncture at volumes greater than 20 mL; subsequently, cells were collected by centrifugation at 400*g* for 10 minutes at 4°C. The CSF cells were cryopreserved in RNAlater (Invitrogen, Thermo Fisher Scientific) solution for TCR sequencing.

### HTLV-1 PVL.

HTLV-1 PVL from HAM/TSP patients was quantified based on the percentage of HTLV-1 *tax* gene detection in PBMCs by using the droplet digital PCR technique (Bio-Rad), as previously reported (25). DNA was extracted from the PBMCs and CSF cell pellets using a DNeasy Blood and Tissue kit (Qiagen) according to the manufacturer’s instructions. Primers and probes specific for HTLV-1 *tax* and human ribonuclease P protein subunit 30 (*RPP30*) were used (25). All samples were tested in duplicate, and PVL was reported as the average of the 2 measurements.

### Flow cytometry and sorting.

For flow cytometric analysis, EDTA-treated whole blood from NDs and HAM/TSP patients were stained with CD3, CD4, CD8, CD27, CD45, and CD45RA (all from BD Biosciences), as well as T cell subtypes, were defined as effector/memory (CD27^–^CD45RA^–^) and effector (CD27^–^CD45RA^+^) subsets in CD4^+^ or CD8^+^ T cells as previously described (28, 40). All flow cytometric analyses were performed using a LSR II (BD Biosciences). For staining of HTLV-1 Tax–specific CD8^+^ T cells in PBMCs, Tax11-19/HLA-A*0201 tetramer (provided by NIH Tetramer Core Facility, Atlanta, Georgia, USA) was used. For sorting of Tax11-19–specific CD8^+^ T cells, after CD8^+^ T cells were enriched from 7 HAM/TSP patients with HLA-A*0201 using human CD8^+^ T cell isolation kit (Miltenyi Biotec), Tax11-19–specific CD8^+^ T cells were sorted from the enriched CD8^+^ T cells using FACSAria II (BD Biosciences). The purity of enriched CD8^+^ T cells and Tax11-19–specific CD8^+^ T cells was 82.0%–96.3% and 93.0%–100.0%, respectively. All the data were analyzed using FlowJo 10.6 software. All antibodies used for flow cytometry are provided in Supplemental Table 4.

### TCR β library preparation.

HTS for TCR β chain was performed on PBMCs, enriched CD8^+^ T cells, sorted Tax11-19–specific CD8^+^ T cells and CSF cells. The TCR β chain was amplified from 2 × 10^6^ cells of PBMCs and enriched CD8^+^ T cells. The sorted Tax11-19–specific CD8^+^ T cells and CSF cells were used at 4 × 10^4^ to 5 × 10^5^ cells and 1 × 10^4^ to 2.5 × 10^5^ cells, respectively, for the amplification of TCR β chain. Total RNA was extracted from PBMCs and enriched CD8^+^ T cells using a miRNA Mini Kit (Qiagen), and RNA from sorted Tax11-19–specific CD8^+^ T cells and CSF cells was extracted using a RNeasy Micro kit (Qiagen) following the manufacturer’s instructions. The rearranged TCR β repertoires were amplified using previously described methods (14). Briefly, cDNA synthesis was performed using the anchored switch 5′ rapid amplification of cDNA ends (5′ RACE) PCR-based primer combined with a unique molecular barcode that consists of 10 random nucleotide sequences. Nested PCR was performed using a pair of primers specific for the TCR constant region and 5′ RACE region, which contains a sample index and Illumina adapter sequences. Final PCR product was purified, and the quality of TCR library was analyzed using a Bioanalyzer (Agilent Technologies).

### TCR β repertoire sequencing and bioinformatics analysis.

TCR library were sequenced on the Miseq platform with a 150 bp paired-end run (Illumina). After quality filtering, raw sequence reads were then processed using the MiGEC software pipeline with default parameter (15) for CDR3 extraction and VDJ gene segment alignment to the human TCR β germline sequences based on IMGT database. We conducted further analysis using VDJtools (41) and tcR (42), which allowed applying postanalysis of repertoire sequencing data and generated basic sample statistics. Read counts, number of clonotypes, diversity estimations, and repertoire overlap analysis within each sample or across samples were evaluated and visualized using these software tools. To evaluate the TCR β clonal expansion, TCR β clones in each subject were classified into one of the 3 groups: singletons (1 UMI), clones (2 ≤ UMIs < 8), and clones ≥ 8 UMIs, as previously described based on coefficients of variation analyses for each clonotype by the average number of UMIs observed across technical triplicates (14). Those clones having at least 8 UMIs were used to perform phylogenetic tree cluster analyses with respect to CDR3 amino acid sequence similarities across samples to determine clonal relatedness using CLCbio workbench (Qiagen). Consensus sequence data from the cluster analysis were then visualized with WebLogo (43).

### Statistics.

The Mann-Whitney *U* test was used to compare clonal expansion both in PBMCs and CSF between HAM/TSP patients and NDs. The Friedman test’s post hoc test was used to compare clonal expansion and Shannon diversity index among PBMCs, enriched CD8^+^ T cells, and Tax11-19–specific CD8^+^ T cells in HAM/TSP patients with HLA-A*0201. Wilcoxon’s signed rank test was used to compare HTLV-1 PVL between PBMCs and CSF in HAM/TSP patients. Spearman’s rank correlation test was used to compare effector/memory and effector cells both in CD4^+^ and CD8^+^ T cells or HTLV-1 PVL in PBMCs with clonal expansion in PBMCs. All statistical analysis was performed using Prism 8 (GraphPad software) or R software (R version 3.5.1).

### Study approval.

All samples from HAM/TSP patients and NDs were collected under protocol (National Institute of Neurological Disorders and Stroke protocol nos. 98-N-0047 and 13-N-0149). All subjects gave written informed consent before inclusion in accordance with the Declaration of Helsinki, and the study was reviewed and approval by the NIH IRB.

## Author contributions

SN designed the study, performed the experiments, analyzed the data, and wrote the manuscript. YEA performed the experiments, analyzed the data, and wrote the manuscript. KRJ performed the bioinformatic analysis and analyzed the data. MCM and NN performed the experiments. AE and JZ provided experimental and high-throughput sequencing support. JO provided clinical support. SJ supervised the project, interpreted the data, and wrote the manuscript.

## Supplementary Material

Supplemental data

## Figures and Tables

**Table 1:**
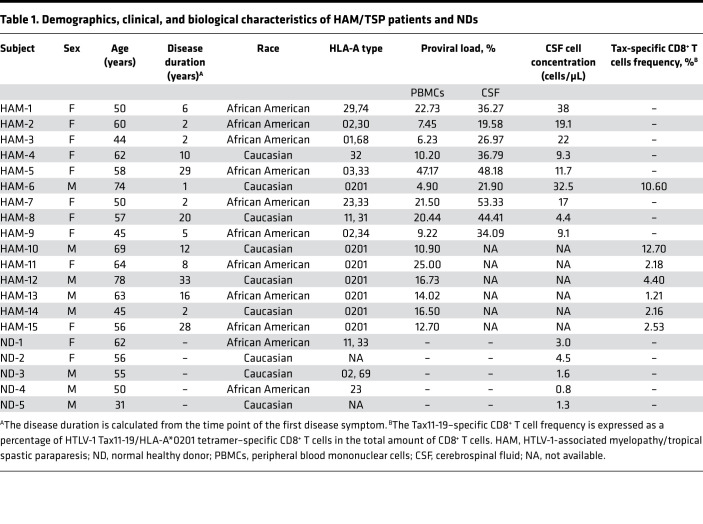
Demographics, clinical, and biological characteristics of HAM/TSP patients and NDs

**Table 2:**
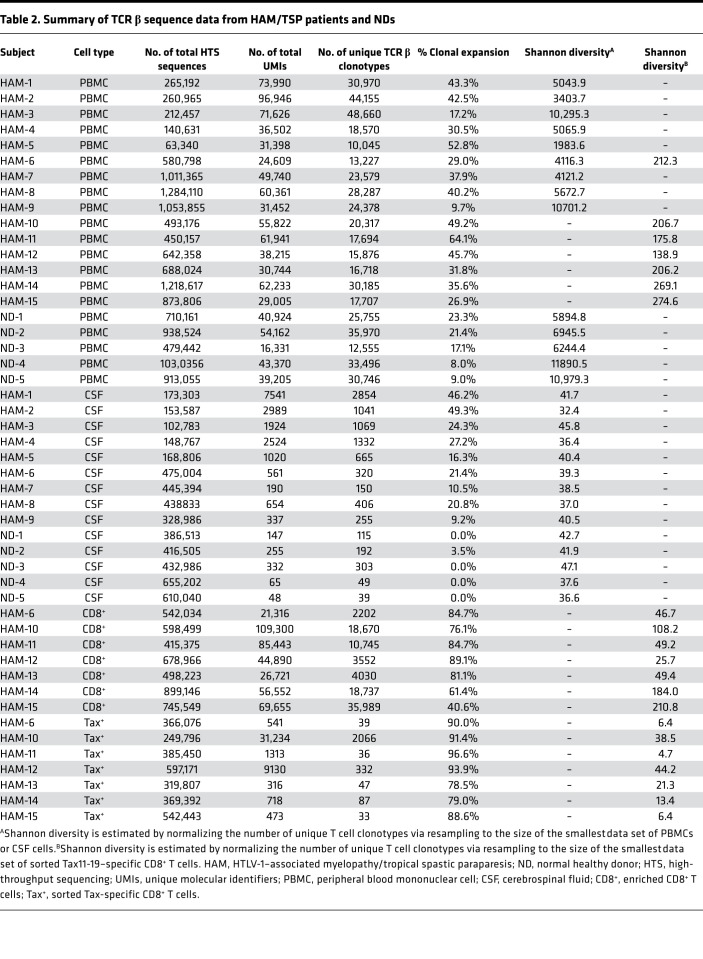
Summary of TCR β sequence data from HAM/TSP patients and NDs

**Table 3:**
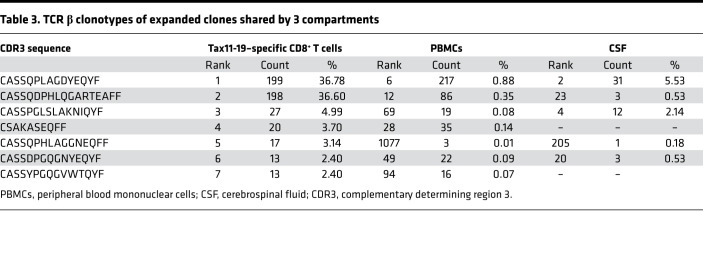
TCR β clonotypes of expanded clones shared by 3 compartments

## Version 1 02/22/2021

Electronic publication

## References

[1] Uchiyama T (1977). Adult T-cell leukemia: clinical and hematologic features of 16 cases. Blood.

[2] Osame M (1986). HTLV-I associated myelopathy, a new clinical entity. Lancet.

[3] Kubota R (1998). Demonstration of human T lymphotropic virus type I (HTLV-I) tax-specific CD8+ lymphocytes directly in peripheral blood of HTLV-I-associated myelopathy/tropical spastic paraparesis patients by intracellular cytokine detection. J Immunol.

[4] Yamano Y (2002). Correlation of human T-cell lymphotropic virus type 1 (HTLV-1) mRNA with proviral DNA load, virus-specific CD8(+) T cells, and disease severity in HTLV-1-associated myelopathy (HAM/TSP). Blood.

[5] Jacobson S (1990). Circulating CD8+ cytotoxic T lymphocytes specific for HTLV-I pX in patients with HTLV-I associated neurological disease. Nature.

[6] Nagai M (2001). Increased HTLV-I proviral load and preferential expansion of HTLV-I Tax-specific CD8+ T cells in cerebrospinal fluid from patients with HAM/TSP. Ann Neurol.

[7] Jacobson S (2002). Immunopathogenesis of human T cell lymphotropic virus type I-associated neurologic disease. J Infect Dis.

[8] Davis MM, Bjorkman PJ (1988). T-cell antigen receptor genes and T-cell recognition. Nature.

[9] Laydon DJ (2015). Estimating T-cell repertoire diversity: limitations of classical estimators and a new approach. Philos Trans R Soc Lond B Biol Sci.

[10] Attaf M (2015). αβ T cell receptors as predictors of health and disease. Cell Mol Immunol.

[11] Bianconi E (2013). An estimation of the number of cells in the human body. Ann Hum Biol.

[12] Krogsgaard M, Davis MM (2005). How T cells ‘see’ antigen. Nat Immunol.

[13] Rosati E (2017). Overview of methodologies for T-cell receptor repertoire analysis. BMC Biotechnol.

[14] Alves Sousa AP (2019). Comprehensive analysis of TCR-beta repertoire in patients with neurological immune-mediated disorders. Sci Rep.

[15] Shugay M (2014). Towards error-free profiling of immune repertoires. Nat Methods.

[16] Wilson EH (2010). Trafficking of immune cells in the central nervous system. J Clin Invest.

[17] Lossius A (2014). High-throughput sequencing of TCR repertoires in multiple sclerosis reveals intrathecal enrichment of EBV-reactive CD8+ T cells. Eur J Immunol.

[18] Salou M (2015). Expanded CD8 T-cell sharing between periphery and CNS in multiple sclerosis. Ann Clin Transl Neurol.

[19] Stangel M (2013). The utility of cerebrospinal fluid analysis in patients with multiple sclerosis. Nat Rev Neurol.

[20] Eiraku N (1998). Clonal expansion within CD4+ and CD8+ T cell subsets in human T lymphotropic virus type I-infected individuals. J Immunol.

[21] Ureta-Vidal A (2001). Human T cell leukemia virus type I (HTLV-I) infection induces greater expansions of CD8 T lymphocytes in persons with HTLV-I-associated myelopathy/tropical spastic paraparesis than in asymptomatic carriers. J Infect Dis.

[22] Saito M (2001). In vivo selection of T-cell receptor junctional region sequences by HLA-A2 human T-cell lymphotropic virus type 1 Tax11-19 peptide complexes. J Virol.

[23] Bourcier KD (2001). Conserved CDR3 regions in T-cell receptor (TCR) CD8(+) T cells that recognize the Tax11-19/HLA-A*0201 complex in a subject infected with human T-cell leukemia virus type 1: relationship of T-cell fine specificity and major histocompatibility complex/peptide/TCR crystal structure. J Virol.

[24] Nakagawa M (1995). HTLV-I-associated myelopathy: analysis of 213 patients based on clinical features and laboratory findings. J Neurovirol.

[25] Brunetto GS (2014). Digital droplet PCR (ddPCR) for the precise quantification of human T-lymphotropic virus 1 proviral loads in peripheral blood and cerebrospinal fluid of HAM/TSP patients and identification of viral mutations. J Neurovirol.

[26] Nagai M (2001). Increased activated human T cell lymphotropic virus type I (HTLV-I) Tax11-19-specific memory and effector CD8+ cells in patients with HTLV-I-associated myelopathy/tropical spastic paraparesis: correlation with HTLV-I provirus load. J Infect Dis.

[27] Ousman SS, Kubes P (2012). Immune surveillance in the central nervous system. Nat Neurosci.

[28] Enose-Akahata Y (2018). Immunophenotypic characterization of CSF B cells in virus-associated neuroinflammatory diseases. PLoS Pathog.

[29] Nagai M (1998). Analysis of HTLV-I proviral load in 202 HAM/TSP patients and 243 asymptomatic HTLV-I carriers: high proviral load strongly predisposes to HAM/TSP. J Neurovirol.

[30] Enose-Akahata Y (2017). Role of HTLV-1 Tax and HBZ in the Pathogenesis of HAM/TSP. Front Microbiol.

[31] Planas R (2018). Detailed characterization of T cell receptor repertoires in multiple sclerosis brain lesions. Front Immunol.

[32] Zvyagin (2019). An overview of immunoinformatics approaches and databases linking T cell receptor repertoires to their antigen specificity. Immunogenetics.

[33] Li H (2012). Determinants of public T cell responses. Cell Res.

[34] Qi Q (2016). Diversification of the antigen-specific T cell receptor repertoire after varicella zoster vaccination. Sci Transl Med.

[35] Glanville J (2017). Identifying specificity groups in the T cell receptor repertoire. Nature.

[36] Shugay M (2018). VDJdb: a curated database of T-cell receptor sequences with known antigen specificity. Nucleic Acids Res.

[37] Pizzolla A (2018). Influenza-specific lung-resident memory T cells are proliferative and polyfunctional and maintain diverse TCR profiles. J Clin Invest.

[38] Culina S (2018). Islet-reactive CD8^+^ T cell frequencies in the pancreas, but not in blood, distinguish type 1 diabetic patients from healthy donors. Sci Immunol.

[39] Tickotsky N (2017). McPAS-TCR: a manually curated catalogue of pathology-associated T cell receptor sequences. Bioinformatics.

[40] Schindler MK (2020). Haploinsufficiency of immune checkpoint receptor CTLA4 induces a distinct neuroinflammatory disorder. J Clin Invest.

[41] Shugay M (2015). VDJtools: unifying post-analysis of T cell receptor repertoires. PLoS Comput Biol.

[42] Nazarov VI (2015). tcR: an R package for T cell receptor repertoire advanced data analysis. BMC Bioinformatics.

[43] Crooks GE (2004). WebLogo: a sequence logo generator. Genome Res.

