# Supplementary material for: Immunopathogenic CSF TCR repertoire signatures in virus-associated neurologic disease
Source: JCI Insight. 2021 Feb 22;6(4):e144869. doi: 10.1172/jci.insight.144869 (PMC7934934; doi:10.1172/jci.insight.144869)
Supplement: Supplemental data [file jciinsight-6-144869-s145.pdf]

Immunopathogenic CSF TCR repertoire signatures in virus-associated neurologic disease.

Satoshi Nozuma[1], Yoshimi Enose-Akahata[1], Kory R. Johnson[2], Maria Chiara Monaco[1], Nyater Ngouth[1], Abdel Elkahloun[3], Joan Ohayon[4], Jun Zhu[5] and Steven Jacobson[1]

Supplemental materials

Supplemental figures: 3

Supplemental tables: 4

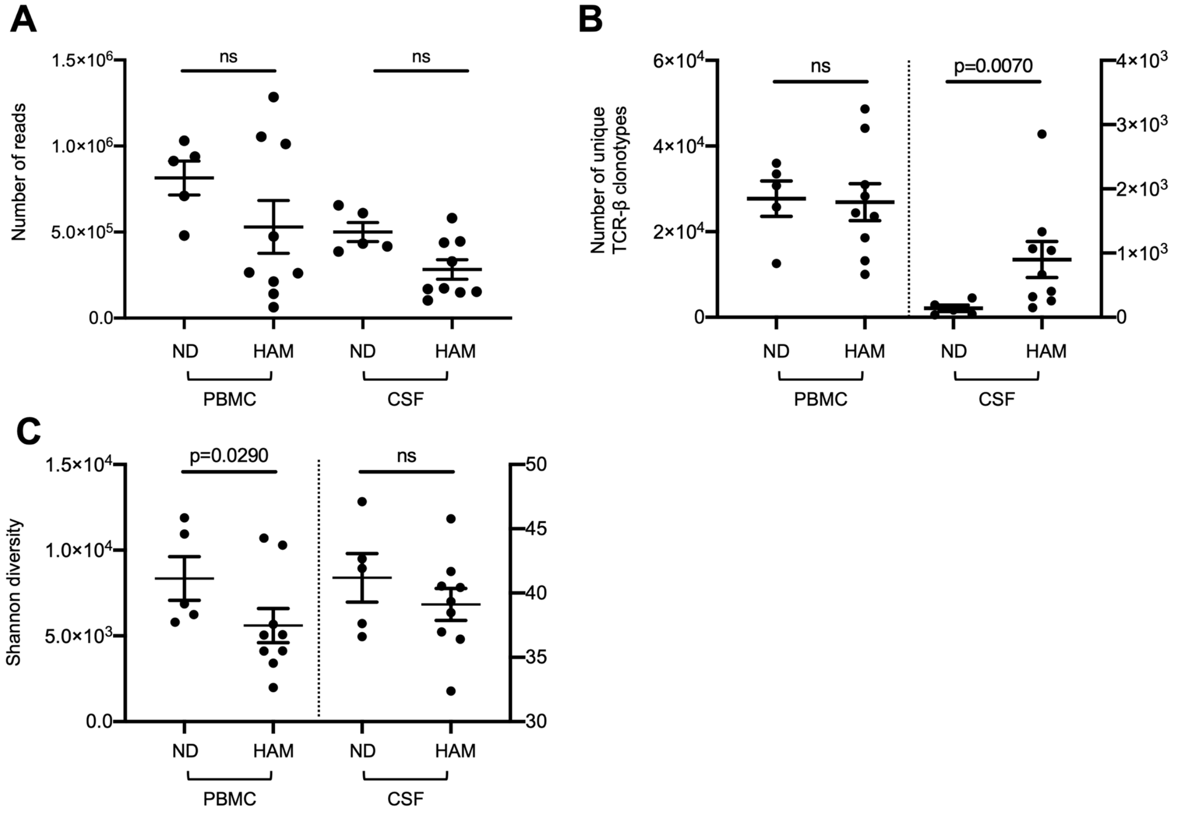

Figure S1. TCR clonal expansion and diversity of TCR-β repertoire in paired PBMC and CSF of HAM/TSP patients and NDs. (**A**) Comparison of the number of total sequence reads between HAM/TSP patients (n=9) and NDs (n=5) in PBMC and CSF using Mann-Whitney test. (**B**) Comparison of the number of unique TCR-β clonotypes between HAM/TSP patients (n=9) and NDs (n=5) in PBMC and CSF using Mann-Whitney test. (**C**) Comparison of Shannon diversity between NDs (n=5) and HAM/TSP patients (n=9) in PBMC (left) and in CSF (right) using Mann-Whitney test. All data represent mean ± SEM.

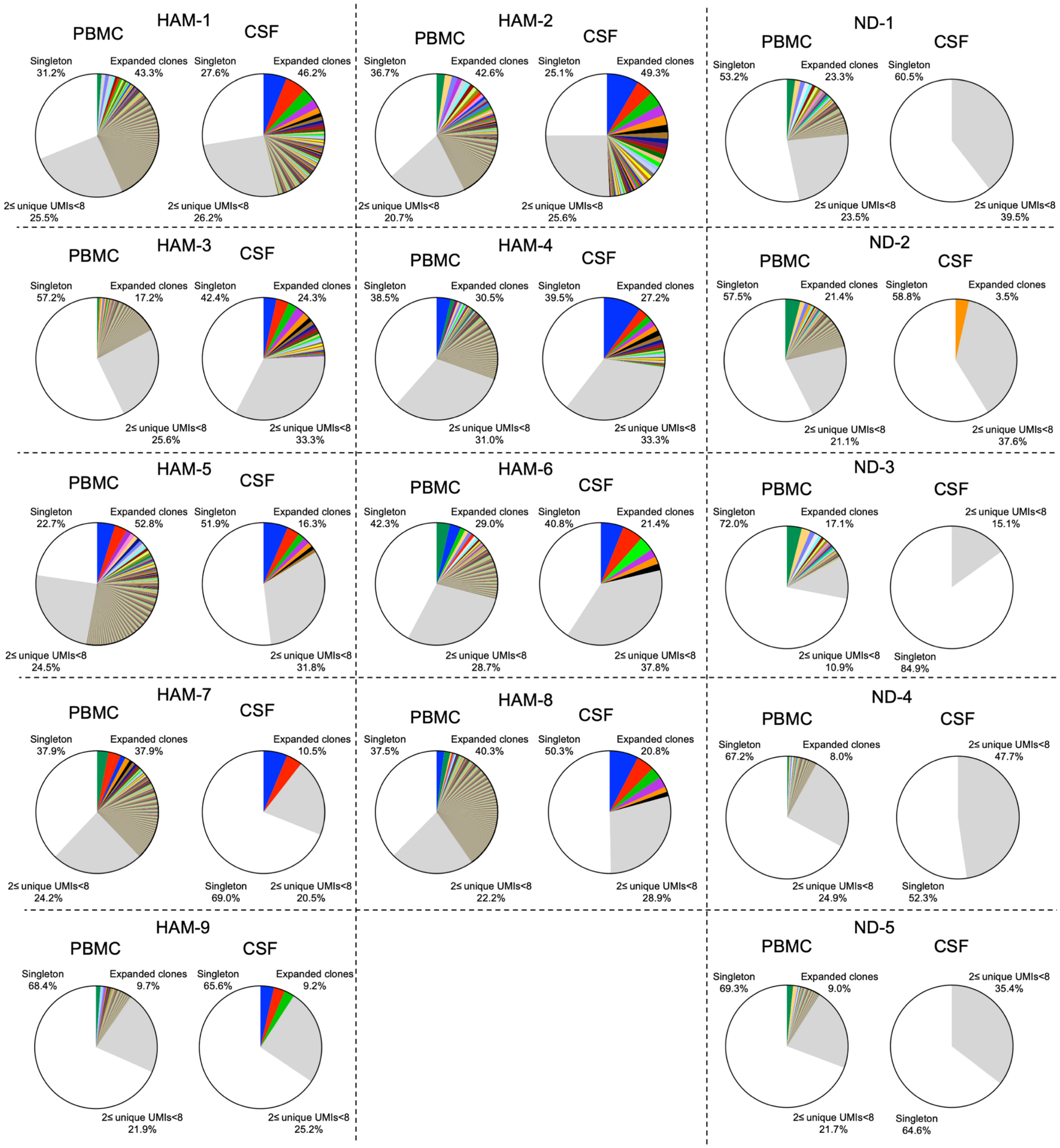

Figure S2. Analysis of T-cell clonal expansion in paired PBMC and CSF of HAM/TSP patients and NDs. TCR clonal expansion was analyzed in paired PBMC and CSF of HAM/TSP patient (n=9) and NDs (n=5) by using the frequencies of clones ≥8 unique UMIs (colored wedges), clones with 2≤unique UMIs<8 (grey color) and singletons (white color). In the group of expanded clones, each wedge represents a unique clonotype with a defined CDR3 sequence and the same clone shared by PBMC and CSF in each individual is visualized in the same color.

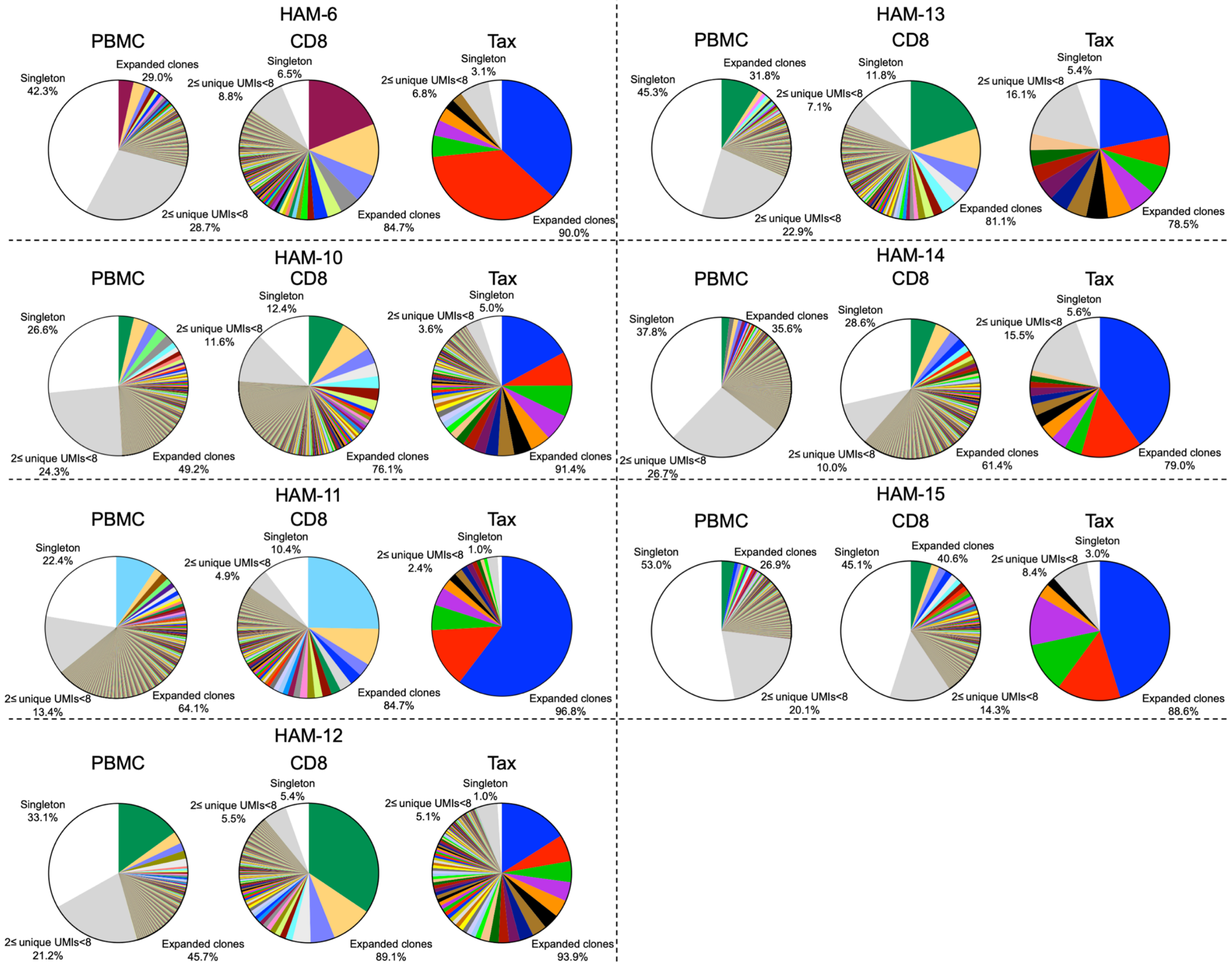

Figure S3. Analysis of T-cell clonal expansion in paired samples of PBMC, enriched CD8⁺ T cells and sorted Tax-specific CD8⁺ T cells of HAM/TSP patients with HLA-A*0201. TCR-β clonal expansion was analyzed in PBMC, enriched CD8⁺ T cells and sorted Tax-specific CD8⁺ T cells of HAM/TSP patient with HLA-A*0201 (n=7) by using the frequency of clones ≥8 unique UMIs (colored wedges), clones with 2≤unique UMIs<8 (grey color) and singletons (white color). In the group of expanded clones, each wedge represents a unique clonotype with a defined CDR3 sequence and the same clone shared among PBMC, CD8⁺ T cells and Tax-specific CD8⁺ T cells is visualized in the same color.

Table S1. TCR-β sequences used for phylogenetic tree cluster analysis of expanded clones in CSF

Seq ID	Read counts	Patient ID	CDR3 sequence	Cluster	Expanded clones groups
seq107	21	HAM1_CSF	CAIRKLDTQYF	1	Shared by CSF and PBMC
seq100	25	HAM1_CSF	CAISGLAGGSDQETQYF	1	Shared by CSF and PBMC
seq133	15	HAM1_CSF	CATRRHTRGGETQYF	1	Unique in CSF
seq173	9	HAM1_CSF	CATSDPGQETQYF	1	Shared by CSF and PBMC
seq75	9	HAM2_CSF	CATSDPGTGRDTQYF	1	Shared by CSF and PBMC
seq90	34	HAM1_CSF	CATSDPTDPTDTQYF	1	Shared by CSF and PBMC
seq224	8	HAM1_CSF	CATSERDRVGETQYF	1	Shared by CSF and PBMC
seq218	8	HAM3_CSF	CATTGTGNTDTQYF	1	Shared by CSF and PBMC
seq82	75	HAM1_CSF	CASSRERGGYTDTQYF	2	Shared by CSF and PBMC
seq145	12	HAM1_CSF	CASTPRGRGTDTQYF	2	Shared by CSF and PBMC
seq220	8	HAM2_CSF	CSAERPSGGATDTQYF	2	Shared by CSF and PBMC
seq44	42	HAM2_CSF	CSAERTSGGATDTQYF	2	Shared by CSF and PBMC
seq89	35	HAM1_CSF	CSAKTSGRGTDTQYF	2	Shared by CSF and PBMC
seq176	9	HAM1_CSF	CSAPDRGRGTDTQYF	2	Shared by CSF and PBMC
seq56	17	HAM2_CSF	CASSGIKGTDTQYF	3	Shared by CSF and PBMC
seq94	29	HAM1_CSF	CASSLEYLAGGTDTQYF	3	Shared by CSF and PBMC
seq38	115	HAM2_CSF	CASSLGAGWGTDTQYF	3	Shared by CSF and PBMC
seq231	8	HAM1_CSF	CASSLVTAIGTDTQYF	3	Clone 1~7 in PBMC
seq18	9	HAM4_CSF	CASSPGPPDTQYF	3	Shared by CSF and PBMC
seq95	28	HAM1_CSF	CASSQRAGDDTQYF	3	Shared by CSF and PBMC
seq11	13	HAM4_CSF	CASSRGRQGTDTQYF	3	Shared by CSF and PBMC
seq119	17	HAM1_CSF	CASSFEQGAADTQYF	4	Unique in CSF
seq34	9	HAM3_CSF	CASSLEPSGGPMDTQYF	4	Shared by CSF and PBMC
seq96	28	HAM1_CSF	CASSLSGGGADTQYF	4	Clone 1~7 in PBMC
seq132	15	HAM1_CSF	CASSLAGETQYF	5	Shared by CSF and PBMC
seq175	9	HAM1_CSF	CASSLANRAETQYF	5	Shared by CSF and PBMC
seq217	8	HAM3_CSF	CASSLGETQYF	5	Clone 1~7 in PBMC
seq159	10	HAM1_CSF	CASSLGWGQAHKYTIYF	5	Unique in CSF
seq23	33	HAM3_CSF	CASSLRTSGGPQETQYF	5	Shared by CSF and PBMC
seq25	19	HAM3_CSF	CASSLRWTGVPETQYF	5	Shared by CSF and PBMC
seq35	9	HAM3_CSF	CASSLTGGLSSGETQYF	5	Shared by CSF and PBMC
seq240	8	HAM1_CSF	CASSPGETQYF	6	Shared by CSF and PBMC
seq126	16	HAM1_CSF	CASSSLTRDQETQYF	6	Shared by CSF and PBMC
seq172	9	HAM1_CSF	CASSSPSKETQYF	6	Shared by CSF and PBMC
seq121	17	HAM1_CSF	CASLNSNTQYF	7	Shared by CSF and PBMC
seq72	9	HAM2_CSF	CASSFGAGGGFGNIQYF	7	Shared by CSF and PBMC
seq195	12	HAM6_CSF	CASSPGLSLAKNIQYF	7	Shared by CSF and PBMC
seq192	34	HAM6_CSF	CASSPHLTVDYGYTF	7	Shared by CSF and PBMC
seq180	9	HAM1_CSF	CASSPWTGLSGNTIYF	7	Shared by CSF and PBMC

seq160	10	HAM1_CSF	CAISDPNREQASYGYTF	8	Clone 1~7 in PBMC
seq30	11	HAM3_CSF	CASSDDRDRGYTF	8	Shared by CSF and PBMC
seq170	9	HAM1_CSF	CASSEQGKYGYTF	8	Shared by CSF and PBMC
seq196	11	HAM6_CSF	CASSLVGSGGYTF	8	Unique in CSF
seq8	21	HAM4_CSF	CASSPGQGGNGYTF	8	Shared by CSF and PBMC
seq198	66	HAM5_CSF	CASSPQGSYGYTF	8	Shared by CSF and PBMC
seq92	31	HAM1_CSF	CASIPDRAGANVLTF	9	Shared by CSF and PBMC
seq166	10	HAM1_CSF	CASSLFRGLLAAGANVLTF	9	Shared by CSF and PBMC
seq234	8	HAM1_CSF	CASSQDALESRDRGSGANVLTF	9	Shared by CSF and PBMC
seq5	37	HAM4_CSF	CASSQEGSRGLFSGANVLTF	9	Shared by CSF and PBMC
seq225	8	HAM1_CSF	CSASTQGGSGANVLTF	9	Clone 1~7 in PBMC
seq73	9	HAM2_CSF	CSVGESGANVLTF	9	Shared by CSF and PBMC
seq48	34	HAM2_CSF	CASGRQGGNQPQHF	10	Shared by CSF and PBMC
seq164	10	HAM1_CSF	CASSIRGYSNQPQHF	10	Shared by CSF and PBMC
seq9	17	HAM4_CSF	CASSLEAGGRNQPQHF	10	Shared by CSF and PBMC
seq37	133	HAM2_CSF	CASSPQGGNQPQHF	10	Shared by CSF and PBMC
seq6	37	HAM4_CSF	CASSQDSGVNQPQHF	10	Unique in CSF
seq116	18	HAM1_CSF	CASSSRGYSNQPQHF	10	Shared by CSF and PBMC
seq78	417	HAM1_CSF	CASSYGAQQPQHF	10	Shared by CSF and PBMC
seq65	11	HAM2_CSF	CATGPQGGNQPQHF	10	Shared by CSF and PBMC
seq60	15	HAM2_CSF	CSAIRTLADQPQHF	10	Unique in CSF
seq215	8	HAM3_CSF	CSASVQNQPQHF	10	Shared by CSF and PBMC
seq111	19	HAM1_CSF	CASRIPGTPLHF	11	Unique in CSF
seq146	12	HAM1_CSF	CASSDLAGSDSPLHF	11	Unique in CSF
seq63	12	HAM2_CSF	CASSLDRAYSPLHF	11	Shared by CSF and PBMC
seq55	18	HAM2_CSF	CASTIGVNNSPLHF	11	Shared by CSF and PBMC
seq112	19	HAM1_CSF	CASRDGQVQYEQYF	12	Shared by CSF and PBMC
seq208	8	HAM4_CSF	CASRDGRGEQYF	12	Clone 1~7 in PBMC
seq62	12	HAM2_CSF	CASRLGLAGGQETQYF	12	Shared by CSF and PBMC
seq190	9	HAM8_CSF	CASRSPAETQYF	12	Shared by CSF and PBMC
seq193	31	HAM6_CSF	CASSQPLAGDYEQYF	12	Shared by CSF and PBMC
seq152	11	HAM1_CSF	CASSSAPPWEQYF	12	Unique in CSF
seq58	16	HAM2_CSF	CASSSPLQGRYEQYF	12	Shared by CSF and PBMC
seq191	12	HAM7_CSF	CAALPDRGSSYEQYF	13	Shared by CSF and PBMC
seq1	253	HAM4_CSF	CASSFEPQRATQYSYEQYF	13	Shared by CSF and PBMC
seq206	8	HAM7_CSF	CASSPDRRPTYEQYF	13	Shared by CSF and PBMC
seq204	9	HAM5_CSF	CASSPDTYEQYF	13	Shared by CSF and PBMC
seq28	14	HAM3_CSF	CASTPREPPYEQYF	13	Shared by CSF and PBMC
seq27	14	HAM3_CSF	CATFNPGTGESYEQYF	13	Shared by CSF and PBMC
seq227	8	HAM1_CSF	CASSFGGGQIEQYF	14	Shared by CSF and PBMC
seq105	23	HAM1_CSF	CASSLGPIYEQYF	14	Shared by CSF and PBMC
seq41	60	HAM2_CSF	CASSLGQGGEQYF	14	Shared by CSF and PBMC
seq154	11	HAM1_CSF	CASSLGSHLYEQYF	14	Shared by CSF and PBMC
seq186	50	HAM8_CSF	CASSLIAGAEQYF	14	Shared by CSF and PBMC

seq153	11	HAM1_CSF	CASSLQDGYEQYF	14	Clone 1~7 in PBMC
seq228	8	HAM1_CSF	CASSLQTRGIQYF	14	Shared by CSF and PBMC
seq21	47	HAM3_CSF	CASSLTTPGLSYEQYF	14	Shared by CSF and PBMC
seq85	60	HAM1_CSF	CASSLYQGYEQYF	14	Shared by CSF and PBMC
seq26	15	HAM3_CSF	CSALVGSAEAFF	15	Shared by CSF and PBMC
seq236	8	HAM1_CSF	CSAPTSRGTEAFF	15	Shared by CSF and PBMC
seq40	77	HAM2_CSF	CSARAGTGIVLGNTEAFF	15	Unique in CSF
seq64	11	HAM2_CSF	CSARGWDEAFF	15	Shared by CSF and PBMC
seq123	16	HAM1_CSF	CSVEEGLGEAFF	15	Shared by CSF and PBMC
seq114	18	HAM1_CSF	CSVEMAGWSGTEAFF	15	Shared by CSF and PBMC
seq179	9	HAM1_CSF	CSAKRARERNSPLHF	16	Unique in CSF
seq223	8	HAM2_CSF	CSARDFVGQPQHF	16	Shared by CSF and PBMC
seq205	8	HAM8_CSF	CSARDLADLYNSPLHF	16	Shared by CSF and PBMC
seq129	15	HAM1_CSF	CSARDRTGWTNEKLFF	16	Unique in CSF
seq120	17	HAM1_CSF	CSARDVTGLQPQHF	16	Shared by CSF and PBMC
seq67	10	HAM2_CSF	CSAREGPQPQHF	16	Shared by CSF and PBMC
seq161	10	HAM1_CSF	CSAFLGTSGYTF	17	Shared by CSF and PBMC
seq212	8	HAM3_CSF	CSARDRETYGYTF	17	Shared by CSF and PBMC
seq24	21	HAM3_CSF	CSARDRFTYGYTF	17	Shared by CSF and PBMC
seq109	20	HAM1_CSF	CSARGSRDVRSGYTF	17	Shared by CSF and PBMC
seq147	12	HAM1_CSF	CSATRGTYGYTF	17	Clone 1~7 in PBMC
seq83	73	HAM1_CSF	CSALTGSGELFF	18	Shared by CSF and PBMC
seq51	29	HAM2_CSF	CSAREGADFF	18	Shared by CSF and PBMC
seq39	86	HAM2_CSF	CSARTGQDFF	18	Shared by CSF and PBMC
seq168	10	HAM1_CSF	CSALPGTSGRKTYNEQFF	19	Shared by CSF and PBMC
seq59	15	HAM2_CSF	CSARGQGYEQFF	19	Unique in CSF
seq36	253	HAM2_CSF	CSARPTLGRGLNEQFF	19	Shared by CSF and PBMC
seq104	23	HAM1_CSF	CSARTGTWGPYNEQFF	19	Clone 1~7 in PBMC
seq57	16	HAM2_CSF	CSAWGSGVSNEQFF	19	Shared by CSF and PBMC
seq87	49	HAM1_CSF	CSAAPGTGVEQYF	20	Shared by CSF and PBMC
seq141	13	HAM1_CSF	CSAGVPFVVPNIQYF	20	Shared by CSF and PBMC
seq110	20	HAM1_CSF	CSVEVGQGYEQYF	20	Shared by CSF and PBMC
seq136	14	HAM1_CSF	CSAGDNRYEQYF	21	Shared by CSF and PBMC
seq61	12	HAM2_CSF	CSARDSATEQYF	21	Shared by CSF and PBMC
seq81	113	HAM1_CSF	CSARDSGVGYEQYF	21	Shared by CSF and PBMC
seq76	9	HAM2_CSF	CSARENAYEQYF	21	Shared by CSF and PBMC
seq167	10	HAM1_CSF	CSARGTGTSGYEQYF	21	Unique in CSF
seq219	8	HAM2_CSF	CSARVGVEQYF	21	Shared by CSF and PBMC
seq155	11	HAM1_CSF	CSARTSGGTQYF	22	Clone 1~7 in PBMC
seq200	18	HAM5_CSF	CSARTSGQAREQYF	22	Shared by CSF and PBMC
seq98	27	HAM1_CSF	CSARTSGQGREQYF	22	Shared by CSF and PBMC
seq178	9	HAM1_CSF	CSALSRSSGNTIYF	23	Shared by CSF and PBMC
seq77	464	HAM1_CSF	CSARDEDRGGNTIYF	23	Shared by CSF and PBMC
seq184	10	HAM9_CSF	CSARDLGAQETQYF	23	Shared by CSF and PBMC

seq183	12	HAM9_CSF	CSARDLGQQETQYF	23	Clone 1~7 in PBMC
seq203	10	HAM5_CSF	CSARDPGRGGQETQYF	23	Shared by CSF and PBMC
seq185	9	HAM9_CSF	CSARPLLAQETQYF	23	Clone 1~7 in PBMC
seq137	14	HAM1_CSF	CSAEQSGTDTQYF	24	Shared by CSF and PBMC
seq181	9	HAM1_CSF	CSAPPRERGTDTQYF	24	Shared by CSF and PBMC
seq102	25	HAM1_CSF	CSARDLNRGQDTQYF	24	Shared by CSF and PBMC
seq148	12	HAM1_CSF	CSARRPSGRDTQYF	24	Shared by CSF and PBMC
seq144	13	HAM1_CSF	CSHRTGFGTDTQYF	24	Shared by CSF and PBMC
seq233	8	HAM1_CSF	CSVAASGGADTQYF	24	Clone 1~7 in PBMC
seq47	36	HAM2_CSF	CSVPAPDGNTDTQYF	24	Shared by CSF and PBMC
seq211	8	HAM4_CSF	CASSLALVAEAGIRNEQFF	25	Clone 1~7 in PBMC
seq74	9	HAM2_CSF	CASSSRLANNEQFF	25	Shared by CSF and PBMC
seq10	16	HAM4_CSF	CASSVALGQGSHNEQFF	25	Unique in CSF
seq13	12	HAM4_CSF	CASGQGSNEQFF	26	Shared by CSF and PBMC
seq103	24	HAM1_CSF	CASSPINSGTGVYNEQFF	26	Shared by CSF and PBMC
seq143	13	HAM1_CSF	CASSQGGVSGRLNEQFF	26	Shared by CSF and PBMC
seq209	8	HAM4_CSF	CASSQGSGPLNEQFF	26	Clone 1~7 in PBMC
seq142	13	HAM1_CSF	CASSQQRGGWADEQFF	26	Unique in CSF
seq169	9	HAM1_CSF	CASSYGLSNEQFF	26	Shared by CSF and PBMC
seq43	43	HAM2_CSF	CASNLDPLAGGYNEQFF	27	Shared by CSF and PBMC
seq213	8	HAM3_CSF	CASRVDKSSYNEQFF	27	Shared by CSF and PBMC
seq128	15	HAM1_CSF	CASSFQRTSGYVLSYNEQFF	27	Clone 1~7 in PBMC
seq127	16	HAM1_CSF	CASSLRDTYNEQFF	27	Shared by CSF and PBMC
seq19	66	HAM3_CSF	CASSYSPDWGINEQFF	27	Clone 1~7 in PBMC
seq214	8	HAM3_CSF	CASSARQGPSRNEQFF	28	Shared by CSF and PBMC
seq79	263	HAM1_CSF	CASSPSRSEQFF	28	Shared by CSF and PBMC
seq130	15	HAM1_CSF	CASSPYGTGNEQFF	28	Shared by CSF and PBMC
seq239	8	HAM1_CSF	CASSRSGSEQFF	28	Shared by CSF and PBMC
seq125	16	HAM1_CSF	CAIRQDGGNEKLFF	29	Shared by CSF and PBMC
seq139	13	HAM1_CSF	CASRETEMLNTEAFF	29	Shared by CSF and PBMC
seq86	58	HAM1_CSF	CASSLGGEAFF	29	Shared by CSF and PBMC
seq122	17	HAM1_CSF	CASSREVLAGPGELFF	29	Shared by CSF and PBMC
seq118	17	HAM1_CSF	CASSSTGTTGELFF	29	Clone 1~7 in PBMC
seq151	11	HAM1_CSF	CASSSTILGGLAFF	29	Clone 1~7 in PBMC
seq115	18	HAM1_CSF	CATEGSITEEKLFF	29	Clone 1~7 in PBMC
seq162	10	HAM1_CSF	CASRATANTGELFF	30	Shared by CSF and PBMC
seq101	25	HAM1_CSF	CSALAGTGELFF	30	Shared by CSF and PBMC
seq201	17	HAM5_CSF	CSALVGFQSTGELFF	30	Shared by CSF and PBMC
seq135	15	HAM1_CSF	CSARDAGVAGSRTGELFF	30	Shared by CSF and PBMC
seq68	10	HAM2_CSF	CSARGNTGELFF	30	Unique in CSF
seq140	13	HAM1_CSF	CSARMASGRKRTGELFF	30	Unique in CSF
seq238	8	HAM1_CSF	CSASGLNRDTGELFF	30	Unique in CSF
seq157	10	HAM1_CSF	CSASRADTGELFF	30	Shared by CSF and PBMC
seq210	8	HAM4_CSF	CASSFGGGNTGELFF	31	Unique in CSF

seq3	42	HAM4_CSF	CASSKRQNTGELFF	31	Shared by CSF and PBMC
seq84	60	HAM1_CSF	CASSPRTSGDWNTGELFF	31	Unique in CSF
seq80	178	HAM1_CSF	CASSLDPGGAGELFF	32	Shared by CSF and PBMC
seq50	32	HAM2_CSF	CASSLDRLLPLLNTGELFF	32	Shared by CSF and PBMC
seq12	13	HAM4_CSF	CASSLDTRSTGELFF	32	Shared by CSF and PBMC
seq124	16	HAM1_CSF	CASSLFTGELFF	32	Clone 1~7 in PBMC
seq230	8	HAM1_CSF	CASSPRAGANTEAFF	33	Shared by CSF and PBMC
seq93	30	HAM1_CSF	CASSPRPNAEAFF	33	Shared by CSF and PBMC
seq31	11	HAM3_CSF	CASSPRTNTEAFF	33	Shared by CSF and PBMC
seq99	25	HAM1_CSF	CASGRAYMNTEAFF	34	Unique in CSF
seq14	12	HAM4_CSF	CASSSGTVNTEAFF	34	Clone 1~7 in PBMC
seq32	11	HAM3_CSF	CASSSQENTEAFF	34	Shared by CSF and PBMC
seq199	34	HAM5_CSF	CASSSRGPAMNTEAFF	34	Shared by CSF and PBMC
seq158	10	HAM1_CSF	CASSSRVNTEAFF	34	Shared by CSF and PBMC
seq221	8	HAM2_CSF	CASSVYRGGNTEAFF	34	Shared by CSF and PBMC
seq29	12	HAM3_CSF	CASSFGANEKLFF	35	Shared by CSF and PBMC
seq88	47	HAM1_CSF	CASSLEGRRGREKLFF	35	Shared by CSF and PBMC
seq49	32	HAM2_CSF	CASSLSGWQGYEKLFF	35	Shared by CSF and PBMC
seq187	31	HAM8_CSF	CASSPRAGPVEKLFF	35	Shared by CSF and PBMC
seq226	8	HAM1_CSF	CASSQVGATNEKLFF	35	Shared by CSF and PBMC
seq163	10	HAM1_CSF	CACGGSMNTEAFF	36	Shared by CSF and PBMC
seq138	14	HAM1_CSF	CAIRTRDNTEAFF	36	Clone 1~7 in PBMC
seq117	18	HAM1_CSF	CASSAASETEAFF	36	Shared by CSF and PBMC
seq17	10	HAM4_CSF	CASSAGTGGHRDTEAFF	36	Shared by CSF and PBMC
seq229	8	HAM1_CSF	CASSLDRWAGRAITEAFF	36	Unique in CSF
seq131	15	HAM1_CSF	CASSLMDRGTEAFF	36	Shared by CSF and PBMC
seq171	9	HAM1_CSF	CASSPGRGPHTEAFF	37	Shared by CSF and PBMC
seq134	15	HAM1_CSF	CASSPGVQGLYTEAFF	37	Clone 1~7 in PBMC
seq33	10	HAM3_CSF	CASSYGSEAFF	37	Shared by CSF and PBMC
seq20	64	HAM3_CSF	CASSYNGEAFF	37	Shared by CSF and PBMC
seq53	24	HAM2_CSF	CASSAGLAGGPRQFF	38	Shared by CSF and PBMC
seq91	33	HAM1_CSF	CASSFLTAGGPGEQFF	38	Clone 1~7 in PBMC
seq66	11	HAM2_CSF	CASSPGLAGGVEQFF	38	Shared by CSF and PBMC
seq4	38	HAM4_CSF	CASSPLEGGNNEQFF	38	Shared by CSF and PBMC
seq197	10	HAM6_CSF	CASSPLGGYEQFF	38	Unique in CSF
seq189	18	HAM8_CSF	CASSLDGSEQFF	39	Shared by CSF and PBMC
seq52	25	HAM2_CSF	CASSLGLASASWEQYF	39	Shared by CSF and PBMC
seq188	20	HAM8_CSF	CASSLTGTEQFF	39	Shared by CSF and PBMC
seq113	19	HAM1_CSF	CARAIGTGGHEQFF	NA	Shared by CSF and PBMC
seq54	24	HAM2_CSF	CASSEAGGYYEQYF	NA	Shared by CSF and PBMC
seq232	8	HAM1_CSF	CASSEGGGYGYTF	NA	Clone 1~7 in PBMC
seq97	27	HAM1_CSF	CASSEGGHTRQDTQYF	NA	Shared by CSF and PBMC
seq46	39	HAM2_CSF	CASSFPGQGNPEQFF	NA	Shared by CSF and PBMC
seq150	11	HAM1_CSF	CASSFWGAPSQPQHF	NA	Shared by CSF and PBMC

seq2	66	HAM4_CSF	CASSHGSQPQHF	NA	Unique in CSF
seq71	9	HAM2_CSF	CASSHPGTGRTEAFF	NA	Shared by CSF and PBMC
seq70	9	HAM2_CSF	CASSHQGTSGLTDTQYF	NA	Clone 1~7 in PBMC
seq106	22	HAM1_CSF	CASSHWDRAYEQYF	NA	Shared by CSF and PBMC
seq207	8	HAM4_CSF	CASSKGTPGEQYF	NA	Unique in CSF
seq16	11	HAM4_CSF	CASSLAFGSNTEAFF	NA	Shared by CSF and PBMC
seq22	46	HAM3_CSF	CASSLERGNEQYF	NA	Shared by CSF and PBMC
seq15	11	HAM4_CSF	CASSLGGRNTEAFF	NA	Unique in CSF
seq202	12	HAM5_CSF	CASSLGQGGTEAFF	NA	Shared by CSF and PBMC
seq237	8	HAM1_CSF	CASSLQGGLSDTQYF	NA	Unique in CSF
seq182	9	HAM1_CSF	CASSLVRHEQFF	NA	Shared by CSF and PBMC
seq222	8	HAM2_CSF	CASSNPGQGANEQYF	NA	Shared by CSF and PBMC
seq165	10	HAM1_CSF	CASSQAGTQYF	NA	Clone 1~7 in PBMC
seq177	9	HAM1_CSF	CASSQHPRNTEAFF	NA	Shared by CSF and PBMC
seq216	8	HAM3_CSF	CASSRRTGGTDTQYF	NA	Shared by CSF and PBMC
seq7	28	HAM4_CSF	CASSSGRGLNEQFF	NA	Clone 1~7 in PBMC
seq235	8	HAM1_CSF	CASSTDRGTQYF	NA	Shared by CSF and PBMC
seq174	9	HAM1_CSF	CASSTVQGHYYGYTF	NA	Unique in CSF
seq42	50	HAM2_CSF	CASSVGQGAEQYF	NA	Shared by CSF and PBMC
seq194	22	HAM6_CSF	CASSYTGEVQYF	NA	Shared by CSF and PBMC
seq149	12	HAM1_CSF	CATSDRTSETDEQYF	NA	Shared by CSF and PBMC
seq108	20	HAM1_CSF	CSASISGRVNNEQFF	NA	Shared by CSF and PBMC
seq45	41	HAM2_CSF	CSASPSQGATDTQYF	NA	Shared by CSF and PBMC
seq156	11	HAM1_CSF	CSASQPARVTDEQFF	NA	Clone 1~7 in PBMC
seq69	10	HAM2_CSF	CSASRALGLRETQYF	NA	Clone 1~7 in PBMC

CDR3, complementary determining region 3

Table S2. TCR-β clonotypes of expanded clones shared by three populations

Patient	CDR3 sequence	PBMCs			Enriched CD8$^+$ T cells			Tax-specific CD8$^+$ T cells		
		Rank	Count	%	Rank	Count	%	Rank	Count	%
HAM-6	CASSQPLAGDYEQYF	11	29	0.37%	6	671	3.15%	1	199	36.78%
HAM-6	CASSQDPHLQGARTEAFF	61	7	0.09%	NA	NA	NA	2	198	36.60%
HAM-6	CASSPGLSLAKNIQYF	218	3	0.04%	31	63	0.30%	3	27	4.99%
HAM-6	CSAKASEQFF	35	9	0.11%	17	142	0.67%	4	20	3.70%
HAM-6	CASSQPHLAGGNEQFF	4276	1	0.01%	56	40	0.19%	5	17	3.14%
HAM-6	CASSDPGQGNYEQYF	50	8	0.10%	16	142	0.67%	6	13	2.40%
HAM-6	CASSYPGQGVWTQYF	119	4	0.05%	82	25	0.12%	7	13	2.40%
HAM-6	CASSYPGQGYNEQFF	259	3	0.04%	75	29	0.14%	8	4	0.74%
HAM-6	CASSSPGTGAYEQYF	NA	NA	NA	148	14	0.07%	9	3	0.55%
HAM-6	CATSPGLAGGNSGANVLTF	173	4	0.05%	24	86	0.40%	10	3	0.55%
HAM-6	CSAAGAIQYF	400	2	0.03%	94	21	0.10%	11	3	0.55%
HAM-6	CASTDGLAGATGELFF	NA	NA	NA	1149	1	0.00%	12	3	0.55%
HAM-6	CASSSPGQGQETQYF	NA	NA	NA	175	12	0.06%	13	3	0.55%
HAM-6	CASSLDQLVGDYEQYF	NA	NA	NA	341	5	0.02%	14	2	0.37%
HAM-6	CSARDPGTGLNTEAFF	NA	NA	NA	322	5	0.02%	15	2	0.37%
HAM-6	CASSSPGTGQVEQYF	NA	NA	NA	NA	NA	NA	16	2	0.37%
HAM-6	CASSQEVLTWGTGELFF	2764	1	0.01%	157	14	0.07%	17	2	0.37%
HAM-6	CASSQVLSGGLQETQYF	NA	NA	NA	NA	NA	NA	18	2	0.37%
HAM-6	CASSQDPASMNTEAFF	NA	NA	NA	278	6	0.03%	19	2	0.37%
HAM-6	CSARYGAEAFF	NA	NA	NA	NA	NA	NA	20	2	0.37%
HAM-6	CASRPGLAGAQDTQYF	2061	1	0.01%	729	2	0.01%	21	2	0.37%
HAM-10	CASSPGQGVMDTQYF	14	251	0.45%	8	1033	0.95%	1	5331	17.07%
HAM-10	CASTPGLKGAADTQYF	13	271	0.49%	9	873	0.80%	2	2452	7.85%
HAM-10	CASSQRPEYMNTEAFF	47	87	0.16%	22	487	0.45%	3	2263	7.25%
HAM-10	CASSLRSPKGETQYF	22	176	0.32%	13	738	0.68%	4	1929	6.18%
HAM-10	CASSFGLAGSFNNEQFF	50	83	0.15%	35	341	0.31%	5	1475	4.72%
HAM-10	CASSPGLAGAPGDTQYF	41	97	0.17%	23	466	0.43%	6	1272	4.07%
HAM-10	CASSQEQGVNTEAFF	93	44	0.08%	51	222	0.20%	7	1176	3.77%
HAM-10	CASSSGLAGGIYEQYF	59	61	0.11%	45	261	0.24%	8	918	2.94%
HAM-10	CASSPGLAGGETQYF	114	34	0.06%	66	172	0.16%	9	857	2.74%
HAM-10	CASSSSPWPSTDTQYF	55	71	0.13%	62	185	0.17%	10	853	2.73%
HAM-10	CASSPGLAGRTGELFF	110	35	0.06%	61	187	0.17%	11	633	2.03%
HAM-10	CASSFGLAGGQGDTQYF	71	52	0.09%	58	203	0.19%	12	543	1.74%
HAM-10	CASRPGLADGTDTQYF	160	24	0.04%	92	118	0.11%	13	480	1.54%
HAM-10	CASSHGQGLTDTQYF	101	39	0.07%	76	146	0.13%	14	467	1.50%
HAM-10	CASRPGLAGALDTQYF	144	27	0.05%	90	120	0.11%	15	428	1.37%
HAM-10	CSARDVGLNYGYTF	417	11	0.02%	185	54	0.05%	16	421	1.35%
HAM-10	CASSLGLAGGIVEQFF	257	16	0.03%	173	57	0.05%	17	325	1.04%
HAM-10	CASSPAATDMNTEAFF	760	6	0.01%	194	50	0.05%	18	293	0.94%
HAM-10	CASSPGPGQGHQPQHF	429	11	0.02%	148	67	0.06%	19	284	0.91%
HAM-10	CSARDRAGKETQYF	374	12	0.02%	224	43	0.04%	20	283	0.91%
HAM-10	CSARSGQDVF	419	11	0.02%	245	38	0.03%	21	221	0.71%
HAM-10	CASSQPQGLHTEAFF	514	9	0.02%	196	50	0.05%	22	219	0.70%

HAM-10	CASTQGLAGVNEQFF	291	14	0.03%	157	62	0.06%	23	173	0.55%
HAM-10	CASSITSGRAPEQFF	NA	NA	NA	351	26	0.02%	24	164	0.53%
HAM-10	CASRMGLAGGPETQYF	1048	5	0.01%	297	30	0.03%	25	153	0.49%
HAM-10	CASSATGTLIPEAFF	725	7	0.01%	237	40	0.04%	26	145	0.46%
HAM-10	CASSQVLQRGASGEQYF	1756	3	0.01%	293	30	0.03%	27	135	0.43%
HAM-10	CASRQGLDGGTGELFF	1140	5	0.01%	231	42	0.04%	28	134	0.43%
HAM-10	CASSPGQGITDTQYF	401	11	0.02%	358	25	0.02%	29	127	0.41%
HAM-10	CASSPGLAGSNEQFF	NA	NA	NA	393	23	0.02%	30	127	0.41%
HAM-10	CASTPGLAGGRSTDTQYF	355	12	0.02%	198	49	0.04%	31	126	0.40%
HAM-10	CASSLDPLESGMNTEAFF	NA	NA	NA	277	32	0.03%	32	119	0.38%
HAM-10	CASSPLGRGDYEQYF	473	10	0.02%	310	29	0.03%	33	114	0.36%
HAM-10	CASSSPRTGVYGYTF	1446	4	0.01%	236	40	0.04%	34	109	0.35%
HAM-10	CASSPGQGVMDAQYF	804	6	0.01%	376	24	0.02%	35	107	0.34%
HAM-10	CASSSPREGNYGYTF	1301	4	0.01%	333	27	0.02%	36	100	0.32%
HAM-10	CASSSPGTGRETQYF	392	11	0.02%	318	28	0.03%	37	99	0.32%
HAM-10	CASSLGLAGGPGELFF	NA	NA	NA	549	16	0.01%	38	98	0.31%
HAM-10	CASSGTGYSPLHF	1323	4	0.01%	580	15	0.01%	39	94	0.30%
HAM-10	CASSFEQGSMNTEAFF	888	6	0.01%	528	17	0.02%	40	92	0.29%
HAM-10	CASSIGQGSDEQFF	1479	4	0.01%	636	14	0.01%	41	92	0.29%
HAM-10	CASSGIGDRSYEQYF	628	8	0.01%	252	37	0.03%	42	90	0.29%
HAM-10	CASNPGLAGVAGELFF	856	6	0.01%	1227	7	0.01%	43	89	0.28%
HAM-10	CASSHPGTGVNEQFF	1342	4	0.01%	336	27	0.02%	44	76	0.24%
HAM-10	CASSPPTAEMNTEAFF	2045	3	0.01%	849	10	0.01%	45	74	0.24%
HAM-10	CASSQGQGITDTQYF	4378	2	0.00%	457	19	0.02%	46	74	0.24%
HAM-10	CASRSGLAGDTGELFF	4292	2	0.00%	629	14	0.01%	47	73	0.23%
HAM-10	CASSQEQGRSEQFF	2308	3	0.01%	520	17	0.02%	48	71	0.23%
HAM-10	CASSPGLAALRSYNEQFF	2566	3	0.01%	350	26	0.02%	49	70	0.22%
HAM-10	CASSYSSGQAHQPQHF	2787	2	0.00%	826	10	0.01%	50	68	0.22%
HAM-10	CASSLAGGRASAGGPLGTQYF	NA	NA	NA	804	11	0.01%	51	68	0.22%
HAM-10	CASSRGQGMYNEQFF	NA	NA	NA	517	17	0.02%	52	64	0.20%
HAM-10	CASSPGLAGGRGNEQFF	1155	5	0.01%	1098	8	0.01%	53	63	0.20%
HAM-10	CASSHGLAGDYEQYF	769	6	0.01%	501	17	0.02%	54	62	0.20%
HAM-10	CASSFPFQGIYGYTF	NA	NA	NA	621	14	0.01%	55	60	0.19%
HAM-10	CASSQDERVMVNTEAFF	1984	3	0.01%	842	10	0.01%	56	55	0.18%
HAM-10	CASSPGLRGGLEQFF	1185	4	0.01%	563	15	0.01%	57	53	0.17%
HAM-10	CASIPGLAGGYNEQFF	699	7	0.01%	346	26	0.02%	58	52	0.17%
HAM-10	CASSEPLAGAYEQYF	NA	NA	NA	687	13	0.01%	59	45	0.14%
HAM-10	CASSQDPLAGHYEQYF	2546	3	0.01%	757	12	0.01%	60	42	0.13%
HAM-10	CASSQDFRLAGAYNEQFF	NA	NA	NA	2918	3	0.00%	61	39	0.12%
HAM-10	CASSLGLEQYF	818	6	0.01%	249	37	0.03%	62	38	0.12%
HAM-10	CASTSGLAGTDTQYF	612	8	0.01%	273	33	0.03%	63	38	0.12%
HAM-10	CASSPGLAGRQETQYF	1993	3	0.01%	1023	8	0.01%	64	38	0.12%
HAM-10	CASSQPLAGYNEQFF	3763	2	0.00%	NA	NA	NA	65	38	0.12%
HAM-10	CASSQDWMASGAYNEQFF	17553	1	0.00%	1248	7	0.01%	66	37	0.12%
HAM-10	CASSDPGQGIFEQFF	1780	3	0.01%	1900	4	0.00%	67	34	0.11%
HAM-10	CASSQDFSIRNQETQYF	5232	2	0.00%	886	10	0.01%	68	33	0.11%
HAM-10	CAWSDPLVGSTEAFF	NA	NA	NA	NA	NA	NA	69	32	0.10%

HAM-10	CASREGLAGGNRPGELFF	9866	1	0.00%	1362	6	0.01%	70	32	0.10%
HAM-10	CASRPGLAGGPGELFF	947	5	0.01%	535	16	0.01%	71	31	0.10%
HAM-10	CASSHPLAGANEQFF	NA	NA	NA	2179	4	0.00%	72	30	0.10%
HAM-10	CASSPLGGYEQFF	1	2012	3.60%	2	8522	7.80%	73	30	0.10%
HAM-10	CASSFGLAGVTGELFF	NA	NA	NA	NA	NA	NA	74	29	0.09%
HAM-10	CASSSWGLNEKLFF	6071	1	0.00%	1854	4	0.00%	75	29	0.09%
HAM-10	CSANLNEQFF	610	8	0.01%	140	70	0.06%	76	29	0.09%
HAM-10	CASTPGLAGGPPEQFF	NA	NA	NA	1101	8	0.01%	77	27	0.09%
HAM-10	CASTPGLKGAADAQYF	1017	5	0.01%	781	11	0.01%	78	27	0.09%
HAM-10	CASSLGTPEQFF	NA	NA	NA	534	16	0.01%	79	26	0.08%
HAM-10	CASSPPQGRTEAFF	NA	NA	NA	NA	NA	NA	80	26	0.08%
HAM-10	CASSSAGANPYEQYF	2369	3	0.01%	1747	5	0.00%	81	26	0.08%
HAM-10	CASSSPRTGTHSGANVLTF	4517	2	0.00%	1210	7	0.01%	82	25	0.08%
HAM-10	CASSQVLTGGGNEKLFF	9457	1	0.00%	1962	4	0.00%	83	24	0.08%
HAM-10	CASSQDPARMNTEAFF	10439	1	0.00%	2574	3	0.00%	84	24	0.08%
HAM-10	CASSLGLSGGYNEQFF	NA	NA	NA	1488	6	0.01%	85	24	0.08%
HAM-10	CASSYPQAGTYEQYF	3570	2	0.00%	1603	5	0.00%	86	23	0.07%
HAM-10	CASSEPLTGYYEQYF	19889	1	0.00%	1496	6	0.01%	87	23	0.07%
HAM-10	CASSPGLAGSSGELFF	19334	1	0.00%	NA	NA	NA	88	23	0.07%
HAM-10	CASSFPGRDRGANEQFF	16255	1	0.00%	2907	3	0.00%	89	22	0.07%
HAM-10	CASRTGLAGVGVREFF	1743	3	0.01%	1121	7	0.01%	90	21	0.07%
HAM-10	CASSDGLAGVYEQFF	NA	NA	NA	12120	1	0.00%	91	20	0.06%
HAM-10	CASSQGQGKSPLHF	NA	NA	NA	NA	NA	NA	92	19	0.06%
HAM-10	CASSPGLAGGGWETQYF	4313	2	0.00%	3945	2	0.00%	93	18	0.06%
HAM-10	CASSQDHREMNTEAFF	4893	2	0.00%	NA	NA	NA	94	18	0.06%
HAM-10	CAISDLGKGSYNEQFF	18318	1	0.00%	3032	3	0.00%	95	18	0.06%
HAM-10	CSARFGAPLHF	8705	1	0.00%	2480	3	0.00%	96	17	0.05%
HAM-10	CASSQDVASGGAPDTQYF	4524	2	0.00%	NA	NA	NA	97	17	0.05%
HAM-10	CASSHGQGLTDAQYF	12057	1	0.00%	2673	3	0.00%	98	16	0.05%
HAM-10	CASSPPQGLAQEQYF	NA	NA	NA	NA	NA	NA	99	16	0.05%
HAM-10	CASSLRSPKGEAQYF	NA	NA	NA	1063	8	0.01%	100	16	0.05%
HAM-10	CASRGGLAGGLGYNEQFF	4342	2	0.00%	13452	1	0.00%	101	16	0.05%
HAM-10	CASSLVGSGGYTF	2	1992	3.57%	1	8971	8.21%	102	15	0.05%
HAM-10	CASSLPPTDMNTEAFF	9494	1	0.00%	1581	5	0.00%	103	15	0.05%
HAM-10	CAGQPTTNTGELFF	NA	NA	NA	NA	NA	NA	104	15	0.05%
HAM-10	CASSPGQGTVAEAFF	NA	NA	NA	NA	NA	NA	105	15	0.05%
HAM-10	CASSLGLAGGTDTQYF	17917	1	0.00%	3007	3	0.00%	106	14	0.04%
HAM-10	CASSPGQGVRDTQYF	NA	NA	NA	1165	7	0.01%	107	14	0.04%
HAM-10	CASSESERGDYGYTF	NA	NA	NA	1238	7	0.01%	108	14	0.04%
HAM-10	CASSDPGQGSLYGYTF	12946	1	0.00%	2732	3	0.00%	109	13	0.04%
HAM-10	CASRGGLTANTEAFF	16794	1	0.00%	15449	1	0.00%	110	13	0.04%
HAM-10	CASSDPLQGVGYGYTF	NA	NA	NA	1492	6	0.01%	111	13	0.04%
HAM-10	CASSQPQGLNTEAFF	8501	1	0.00%	NA	NA	NA	112	13	0.04%
HAM-10	CASSYTTGRVNTEAFF	1800	3	0.01%	903	9	0.01%	113	13	0.04%
HAM-10	CASIAPRQGIYEQYF	NA	NA	NA	4296	2	0.00%	114	13	0.04%
HAM-10	CASSQQQGHTEAFF	NA	NA	NA	NA	NA	NA	115	13	0.04%
HAM-10	CATSTAGGSYEQYF	6920	1	0.00%	NA	NA	NA	116	13	0.04%

HAM-10	CASRAGLAGVGEQYF	NA	NA	NA	NA	NA	NA	117	13	0.04%
HAM-10	CASSLPLAGVYEQYF	5438	2	0.00%	NA	NA	NA	118	13	0.04%
HAM-10	CASSPGLAGGLGTGELFF	2415	3	0.01%	2964	3	0.00%	119	13	0.04%
HAM-10	CASSSYLGGNVEAFF	NA	NA	NA	1693	5	0.00%	120	13	0.04%
HAM-10	CASSPDPLSGAHHEQFF	9213	1	0.00%	2517	3	0.00%	121	13	0.04%
HAM-10	CASSHGLAGSYEQYF	2433	3	0.01%	NA	NA	NA	122	12	0.04%
HAM-10	CASSHPQGLNTEAFF	NA	NA	NA	NA	NA	NA	123	12	0.04%
HAM-10	CASSLDPLAGANNEQFF	NA	NA	NA	NA	NA	NA	124	12	0.04%
HAM-10	CASSSPLAVGYNEQFF	NA	NA	NA	2595	3	0.00%	125	12	0.04%
HAM-10	CASAPGLKGAADTQYF	13537	1	0.00%	1409	6	0.01%	126	12	0.04%
HAM-10	CATSPGLAGFYEQYF	NA	NA	NA	3988	2	0.00%	127	12	0.04%
HAM-10	CASANPLGGGARDTQYF	NA	NA	NA	NA	NA	NA	128	11	0.04%
HAM-10	CASSQGQGVNEQFF	13017	1	0.00%	4158	2	0.00%	129	11	0.04%
HAM-10	CASSWGLASAGNEQFF	1725	3	0.01%	2380	3	0.00%	130	11	0.04%
HAM-10	CASRPGLAGALDAQYF	11323	1	0.00%	1613	5	0.00%	131	11	0.04%
HAM-10	CASNPGLAGGTGELFF	NA	NA	NA	1196	7	0.01%	132	11	0.04%
HAM-10	CASSLGLAGGVEQFF	NA	NA	NA	NA	NA	NA	133	11	0.04%
HAM-10	CASSLSTGEVMNTEAFF	NA	NA	NA	NA	NA	NA	134	11	0.04%
HAM-10	CASSLGLGQGAPGYTF	NA	NA	NA	4069	2	0.00%	135	11	0.04%
HAM-10	CASTTGLKGAADTQYF	16284	1	0.00%	2187	4	0.00%	136	11	0.04%
HAM-10	CARLAGEGCEQYF	NA	NA	NA	1627	5	0.00%	137	11	0.04%
HAM-10	CASTPGVKGAADTQYF	NA	NA	NA	3638	2	0.00%	138	10	0.03%
HAM-10	CASTTLYGAGAGSPLHF	NA	NA	NA	NA	NA	NA	139	10	0.03%
HAM-10	CASSYSRSVGYNEQFF	17750	1	0.00%	NA	NA	NA	140	10	0.03%
HAM-10	CASSYGLEPSGANVLTF	NA	NA	NA	15539	1	0.00%	141	10	0.03%
HAM-10	CSAKAHEQYF	NA	NA	NA	NA	NA	NA	142	10	0.03%
HAM-10	CASSPGLAGGNEQFF	NA	NA	NA	2001	4	0.00%	143	10	0.03%
HAM-10	CASRGQGAVGYTF	NA	NA	NA	3034	3	0.00%	144	10	0.03%
HAM-10	CASSPGLATFPEQFF	NA	NA	NA	2188	4	0.00%	145	10	0.03%
HAM-10	CASSPGLAIATGELFF	2737	2	0.00%	1522	5	0.00%	146	10	0.03%
HAM-10	CASSQPFTSGSYEQYF	NA	NA	NA	NA	NA	NA	147	10	0.03%
HAM-10	CASSYSIGGSDYGYTF	NA	NA	NA	4122	2	0.00%	148	10	0.03%
HAM-10	CASRDPLAGGHTQYF	NA	NA	NA	NA	NA	NA	149	9	0.03%
HAM-10	CASSPGLAGAPGDAQYF	17920	1	0.00%	1252	7	0.01%	150	9	0.03%
HAM-10	CASSPREGSYEQYF	NA	NA	NA	NA	NA	NA	151	9	0.03%
HAM-10	CASSQVLGQGAAGELFF	NA	NA	NA	NA	NA	NA	152	9	0.03%
HAM-10	CASSLDRLFKNIQYF	NA	NA	NA	NA	NA	NA	153	9	0.03%
HAM-10	CASSQGQGATDTQYF	NA	NA	NA	3754	2	0.00%	154	8	0.03%
HAM-10	CASSYPGTGSFEQYF	NA	NA	NA	NA	NA	NA	155	8	0.03%
HAM-10	CASRPGLAGGVQNNEQFF	NA	NA	NA	NA	NA	NA	156	8	0.03%
HAM-10	CASSLHGQGSKAGELFF	16239	1	0.00%	2906	3	0.00%	157	8	0.03%
HAM-10	CASSPGLAGGRPPDTQYF	NA	NA	NA	1690	5	0.00%	158	8	0.03%
HAM-10	CASSDPGQGRNEQFF	NA	NA	NA	NA	NA	NA	159	8	0.03%
HAM-10	CASSPDARTMNYEQYF	NA	NA	NA	15882	1	0.00%	160	8	0.03%
HAM-10	CASSFGLAGGQGDAQYF	14592	1	0.00%	1205	7	0.01%	161	8	0.03%
HAM-10	CVSSPGQGVMDTQYF	NA	NA	NA	10167	1	0.00%	162	8	0.03%
HAM-10	CASSVGLAGGIVEQFF	NA	NA	NA	5235	1	0.00%	163	7	0.02%

HAM-10	CASSSSPWPSTDAQYF	14810	1	0.00%	2826	3	0.00%	164	7	0.02%
HAM-10	CASSPGQGATDTQYF	NA	NA	NA	NA	NA	NA	165	7	0.02%
HAM-10	CSAKSAEQYF	11268	1	0.00%	10372	1	0.00%	166	7	0.02%
HAM-10	CSAAGETQYF	NA	NA	NA	NA	NA	NA	167	7	0.02%
HAM-10	CASSPGLAEVKNIQYF	NA	NA	NA	3782	2	0.00%	168	7	0.02%
HAM-10	CASSQDPSSVGGGTEQYF	NA	NA	NA	NA	NA	NA	169	7	0.02%
HAM-10	CASSLRSPKGETEYF	19619	1	0.00%	1100	8	0.01%	170	7	0.02%
HAM-10	CASSYPGQGAYEQYF	NA	NA	NA	NA	NA	NA	171	7	0.02%
HAM-10	CASSWGLAGGPTTGELFF	NA	NA	NA	NA	NA	NA	172	7	0.02%
HAM-10	CASSPGLAGGRSDTQYF	14865	1	0.00%	2541	3	0.00%	173	7	0.02%
HAM-10	CASSLAPDTMNTEAFF	NA	NA	NA	2392	3	0.00%	174	7	0.02%
HAM-10	CASSPGLAGGETEYF	7023	1	0.00%	3343	2	0.00%	175	7	0.02%
HAM-10	CASSQDPAIQLNTEAFF	NA	NA	NA	NA	NA	NA	176	7	0.02%
HAM-10	CASSNGQGRQPQHF	NA	NA	NA	NA	NA	NA	177	7	0.02%
HAM-10	CASSQVLLQGMNSPLHF	NA	NA	NA	NA	NA	NA	178	7	0.02%
HAM-10	CASRPGLADGTDAQYF	NA	NA	NA	4879	2	0.00%	179	7	0.02%
HAM-10	CASTYLQGLEAEAFF	8	579	1.04%	6	3095	2.83%	180	7	0.02%
HAM-10	CASTAGLKGAADTQYF	NA	NA	NA	NA	NA	NA	181	7	0.02%
HAM-10	CASSQDNPGNGYTF	NA	NA	NA	10328	1	0.00%	182	6	0.02%
HAM-10	CASSHGLAGVYEQYF	NA	NA	NA	12453	1	0.00%	183	6	0.02%
HAM-10	CASSHGLAGDLEQYF	NA	NA	NA	3051	3	0.00%	184	6	0.02%
HAM-10	CASSCGLAGSFNNEQFF	14862	1	0.00%	13781	1	0.00%	185	6	0.02%
HAM-10	CASSEPLAGDYEQYF	NA	NA	NA	NA	NA	NA	186	6	0.02%
HAM-10	CASSLGQGHGYTF	NA	NA	NA	NA	NA	NA	187	6	0.02%
HAM-10	CASSETGYSPLHF	NA	NA	NA	NA	NA	NA	188	6	0.02%
HAM-10	CASSPGLAGGEAQYF	NA	NA	NA	4825	2	0.00%	189	6	0.02%
HAM-10	CASRSGLTPTSGNTIYF	19861	1	0.00%	NA	NA	NA	190	6	0.02%
HAM-10	CASSQDQLVQPQETQYF	NA	NA	NA	14883	1	0.00%	191	6	0.02%
HAM-10	CASSQGQGLNEQYF	NA	NA	NA	NA	NA	NA	192	6	0.02%
HAM-10	CASSQDPRHMNTEAFF	19898	1	0.00%	NA	NA	NA	193	6	0.02%
HAM-10	CASSYPGTGVYEQYF	3189	2	0.00%	2491	3	0.00%	194	6	0.02%
HAM-10	CASSVVSTTYEQYF	19925	1	0.00%	NA	NA	NA	195	6	0.02%
HAM-10	CASRQGLAGGTGELFF	NA	NA	NA	NA	NA	NA	196	6	0.02%
HAM-10	CASSPPDREMVNEQFF	3964	2	0.00%	1646	5	0.00%	197	6	0.02%
HAM-10	CASSDPRVSGTDTQYF	NA	NA	NA	NA	NA	NA	198	6	0.02%
HAM-10	CASSDLFDRGQESEQYF	NA	NA	NA	7587	1	0.00%	199	6	0.02%
HAM-10	CASSQEMGRDTEAFF	NA	NA	NA	NA	NA	NA	200	6	0.02%
HAM-10	CASSLGLGAKNIQYF	NA	NA	NA	NA	NA	NA	201	6	0.02%
HAM-10	CASSSLNPGDTIYF	NA	NA	NA	11067	1	0.00%	202	6	0.02%
HAM-10	CASSLGLAGVGNEQFF	NA	NA	NA	2045	4	0.00%	203	5	0.02%
HAM-10	CASSKPLASVHEQFF	4398	2	0.00%	NA	NA	NA	204	5	0.02%
HAM-10	CASNHGLAGGPGEQFF	NA	NA	NA	NA	NA	NA	205	5	0.02%
HAM-10	CSAPREGPTQPQHF	NA	NA	NA	2207	4	0.00%	206	5	0.02%
HAM-10	CASSIPLAGHYEQYF	NA	NA	NA	NA	NA	NA	207	5	0.02%
HAM-10	CASSPPPARMNTEAFF	NA	NA	NA	NA	NA	NA	208	5	0.02%
HAM-10	CASSQDWPQNEQFF	NA	NA	NA	NA	NA	NA	209	5	0.02%
HAM-10	CASSEALMFPEAFF	NA	NA	NA	NA	NA	NA	210	5	0.02%

HAM-10	CASSPTLAGGNEQFF	NA	NA	NA	NA	NA	NA	211	5	0.02%
HAM-10	CASSPRQGVMDTQYF	NA	NA	NA	13942	1	0.00%	212	5	0.02%
HAM-10	CASSSWLLRGGELFF	NA	NA	NA	NA	NA	NA	213	5	0.02%
HAM-10	CASSSQDAAVKYEQYF	NA	NA	NA	NA	NA	NA	214	5	0.02%
HAM-10	CASSYGLAGNNEQFF	NA	NA	NA	NA	NA	NA	215	5	0.02%
HAM-10	CASSSPGQGINEQFF	5404	2	0.00%	1497	6	0.01%	216	5	0.02%
HAM-10	CASSPPSRDMNTEAFF	NA	NA	NA	NA	NA	NA	217	5	0.02%
HAM-10	CARSPGLAGGGWETQYF	6110	1	0.00%	NA	NA	NA	218	5	0.02%
HAM-10	CSARDPQTGMNTEAFF	NA	NA	NA	NA	NA	NA	219	5	0.02%
HAM-10	CASSQEQGVNAEAFF	NA	NA	NA	4225	2	0.00%	220	5	0.02%
HAM-10	CASSDDEGRSMNTEAFF	NA	NA	NA	NA	NA	NA	221	5	0.02%
HAM-10	CASSLEMGPGEQYF	NA	NA	NA	NA	NA	NA	222	5	0.02%
HAM-10	CASRTGLADGTDTQYF	NA	NA	NA	12616	1	0.00%	223	5	0.02%
HAM-10	CASSDPGLGTDTQYF	543	9	0.02%	267	34	0.03%	224	5	0.02%
HAM-10	CASSFGLAGAFNNEQFF	NA	NA	NA	NA	NA	NA	225	5	0.02%
HAM-10	CATSDTGQGSDEQYF	19243	1	0.00%	NA	NA	NA	226	5	0.02%
HAM-10	CASSQATGGRAPFTF	NA	NA	NA	2779	3	0.00%	227	5	0.02%
HAM-10	CASSLDQGTDTQYF	3	1391	2.49%	3	4009	3.67%	228	5	0.02%
HAM-10	CASTPGLSGGSYEQYF	NA	NA	NA	4761	2	0.00%	229	5	0.02%
HAM-10	CASSPGQGGMDTQYF	NA	NA	NA	NA	NA	NA	230	5	0.02%
HAM-10	CASSHPQGRVADTQYF	8278	1	0.00%	NA	NA	NA	231	4	0.01%
HAM-10	CASSPGQGIMDTQYF	NA	NA	NA	NA	NA	NA	232	4	0.01%
HAM-10	CASSSPLVRPDQPQHF	NA	NA	NA	NA	NA	NA	233	4	0.01%
HAM-10	CASSELGVYGYTF	14762	1	0.00%	13695	1	0.00%	234	4	0.01%
HAM-10	CASSHGLAGIYEQYF	NA	NA	NA	NA	NA	NA	235	4	0.01%
HAM-10	CASSSPHRAGSSGNTIYF	NA	NA	NA	17150	1	0.00%	236	4	0.01%
HAM-10	CASRMGLAGGPEAQYF	NA	NA	NA	NA	NA	NA	237	4	0.01%
HAM-10	CASSTGQGVMDTQYF	NA	NA	NA	6264	1	0.00%	238	4	0.01%
HAM-10	CASSPNPRLMNTEAFF	NA	NA	NA	NA	NA	NA	239	4	0.01%
HAM-10	CASSQRPEYMNTEDFF	NA	NA	NA	13067	1	0.00%	240	4	0.01%
HAM-10	CASAQGLAGVNEQFF	NA	NA	NA	4180	2	0.00%	241	4	0.01%
HAM-10	CASSRGLAGRTGELFF	NA	NA	NA	NA	NA	NA	242	4	0.01%
HAM-10	CATSAWDNQPQHF	NA	NA	NA	NA	NA	NA	243	4	0.01%
HAM-10	CASSQPLAGTYEQYF	NA	NA	NA	NA	NA	NA	244	4	0.01%
HAM-10	CSARDRAGKEAQYF	9726	1	0.00%	8997	1	0.00%	245	4	0.01%
HAM-10	CASSSRLAGVLEQFF	NA	NA	NA	NA	NA	NA	246	4	0.01%
HAM-10	CASSSPREGNYGYAF	NA	NA	NA	16638	1	0.00%	247	4	0.01%
HAM-10	CASSQPPSEMNTEAFF	10745	1	0.00%	NA	NA	NA	248	4	0.01%
HAM-10	CASSLRAPKGETQYF	8870	1	0.00%	13021	1	0.00%	249	4	0.01%
HAM-10	CASSQGLSSGRVYAFF	NA	NA	NA	2650	3	0.00%	250	4	0.01%
HAM-10	CASGPGQGVMDTQYF	10752	1	0.00%	3825	2	0.00%	251	4	0.01%
HAM-10	CASSQRPEYMNTEAVF	7064	1	0.00%	1538	5	0.00%	252	4	0.01%
HAM-10	CASSPGLAGGQGASTDTQYF	NA	NA	NA	NA	NA	NA	253	4	0.01%
HAM-10	CASSSWLREGKLFF	15330	1	0.00%	2851	3	0.00%	254	4	0.01%
HAM-10	CASSQDPIFMNTEAFF	NA	NA	NA	17569	1	0.00%	255	4	0.01%
HAM-10	CASSLGQGRSPLHF	NA	NA	NA	NA	NA	NA	256	4	0.01%
HAM-10	CASSQDPHLMNTEAFF	NA	NA	NA	17633	1	0.00%	257	4	0.01%

HAM-10	CASSPGQGFTEAFF	NA	NA	NA	NA	NA	NA	258	4	0.01%
HAM-10	CASSPGLAGNTGELFF	3530	2	0.00%	6018	1	0.00%	259	4	0.01%
HAM-10	CSARDVGLNYGYAF	NA	NA	NA	NA	NA	NA	260	4	0.01%
HAM-10	CASSRGTGYQSQHF	NA	NA	NA	10947	1	0.00%	261	4	0.01%
HAM-10	CASSKGLAGGETQYF	NA	NA	NA	NA	NA	NA	262	4	0.01%
HAM-10	CASSTGLAGRTGELFF	10148	1	0.00%	NA	NA	NA	263	4	0.01%
HAM-10	CASSPGQGVMGTQYF	NA	NA	NA	NA	NA	NA	264	3	0.01%
HAM-10	CASSIEGQPQHF	NA	NA	NA	NA	NA	NA	265	3	0.01%
HAM-10	CASSLAGGRASAGGPLGAQYF	NA	NA	NA	4776	2	0.00%	266	3	0.01%
HAM-10	CASSFGLARATGELFF	NA	NA	NA	4905	2	0.00%	267	3	0.01%
HAM-10	CATSDDPLRGGYEQYF	NA	NA	NA	NA	NA	NA	268	3	0.01%
HAM-10	CASSFGLAGGSGDTQYF	NA	NA	NA	NA	NA	NA	269	3	0.01%
HAM-10	CASSLGQGVMDTQYF	NA	NA	NA	NA	NA	NA	270	3	0.01%
HAM-10	CSASEVLTSGSGEQFF	NA	NA	NA	NA	NA	NA	271	3	0.01%
HAM-10	CATSHGLAGINEQFF	NA	NA	NA	NA	NA	NA	272	3	0.01%
HAM-10	CSARDPGTGMNTEAFF	NA	NA	NA	NA	NA	NA	273	3	0.01%
HAM-10	CASKDPRQGVYGYTF	NA	NA	NA	NA	NA	NA	274	3	0.01%
HAM-10	CASSPGQGVMDTKYF	NA	NA	NA	NA	NA	NA	275	3	0.01%
HAM-10	CASSPGRGVMDAQYF	NA	NA	NA	3888	2	0.00%	276	3	0.01%
HAM-10	CASSRPLAGGNRVEQFF	NA	NA	NA	NA	NA	NA	277	3	0.01%
HAM-10	CASSPPPQSMNTEAFF	NA	NA	NA	11195	1	0.00%	278	3	0.01%
HAM-10	CARRPGLAGGGWETQYF	NA	NA	NA	NA	NA	NA	279	3	0.01%
HAM-10	CASSPGQGVMDTQDF	NA	NA	NA	12024	1	0.00%	280	3	0.01%
HAM-10	CASSYGLAGDNEQFF	NA	NA	NA	NA	NA	NA	281	3	0.01%
HAM-10	CASSSPGTSGRPYEQYF	NA	NA	NA	NA	NA	NA	282	3	0.01%
HAM-10	CASSPVRGAHNEQFF	NA	NA	NA	NA	NA	NA	283	3	0.01%
HAM-10	CASSQVPAMNTEAFF	NA	NA	NA	3178	2	0.00%	284	3	0.01%
HAM-10	CASSFGLAGSFSNEQFF	NA	NA	NA	NA	NA	NA	285	3	0.01%
HAM-10	CASSAGLAGGIYEQYF	NA	NA	NA	13851	1	0.00%	286	3	0.01%
HAM-10	CASSQDVSVMNTEAFF	NA	NA	NA	NA	NA	NA	287	3	0.01%
HAM-10	CSARAKGVKEQFF	56	69	0.12%	29	433	0.40%	288	3	0.01%
HAM-10	CASTPGLAGGSTDTQYF	NA	NA	NA	NA	NA	NA	289	3	0.01%
HAM-10	CASSMGQGGWQPQHF	2505	3	0.01%	647	14	0.01%	290	3	0.01%
HAM-10	CASSQDPARLNTEAFF	NA	NA	NA	16391	1	0.00%	291	3	0.01%
HAM-10	CASSVAGGRASAGGPLGTQYF	NA	NA	NA	NA	NA	NA	292	3	0.01%
HAM-10	CASSSPGTGNYGYTF	13201	1	0.00%	2744	3	0.00%	293	3	0.01%
HAM-10	CASSSPGQGQETQYF	NA	NA	NA	NA	NA	NA	294	3	0.01%
HAM-10	CASSPGQGVMDTRYF	NA	NA	NA	NA	NA	NA	295	3	0.01%
HAM-10	CASSQAAESSYEQYF	46	88	0.16%	26	448	0.41%	296	3	0.01%
HAM-10	CASAPGLKGAADAQYF	NA	NA	NA	NA	NA	NA	297	3	0.01%
HAM-10	CASSPGQGVMNAQYF	12477	1	0.00%	11590	1	0.00%	298	3	0.01%
HAM-10	CATSAPGQGSDEQFF	NA	NA	NA	NA	NA	NA	299	3	0.01%
HAM-10	CSARSGQEVF	NA	NA	NA	NA	NA	NA	300	3	0.01%
HAM-10	CASSQVFTSGSNEQFF	NA	NA	NA	NA	NA	NA	301	3	0.01%
HAM-10	CASSLRSPKGETQFF	7942	1	0.00%	7404	1	0.00%	302	3	0.01%
HAM-10	CASSPTATDMNTEAFF	NA	NA	NA	NA	NA	NA	303	3	0.01%
HAM-10	CASSSPLAGLLEQFF	NA	NA	NA	NA	NA	NA	304	3	0.01%

HAM-10	CASSLPLAGVWEQFF	NA	NA	NA	NA	NA	NA	305	3	0.01%
HAM-10	CASSPGLAGGETQCF	NA	NA	NA	NA	NA	NA	306	3	0.01%
HAM-10	CASSQVLRGGAGETQYF	NA	NA	NA	NA	NA	NA	307	3	0.01%
HAM-10	CASIPGLAGRAEQFF	NA	NA	NA	16739	1	0.00%	308	3	0.01%
HAM-10	CASSHNRDRGYTEAFF	NA	NA	NA	NA	NA	NA	309	3	0.01%
HAM-10	CASSERITSQGDTQYF	NA	NA	NA	NA	NA	NA	310	3	0.01%
HAM-10	CASSPGEGVMDTQYF	NA	NA	NA	NA	NA	NA	311	3	0.01%
HAM-10	CASSQPLAGGYEQYF	NA	NA	NA	NA	NA	NA	312	3	0.01%
HAM-10	CASSPGQGLNIQYF	NA	NA	NA	NA	NA	NA	313	3	0.01%
HAM-10	CASSPGQRVMDTQYF	NA	NA	NA	6260	1	0.00%	314	3	0.01%
HAM-10	CASSRGQGVMDAQYF	NA	NA	NA	NA	NA	NA	315	3	0.01%
HAM-10	CASSPGQGGMDAQYF	NA	NA	NA	NA	NA	NA	316	3	0.01%
HAM-10	CASSQDPRAMNYEQYF	NA	NA	NA	NA	NA	NA	317	3	0.01%
HAM-10	CASSQRPEYMNAEAFF	8218	1	0.00%	2455	3	0.00%	318	3	0.01%
HAM-10	CASSEPLTGDYEQYF	NA	NA	NA	NA	NA	NA	319	3	0.01%
HAM-10	CASSPGLAGGYNEQFF	NA	NA	NA	NA	NA	NA	320	3	0.01%
HAM-10	CAWLPQYF	NA	NA	NA	NA	NA	NA	321	3	0.01%
HAM-10	CASTPGLKGAEDTQYF	NA	NA	NA	NA	NA	NA	322	3	0.01%
HAM-10	CASTRGLKGAADTQYF	NA	NA	NA	14418	1	0.00%	323	2	0.01%
HAM-10	CASRGQAGRAAFF	NA	NA	NA	NA	NA	NA	324	2	0.01%
HAM-10	CASSTTGTLIPEAFF	NA	NA	NA	NA	NA	NA	325	2	0.01%
HAM-10	CASSLRSPKGETRYF	NA	NA	NA	2867	3	0.00%	326	2	0.01%
HAM-10	CASSHGQRLTDTQYF	NA	NA	NA	NA	NA	NA	327	2	0.01%
HAM-10	CASSCSPWPSTDTQYF	NA	NA	NA	6684	1	0.00%	328	2	0.01%
HAM-10	CATLAGLAGGRTQYF	NA	NA	NA	5159	1	0.00%	329	2	0.01%
HAM-10	CASSVGQGADTQYF	1574	4	0.01%	3002	3	0.00%	330	2	0.01%
HAM-10	CASSRGPGQGHQPQHF	NA	NA	NA	NA	NA	NA	331	2	0.01%
HAM-10	CASSQPLAGEYEQYF	NA	NA	NA	NA	NA	NA	332	2	0.01%
HAM-10	CASSQVPQQMNTEAFF	NA	NA	NA	NA	NA	NA	333	2	0.01%
HAM-10	CASSYSIGTAYQPQHF	NA	NA	NA	NA	NA	NA	334	2	0.01%
HAM-10	CVSRPGLAGALDTQYF	NA	NA	NA	NA	NA	NA	335	2	0.01%
HAM-10	CASSQRPEHMNTEAFF	NA	NA	NA	NA	NA	NA	336	2	0.01%
HAM-10	CASTQGLAGGPNTGELFF	NA	NA	NA	12004	1	0.00%	337	2	0.01%
HAM-10	CASAPGLAGGRSTDTQYF	NA	NA	NA	17045	1	0.00%	338	2	0.01%
HAM-10	CASSPGLAGGLHEQFF	11162	1	0.00%	NA	NA	NA	339	2	0.01%
HAM-10	CANRPGLADGTDTQYF	NA	NA	NA	NA	NA	NA	340	2	0.01%
HAM-10	CASSPRQGVMDAQYF	NA	NA	NA	NA	NA	NA	341	2	0.01%
HAM-10	CASSQGLAAAKNIQYF	NA	NA	NA	NA	NA	NA	342	2	0.01%
HAM-10	CASSQGLAGGPEQFF	NA	NA	NA	13712	1	0.00%	343	2	0.01%
HAM-10	CASSQEQGVNSEAFF	NA	NA	NA	NA	NA	NA	344	2	0.01%
HAM-10	CASSTLGRGDYEQYF	NA	NA	NA	NA	NA	NA	345	2	0.01%
HAM-10	CATSDQTVRELFF	NA	NA	NA	NA	NA	NA	346	2	0.01%
HAM-10	CASSFGIAGSFNNEQFF	NA	NA	NA	NA	NA	NA	347	2	0.01%
HAM-10	CASSLRSPKGETQCF	NA	NA	NA	4276	2	0.00%	348	2	0.01%
HAM-10	CASSPGLAGTNEQFF	NA	NA	NA	4019	2	0.00%	349	2	0.01%
HAM-10	CVSSPGLAGRTGELFF	NA	NA	NA	NA	NA	NA	350	2	0.01%
HAM-10	CASTHPLAGDYEQYF	NA	NA	NA	NA	NA	NA	351	2	0.01%

HAM-10	CASSFGLAGSFNSEQFF	NA	NA	NA	NA	NA	NA	352	2	0.01%
HAM-10	CSARSGEDVF	NA	NA	NA	12988	1	0.00%	353	2	0.01%
HAM-10	CASSRGGNEQFF	NA	NA	NA	NA	NA	NA	354	2	0.01%
HAM-10	CASSQGQRITDAQYF	NA	NA	NA	NA	NA	NA	355	2	0.01%
HAM-10	CASSQEQGVNTEEFF	NA	NA	NA	NA	NA	NA	356	2	0.01%
HAM-10	CASTQGLKGAADTQYF	NA	NA	NA	NA	NA	NA	357	2	0.01%
HAM-10	CASTPGLKGSADTQYF	NA	NA	NA	NA	NA	NA	358	2	0.01%
HAM-10	CASSLRSPKGENQYF	NA	NA	NA	NA	NA	NA	359	2	0.01%
HAM-10	CASSASPWPSTDAQYF	NA	NA	NA	NA	NA	NA	360	2	0.01%
HAM-10	CASSAGQGVMDTQYF	NA	NA	NA	NA	NA	NA	361	2	0.01%
HAM-10	CASSSGLAGGIYEQFF	NA	NA	NA	4550	2	0.00%	362	2	0.01%
HAM-10	CASSPGLAGGRNTQYF	NA	NA	NA	4936	2	0.00%	363	2	0.01%
HAM-10	CASSITSGRATEQFF	NA	NA	NA	NA	NA	NA	364	2	0.01%
HAM-10	CASSPGLKGAADTQYF	NA	NA	NA	2082	4	0.00%	365	2	0.01%
HAM-10	CASSEPHVGDTRNEQFF	NA	NA	NA	NA	NA	NA	366	2	0.01%
HAM-10	CASSPGQGVRDAQYF	NA	NA	NA	NA	NA	NA	367	2	0.01%
HAM-10	CASSQRTEYMNTEAFF	NA	NA	NA	3538	2	0.00%	368	2	0.01%
HAM-10	CASSEPFRGLNYGYTF	NA	NA	NA	10507	1	0.00%	369	2	0.01%
HAM-10	CASRPGLAGVLDTQYF	NA	NA	NA	NA	NA	NA	370	2	0.01%
HAM-10	CASSQEQGVNTEDFF	NA	NA	NA	NA	NA	NA	371	2	0.01%
HAM-10	CASSPEGNEQFF	54	72	0.13%	40	299	0.27%	372	2	0.01%
HAM-10	CASSFGLVGSFNNEQFF	NA	NA	NA	NA	NA	NA	373	2	0.01%
HAM-10	CASSPGLAGRTGELLF	NA	NA	NA	NA	NA	NA	374	2	0.01%
HAM-10	CASSPGQGVMDTEYF	16984	1	0.00%	NA	NA	NA	375	2	0.01%
HAM-10	CASTFELGNEKLFF	6	692	1.24%	5	3104	2.84%	376	2	0.01%
HAM-10	CASSPGQGVMGAQYF	NA	NA	NA	NA	NA	NA	377	2	0.01%
HAM-10	CAISESISGSTETQYF	NA	NA	NA	NA	NA	NA	378	2	0.01%
HAM-10	CASGPGLAGGGWETQYF	NA	NA	NA	NA	NA	NA	379	2	0.01%
HAM-10	CASSQVLQRGASGEQFF	NA	NA	NA	NA	NA	NA	380	2	0.01%
HAM-10	CASSPGLAGGEAEYF	NA	NA	NA	11577	1	0.00%	381	2	0.01%
HAM-10	CASSPGLAGGEDQYF	NA	NA	NA	NA	NA	NA	382	2	0.01%
HAM-10	CASSPGRGVMDTQYF	NA	NA	NA	NA	NA	NA	383	2	0.01%
HAM-10	CASSDPGQGSFEQFF	NA	NA	NA	NA	NA	NA	384	2	0.01%
HAM-10	CSSSPGLAGAPGDTQYF	NA	NA	NA	NA	NA	NA	385	2	0.01%
HAM-10	CASSWGSHNEQFF	694	7	0.01%	782	11	0.01%	386	2	0.01%
HAM-10	CASSQVVQRGASGEQYF	NA	NA	NA	NA	NA	NA	387	2	0.01%
HAM-10	CASSVASSGNTIYF	NA	NA	NA	2342	3	0.00%	388	2	0.01%
HAM-10	CASNPGLAGGAYNEQFF	NA	NA	NA	NA	NA	NA	389	2	0.01%
HAM-10	CASRGGLREAKNIQYF	NA	NA	NA	NA	NA	NA	390	2	0.01%
HAM-10	CASSNPRQGQYEQYF	NA	NA	NA	NA	NA	NA	391	2	0.01%
HAM-10	CASTPGQGVMDTQYF	NA	NA	NA	NA	NA	NA	392	2	0.01%
HAM-10	CASSPRPEPQHF	4495	2	0.00%	1433	6	0.01%	393	2	0.01%
HAM-10	CASSQRPEYMNTEEFF	NA	NA	NA	NA	NA	NA	394	2	0.01%
HAM-10	CASSSGDPSAAYRYTF	NA	NA	NA	NA	NA	NA	395	2	0.01%
HAM-10	CASTPGLKGAANTQYF	NA	NA	NA	5089	2	0.00%	396	2	0.01%
HAM-10	CASSQRTECMNTEAFF	NA	NA	NA	NA	NA	NA	397	2	0.01%
HAM-10	CASSLRSPKGEAEYF	NA	NA	NA	NA	NA	NA	398	2	0.01%

HAM-10	CASSQVLGQGLNSPLHF	16176	1	0.00%	NA	NA	NA	399	2	0.01%
HAM-10	CASSFPLRGKTEAFF	NA	NA	NA	NA	NA	NA	400	2	0.01%
HAM-10	CASSPGLAGGPREQYF	NA	NA	NA	NA	NA	NA	401	2	0.01%
HAM-10	CASSPGQGVLDTQYF	NA	NA	NA	16628	1	0.00%	402	2	0.01%
HAM-10	CASSPGLAGGETQFF	NA	NA	NA	NA	NA	NA	403	2	0.01%
HAM-10	CASSGGLAGTKGQFF	NA	NA	NA	NA	NA	NA	404	2	0.01%
HAM-10	CASSVGLAGVTGELFF	NA	NA	NA	NA	NA	NA	405	2	0.01%
HAM-10	CASVPGLAGGGNEQFF	NA	NA	NA	NA	NA	NA	406	2	0.01%
HAM-10	CASSPGLAGAAGDTQYF	NA	NA	NA	5739	1	0.00%	407	2	0.01%
HAM-10	CASSLDPGQGFYEQYF	NA	NA	NA	NA	NA	NA	408	2	0.01%
HAM-10	CTSSLRSPKGETQYF	NA	NA	NA	NA	NA	NA	409	2	0.01%
HAM-10	CASTLGLAGVAGTDTQYF	NA	NA	NA	NA	NA	NA	410	2	0.01%
HAM-10	CASSLRSPKGGTQYF	NA	NA	NA	NA	NA	NA	411	2	0.01%
HAM-10	CASSQRAEYMNTEAFF	7818	1	0.00%	17921	1	0.00%	412	2	0.01%
HAM-10	CASSRRPEYMNTEAFF	NA	NA	NA	NA	NA	NA	413	2	0.01%
HAM-10	CASSSPGQGTYEQYF	NA	NA	NA	16736	1	0.00%	414	2	0.01%
HAM-10	CASSIFGGGIGYTF	NA	NA	NA	NA	NA	NA	415	2	0.01%
HAM-10	CASSLRSPKGEAQSF	NA	NA	NA	5777	1	0.00%	416	2	0.01%
HAM-10	CASSQPLFTGSYEQYF	NA	NA	NA	NA	NA	NA	417	2	0.01%
HAM-10	CASSNPGQGQYEQYF	NA	NA	NA	NA	NA	NA	418	2	0.01%
HAM-10	CASSYPGRGVNEQFF	NA	NA	NA	NA	NA	NA	419	2	0.01%
HAM-10	CASTPGLEGAADTQYF	NA	NA	NA	NA	NA	NA	420	2	0.01%
HAM-10	CASGPGQGVMDAQYF	NA	NA	NA	NA	NA	NA	421	2	0.01%
HAM-10	CASSSTLVRQGYTDTQYF	NA	NA	NA	NA	NA	NA	422	2	0.01%
HAM-10	CASSLRSPKGETQDF	NA	NA	NA	NA	NA	NA	423	2	0.01%
HAM-10	CASSPGLAGGLATSTDTQYF	NA	NA	NA	1553	5	0.00%	424	2	0.01%
HAM-10	CASSLDPLECGMNTEAFF	NA	NA	NA	NA	NA	NA	425	2	0.01%
HAM-10	CASSPGQGVMDPQYF	NA	NA	NA	NA	NA	NA	426	2	0.01%
HAM-10	CASSLAGSGSVNEQFF	80	49	0.09%	64	180	0.16%	427	2	0.01%
HAM-10	CASSFGLAGSVNNEQFF	NA	NA	NA	13808	1	0.00%	428	2	0.01%
HAM-10	CASSPAQGVMDTQYF	NA	NA	NA	NA	NA	NA	429	2	0.01%
HAM-10	CASTNPMRTGGMEAFF	NA	NA	NA	NA	NA	NA	430	2	0.01%
HAM-10	CASSSPGIGVEAQYF	NA	NA	NA	NA	NA	NA	431	2	0.01%
HAM-10	CASSWSGSGYTF	NA	NA	NA	NA	NA	NA	432	2	0.01%
HAM-10	CASSVRSPKGETQYF	NA	NA	NA	1947	4	0.00%	433	2	0.01%
HAM-10	CASSPLGRGEYEQYF	NA	NA	NA	NA	NA	NA	434	2	0.01%
HAM-10	CAGLGGRDQETQYF	NA	NA	NA	16744	1	0.00%	435	2	0.01%
HAM-10	CASSAGLSGANVLTF	NA	NA	NA	NA	NA	NA	436	2	0.01%
HAM-10	CASSSGLAGGSYEQYF	NA	NA	NA	18593	1	0.00%	437	2	0.01%
HAM-10	CASSPGPGQGHQPEHF	NA	NA	NA	NA	NA	NA	438	2	0.01%
HAM-10	CASSSPGTGREAQYF	NA	NA	NA	NA	NA	NA	439	2	0.01%
HAM-10	CASSLSLAGGQPGELFF	NA	NA	NA	9354	1	0.00%	440	2	0.01%
HAM-10	CASSYPGTGQETQYF	NA	NA	NA	11546	1	0.00%	441	2	0.01%
HAM-10	CASSSGLAGGMYEQYF	NA	NA	NA	NA	NA	NA	442	2	0.01%
HAM-10	CSARDQTSGSIDTQYF	230	18	0.03%	162	60	0.05%	443	2	0.01%
HAM-10	CASSLGYRGMNTEAFF	NA	NA	NA	11020	1	0.00%	444	2	0.01%
HAM-10	CASNSGLAGGIYEQYF	NA	NA	NA	NA	NA	NA	445	2	0.01%

HAM-10	CASSFPFKGIYGYTF	NA	NA	NA	NA	NA	NA	446	2	0.01%
HAM-10	CASTQGLSGANVLTF	NA	NA	NA	NA	NA	NA	447	2	0.01%
HAM-10	CASSLGLAGNSDTQYF	NA	NA	NA	NA	NA	NA	448	2	0.01%
HAM-10	CASSQEQGVNTEAVF	NA	NA	NA	3867	2	0.00%	449	2	0.01%
HAM-10	CASSEPLTGNYEQYF	NA	NA	NA	5973	1	0.00%	450	2	0.01%
HAM-10	CASSETGGYEQYF	75	51	0.09%	44	275	0.25%	451	2	0.01%
HAM-10	CASIRGLSSTNTEAFF	NA	NA	NA	7689	1	0.00%	452	2	0.01%
HAM-10	CASSPGLRGGNTIYF	NA	NA	NA	NA	NA	NA	453	2	0.01%
HAM-10	CASSLRSPKGEIQYF	NA	NA	NA	NA	NA	NA	454	2	0.01%
HAM-10	CASSSDRTLNTEAFF	111	35	0.06%	48	236	0.22%	455	2	0.01%
HAM-11	CASSDPLTGHYEQYF	7	614	0.99%	4	2403	2.81%	1	792	60.32%
HAM-11	CASTDIRFRGLGTEAFF	17	229	0.37%	17	685	0.80%	2	181	13.79%
HAM-11	CASSHPPAGGANTQYF	680	11	0.02%	105	59	0.07%	3	77	5.86%
HAM-11	CATSDAGSGGNTIYF	342	24	0.04%	39	215	0.25%	4	56	4.27%
HAM-11	CASSSPLAGDYEQYF	637	12	0.02%	88	74	0.09%	5	31	2.36%
HAM-11	CASSSPFSRGAADTQYF	782	9	0.01%	86	77	0.09%	6	24	1.83%
HAM-11	CASSYPLAGKYEQYF	394	21	0.03%	52	155	0.18%	7	23	1.75%
HAM-11	CASSQVLGEGAAGELFF	1140	5	0.01%	221	19	0.02%	8	19	1.45%
HAM-11	CASSFGLAGGSTDTQYF	270	30	0.05%	51	157	0.18%	9	18	1.37%
HAM-11	CASSSPLTGLYGYTF	975	6	0.01%	196	23	0.03%	10	14	1.07%
HAM-11	CASSAGLAGNNEQFF	776	9	0.01%	115	52	0.06%	11	12	0.91%
HAM-11	CASSQPQGLNTEAFF	1116	5	0.01%	166	30	0.04%	12	11	0.84%
HAM-11	CASSIHANRGDTEAFF	NA	NA	NA	552	6	0.01%	13	10	0.76%
HAM-11	CASSLEPLAGGGNTEAFF	2949	2	0.00%	249	16	0.02%	14	6	0.46%
HAM-11	CASNDPQAGTNLGYTF	NA	NA	NA	531	6	0.01%	15	5	0.38%
HAM-11	CASSPGLAGGRHNEQFF	17277	1	0.00%	426	8	0.01%	16	4	0.30%
HAM-11	CASSSGLSGANVLTF	1020	6	0.01%	534	6	0.01%	17	3	0.23%
HAM-11	CASSPGLAGSSYEQYF	NA	NA	NA	243	17	0.02%	18	3	0.23%
HAM-11	CASSPGQGASPLHF	NA	NA	NA	248	16	0.02%	19	3	0.23%
HAM-11	CASSWGSHNEQFF	1	5812	9.38%	1	21588	25.27%	20	2	0.15%
HAM-11	CSAKASEQYF	NA	NA	NA	1518	2	0.00%	21	2	0.15%
HAM-11	CASSPLTILNNEQFF	NA	NA	NA	NA	NA	NA	22	2	0.15%
HAM-11	CASSDTLTGHYEQYF	NA	NA	NA	6346	1	0.00%	23	2	0.15%
HAM-12	CATSRITSGEPYEQYF	13	117	0.31%	13	367	0.82%	1	1465	16.05%
HAM-12	CASRPGLAEAKNIQYF	37	44	0.12%	49	82	0.18%	2	566	6.20%
HAM-12	CASSYGLAGEPEQFF	NA	NA	NA	53	71	0.16%	3	447	4.90%
HAM-12	CASSPLSYNEQFF	238	11	0.03%	57	64	0.14%	4	403	4.41%
HAM-12	CASSQEQGLNTEAFF	109	20	0.05%	67	52	0.12%	5	369	4.04%
HAM-12	CASSQEARSLRVEQYF	NA	NA	NA	88	39	0.09%	6	329	3.60%
HAM-12	CASSLPVGEMNTEAFF	144	17	0.04%	87	40	0.09%	7	309	3.38%
HAM-12	CASRPGLAAPTGELFF	309	10	0.03%	63	57	0.13%	8	296	3.24%
HAM-12	CASSQTGLLTHNEQFF	2428	2	0.01%	109	31	0.07%	9	230	2.52%
HAM-12	CASNPGLAGGSTDTQYF	NA	NA	NA	NA	NA	NA	10	219	2.40%
HAM-12	CASSLDPLAGGYNEQFF	466	7	0.02%	106	32	0.07%	11	201	2.20%
HAM-12	CSAAQETQYF	288	10	0.03%	71	49	0.11%	12	190	2.08%
HAM-12	CASTQGLSGANVLTF	149	17	0.04%	54	71	0.16%	13	115	1.26%
HAM-12	CASGPGLASAKNIQYF	199	13	0.03%	98	35	0.08%	14	103	1.13%

HAM-12	CASSIWGYNEQFF	NA	NA	NA	150	19	0.04%	15	99	1.08%
HAM-12	CSASTDTQYF	NA	NA	NA	219	12	0.03%	16	99	1.08%
HAM-12	CSASSGDTQYF	3087	2	0.01%	166	17	0.04%	17	98	1.07%
HAM-12	CATLPGLAGGFNEQFF	664	5	0.01%	147	20	0.04%	18	90	0.99%
HAM-12	CASRPGLAGASDTQYF	NA	NA	NA	99	35	0.08%	19	89	0.97%
HAM-12	CASSVGQGRNEQYF	2174	2	0.01%	94	37	0.08%	20	87	0.95%
HAM-12	CASSDWYNGGANSPLHF	6027	1	0.00%	314	8	0.02%	21	86	0.94%
HAM-12	CASSLPLAGVHEQYF	NA	NA	NA	173	16	0.04%	22	82	0.90%
HAM-12	CASRPGLAGGPRGDTQYF	573	6	0.02%	142	21	0.05%	23	81	0.89%
HAM-12	CASSSPGQGYNEQFF	2597	2	0.01%	113	28	0.06%	24	81	0.89%
HAM-12	CASSAPPQDMNTEAFF	703	5	0.01%	195	13	0.03%	25	78	0.85%
HAM-12	CASSEEVGTPYEQYF	807	4	0.01%	169	16	0.04%	26	76	0.83%
HAM-12	CASSSPLQGGVSSPLHF	2795	2	0.01%	228	11	0.02%	27	76	0.83%
HAM-12	CASSLWGGAQPQHF	490	7	0.02%	132	22	0.05%	28	75	0.82%
HAM-12	CASSLAGGFGEQFF	1442	3	0.01%	111	29	0.06%	29	74	0.81%
HAM-12	CSAKANEQYF	NA	NA	NA	315	8	0.02%	30	73	0.80%
HAM-12	CATREGLAGANVNEQFF	416	8	0.02%	157	18	0.04%	31	65	0.71%
HAM-12	CASSDPLNTGAGGYTF	NA	NA	NA	346	7	0.02%	32	63	0.69%
HAM-12	CATSGGLAGGHETQYF	808	4	0.01%	211	12	0.03%	33	62	0.68%
HAM-12	CASRAGLAGGVEQFF	569	6	0.02%	134	22	0.05%	34	61	0.67%
HAM-12	CASSQDPLASGANNEQFF	356	9	0.02%	176	16	0.04%	35	60	0.66%
HAM-12	CASSNPGRGSYEQYF	505	7	0.02%	124	25	0.06%	36	58	0.64%
HAM-12	CASSQPQGMNTEAFF	NA	NA	NA	183	14	0.03%	37	57	0.62%
HAM-12	CSARDLAGGAVIGNTQYF	675	5	0.01%	269	9	0.02%	38	57	0.62%
HAM-12	CASSLDPLFMNTEAFF	625	5	0.01%	226	11	0.02%	39	56	0.61%
HAM-12	CASRPGLAGGTLGADTQYF	1588	3	0.01%	153	19	0.04%	40	52	0.57%
HAM-12	CASSYPGTGQYEQFF	1519	3	0.01%	135	22	0.05%	41	49	0.54%
HAM-12	CASSLGFGGRGNTIYF	1206	3	0.01%	243	11	0.02%	42	46	0.50%
HAM-12	CSASYGDTQYF	11187	1	0.00%	510	4	0.01%	43	41	0.45%
HAM-12	CASSFLGGGQETQYF	NA	NA	NA	203	13	0.03%	44	39	0.43%
HAM-12	CASSSPLAGVYEQFF	NA	NA	NA	334	7	0.02%	45	38	0.42%
HAM-12	CASSPGLAGGTSGANVLTF	556	6	0.02%	75	48	0.11%	46	38	0.42%
HAM-12	CASSPGLAAAIVGQFF	429	7	0.02%	234	11	0.02%	47	37	0.41%
HAM-12	CASSEVLISGSAGELFF	NA	NA	NA	644	3	0.01%	48	35	0.38%
HAM-12	CSARSGEDTF	1209	3	0.01%	694	3	0.01%	49	35	0.38%
HAM-12	CSAKASEQFF	9830	1	0.00%	318	7	0.02%	50	34	0.37%
HAM-12	CSADGTSASEQFF	NA	NA	NA	265	9	0.02%	51	33	0.36%
HAM-12	CASSLGLAGPRGDEQFF	565	6	0.02%	185	14	0.03%	52	31	0.34%
HAM-12	CSGRLGTEAFF	2342	2	0.01%	449	5	0.01%	53	31	0.34%
HAM-12	CASSEPLTGAYEQYF	623	6	0.02%	224	12	0.03%	54	31	0.34%
HAM-12	CSAKAAEQFF	NA	NA	NA	143	21	0.05%	55	30	0.33%
HAM-12	CASSQGQGQDEQYF	8963	1	0.00%	537	4	0.01%	56	29	0.32%
HAM-12	CASSYGLAGGPKPYNEQFF	NA	NA	NA	546	4	0.01%	57	28	0.31%
HAM-12	CASNPGLAGGHEQFF	649	5	0.01%	253	10	0.02%	58	24	0.26%
HAM-12	CASSEPLTGSYEQYF	2865	2	0.01%	406	5	0.01%	59	24	0.26%
HAM-12	CASSTPGTGVYEQYF	2774	2	0.01%	286	9	0.02%	60	24	0.26%
HAM-12	CASSSPGQGTPEQYF	NA	NA	NA	155	18	0.04%	61	22	0.24%

HAM-12	CSARADRDFF	NA	NA	NA	943	2	0.00%	62	21	0.23%
HAM-12	CASRPGLAGGPGGDTQYF	NA	NA	NA	304	8	0.02%	63	20	0.22%
HAM-12	CASRPGLAGGEASDTQYF	2088	2	0.01%	362	6	0.01%	64	20	0.22%
HAM-12	CASSYGLAGGIEQFF	560	6	0.02%	223	12	0.03%	65	19	0.21%
HAM-12	CASSQDPQVMDTEAFF	NA	NA	NA	595	3	0.01%	66	18	0.20%
HAM-12	CSALYTGLSYEQYF	1988	2	0.01%	555	3	0.01%	67	18	0.20%
HAM-12	CASSRAVGTGDYEQYF	770	5	0.01%	238	11	0.02%	68	18	0.20%
HAM-12	CASSQDTLTGAYNSPLHF	NA	NA	NA	NA	NA	NA	69	18	0.20%
HAM-12	CASSLGVPEQFF	NA	NA	NA	2403	1	0.00%	70	17	0.19%
HAM-12	CASSSPLDGIYGYTF	3730	1	0.00%	768	2	0.00%	71	17	0.19%
HAM-12	CASSYPGQGAYEQYF	NA	NA	NA	351	6	0.01%	72	16	0.18%
HAM-12	CASSSGLAGDGSEQYF	3864	1	0.00%	408	5	0.01%	73	16	0.18%
HAM-12	CASSQVMGVNTEAFF	2197	2	0.01%	300	8	0.02%	74	16	0.18%
HAM-12	CASSPPAEDMNTEAFF	NA	NA	NA	3233	1	0.00%	75	15	0.16%
HAM-12	CASSSAGGFGELFF	NA	NA	NA	387	6	0.01%	76	15	0.16%
HAM-12	CASSQDPLAGGYNEQFF	NA	NA	NA	567	3	0.01%	77	15	0.16%
HAM-12	CASSPPPGGANTGELFF	NA	NA	NA	NA	NA	NA	78	15	0.16%
HAM-12	CASRPGQGGELFF	1	5738	15.02%	1	15444	34.40%	79	15	0.16%
HAM-12	CASNKGLAGGPRGNEQFF	1111	3	0.01%	321	7	0.02%	80	15	0.16%
HAM-12	CASSRPLAGRYEQYF	NA	NA	NA	391	5	0.01%	81	14	0.15%
HAM-12	CASSNPLEGANYGYTF	NA	NA	NA	3251	1	0.00%	82	14	0.15%
HAM-12	CASSPGLAGGGETQYF	NA	NA	NA	502	4	0.01%	83	14	0.15%
HAM-12	CASSSDPLAGGYNEQFF	1995	2	0.01%	708	2	0.00%	84	14	0.15%
HAM-12	CASSPGLAGVGEQFF	1774	2	0.01%	326	7	0.02%	85	14	0.15%
HAM-12	CASSQGQGTTDTQYF	NA	NA	NA	NA	NA	NA	86	14	0.15%
HAM-12	CASSFPLAGDHEQYF	769	5	0.01%	435	5	0.01%	87	13	0.14%
HAM-12	CASSRPLGGVYEQYF	14562	1	0.00%	379	6	0.01%	88	12	0.13%
HAM-12	CASSGGLAGARETQYF	NA	NA	NA	1991	1	0.00%	89	12	0.13%
HAM-12	CASSQSWPYHNEQFF	NA	NA	NA	NA	NA	NA	90	11	0.12%
HAM-12	CASSPGLAGAGHEQYF	NA	NA	NA	NA	NA	NA	91	11	0.12%
HAM-12	CASSRWGQGSTDTQYF	1163	3	0.01%	525	4	0.01%	92	11	0.12%
HAM-12	CASTHGLAGHTRSYNEQFF	2658	2	0.01%	526	4	0.01%	93	11	0.12%
HAM-12	CSASYGAEAFF	12577	1	0.00%	3361	1	0.00%	94	11	0.12%
HAM-12	CASSLGQGKNEQFF	11764	1	0.00%	983	2	0.00%	95	11	0.12%
HAM-12	CASSPVTVTGSNIQYF	NA	NA	NA	NA	NA	NA	96	11	0.12%
HAM-12	CASSVGQGTGELFF	NA	NA	NA	333	7	0.02%	97	10	0.11%
HAM-12	CASSQPLAGAHEQYF	NA	NA	NA	NA	NA	NA	98	10	0.11%
HAM-12	CASSPGLAGGETQYF	10855	1	0.00%	3315	1	0.00%	99	10	0.11%
HAM-12	CASSSPGQGSYEQYF	10866	1	0.00%	NA	NA	NA	100	10	0.11%
HAM-12	CASSESIGDNEQFF	2	1082	2.83%	2	4281	9.54%	101	10	0.11%
HAM-12	CASRQPLRGDYGYTF	NA	NA	NA	NA	NA	NA	102	10	0.11%
HAM-12	CASYNPRVSGANVLTF	15164	1	0.00%	669	3	0.01%	103	9	0.10%
HAM-12	CASSNPLAGLYEQYF	2523	2	0.01%	1851	1	0.00%	104	9	0.10%
HAM-12	CASSQGQGRQPQHF	11707	1	0.00%	NA	NA	NA	105	9	0.10%
HAM-12	CASSNPDRGYSPLHF	NA	NA	NA	2597	1	0.00%	106	9	0.10%
HAM-12	CSASQETQYF	NA	NA	NA	NA	NA	NA	107	8	0.09%
HAM-12	CASKQGLAGGSGELFF	NA	NA	NA	NA	NA	NA	108	8	0.09%

HAM-12	CASSQGLAGDPSGANVLTF	NA	NA	NA	1164	1	0.00%	109	8	0.09%
HAM-12	CASTTGDTQYF	3	734	1.92%	3	2541	5.66%	110	8	0.09%
HAM-12	CASIGGQLNTEAFF	1502	3	0.01%	NA	NA	NA	111	8	0.09%
HAM-12	CSARGVDRVGPGNEQFF	12617	1	0.00%	NA	NA	NA	112	8	0.09%
HAM-12	CASSQGLAGDYEQYF	NA	NA	NA	2537	1	0.00%	113	8	0.09%
HAM-12	CASSQGQGNTEAFF	13907	1	0.00%	490	4	0.01%	114	8	0.09%
HAM-12	CASSISPGQGAQNEQFF	NA	NA	NA	NA	NA	NA	115	8	0.09%
HAM-12	CASRPGLAGGPGNIQYF	8344	1	0.00%	NA	NA	NA	116	8	0.09%
HAM-12	CASSGGLAGDREQYF	NA	NA	NA	NA	NA	NA	117	7	0.08%
HAM-12	CASSPGRAGAKNIQYF	NA	NA	NA	NA	NA	NA	118	7	0.08%
HAM-12	CASSQPGTGETEAFF	NA	NA	NA	634	3	0.01%	119	7	0.08%
HAM-12	CASSESWGANAFF	NA	NA	NA	635	3	0.01%	120	7	0.08%
HAM-12	CASSNPLKGSYEQFF	NA	NA	NA	NA	NA	NA	121	7	0.08%
HAM-12	CASSDPHRSGHNEQFF	8053	1	0.00%	NA	NA	NA	122	7	0.08%
HAM-12	CASSQDPALMTGDTQYF	NA	NA	NA	NA	NA	NA	123	7	0.08%
HAM-12	CASSHPLAGGRNEQFF	NA	NA	NA	NA	NA	NA	124	7	0.08%
HAM-12	CASSYPLAGVNTQYF	1743	2	0.01%	NA	NA	NA	125	6	0.07%
HAM-12	CASSPEEDWGNTIYF	NA	NA	NA	NA	NA	NA	126	6	0.07%
HAM-12	CASSQGLAGGLEQFF	NA	NA	NA	671	3	0.01%	127	6	0.07%
HAM-12	CASSPGQGDSPLHF	NA	NA	NA	1076	2	0.00%	128	6	0.07%
HAM-12	CASSAQSSGNTIYF	NA	NA	NA	1834	1	0.00%	129	6	0.07%
HAM-12	CASSTGQGITYEQYF	NA	NA	NA	NA	NA	NA	130	6	0.07%
HAM-12	CASRPGLAGGHTQYF	NA	NA	NA	978	2	0.00%	131	6	0.07%
HAM-12	CATSGTGGSYEQYF	NA	NA	NA	NA	NA	NA	132	6	0.07%
HAM-12	CASSLPLAGGSYEQYF	NA	NA	NA	979	2	0.00%	133	6	0.07%
HAM-12	CASSKGLAGGLEQYF	NA	NA	NA	3373	1	0.00%	134	6	0.07%
HAM-12	CASSWEQGARAGELFF	NA	NA	NA	1900	1	0.00%	135	6	0.07%
HAM-12	CSAKGAGQFF	13402	1	0.00%	NA	NA	NA	136	6	0.07%
HAM-12	CASSHVDSTGELFF	5	686	1.80%	4	1914	4.26%	137	6	0.07%
HAM-12	CASSQGMGLVDTQYF	NA	NA	NA	3465	1	0.00%	138	6	0.07%
HAM-12	CASSYSRYPTSGSQETQYF	2500	2	0.01%	NA	NA	NA	139	6	0.07%
HAM-12	CASSHPLAVPPGRLYNEQFF	4818	1	0.00%	632	3	0.01%	140	5	0.05%
HAM-12	CASSYQLAGDYEQYF	9589	1	0.00%	NA	NA	NA	141	5	0.05%
HAM-12	CAISHDDPTQYF	5228	1	0.00%	NA	NA	NA	142	5	0.05%
HAM-12	CASSPYGQGQETQYF	NA	NA	NA	2638	1	0.00%	143	5	0.05%
HAM-12	CASSWGLAGGSGEQFF	NA	NA	NA	NA	NA	NA	144	5	0.05%
HAM-12	CASSYSRESGFSPLHF	NA	NA	NA	1183	1	0.00%	145	5	0.05%
HAM-12	CASSQAPRMMNTEAFF	NA	NA	NA	1213	1	0.00%	146	5	0.05%
HAM-12	CASRPGLAGGTGELFF	NA	NA	NA	NA	NA	NA	147	5	0.05%
HAM-12	CASSPPPKIMNTEAFF	NA	NA	NA	NA	NA	NA	148	5	0.05%
HAM-12	CASNAGLAGADTQYF	NA	NA	NA	NA	NA	NA	149	5	0.05%
HAM-12	CASSYPLGGNTEAFF	NA	NA	NA	NA	NA	NA	150	5	0.05%
HAM-12	CASIPVGTSGNNEQFF	NA	NA	NA	NA	NA	NA	151	4	0.04%
HAM-12	CASSQSPVTDFQETQYF	NA	NA	NA	NA	NA	NA	152	4	0.04%
HAM-12	CASSSGLAGGTGELFF	8440	1	0.00%	NA	NA	NA	153	4	0.04%
HAM-12	CASSPGQGNSPLHF	NA	NA	NA	2978	1	0.00%	154	4	0.04%
HAM-12	CASSENLQGWTEAFF	NA	NA	NA	1078	2	0.00%	155	4	0.04%

HAM-12	CASSPGLAGGIEQFF	NA	NA	NA	1856	1	0.00%	156	4	0.04%
HAM-12	CASSLGLAGAGGELFF	NA	NA	NA	NA	NA	NA	157	4	0.04%
HAM-12	CASSEPGQGMNYGYTF	NA	NA	NA	784	2	0.00%	158	4	0.04%
HAM-12	CASSYSRNPHKGFQETQYF	NA	NA	NA	NA	NA	NA	159	4	0.04%
HAM-12	CSASEGAAQYF	1763	2	0.01%	619	3	0.01%	160	4	0.04%
HAM-12	CASSNILAGVYEQYF	NA	NA	NA	NA	NA	NA	161	4	0.04%
HAM-12	CASNPGLAGTGELFF	NA	NA	NA	NA	NA	NA	162	4	0.04%
HAM-12	CASTRWGTVRGTEAFF	NA	NA	NA	1620	1	0.00%	163	4	0.04%
HAM-12	CASSSPLTGTNEQFF	NA	NA	NA	NA	NA	NA	164	4	0.04%
HAM-12	CAVSLAPGPLLTNQPQHF	NA	NA	NA	1035	2	0.00%	165	4	0.04%
HAM-12	CASSPGLAGGNEQFF	9872	1	0.00%	NA	NA	NA	166	4	0.04%
HAM-12	CASRLARTGGNTEAFF	NA	NA	NA	NA	NA	NA	167	4	0.04%
HAM-12	CASSHPMTAEQFF	NA	NA	NA	1106	2	0.00%	168	4	0.04%
HAM-12	CASNAGLRGGNEQFF	NA	NA	NA	NA	NA	NA	169	4	0.04%
HAM-12	CASSWLDNEQFF	NA	NA	NA	1996	1	0.00%	170	4	0.04%
HAM-12	CASGPGLAGASGELFF	NA	NA	NA	3538	1	0.00%	171	4	0.04%
HAM-12	CASSSGLAGGGTQYF	NA	NA	NA	2316	1	0.00%	172	4	0.04%
HAM-12	CASSQDPASFNTEAFF	NA	NA	NA	NA	NA	NA	173	4	0.04%
HAM-12	CASSPGQGVADTQYF	NA	NA	NA	2042	1	0.00%	174	3	0.03%
HAM-12	CASSQESLSWEQYF	NA	NA	NA	1436	1	0.00%	175	3	0.03%
HAM-12	CASRGGLAGVYPSTDTQYF	NA	NA	NA	NA	NA	NA	176	3	0.03%
HAM-12	CASSLGEVGTDHEKLFF	NA	NA	NA	1439	1	0.00%	177	3	0.03%
HAM-12	CASSQDPRAMNTEAFF	NA	NA	NA	NA	NA	NA	178	3	0.03%
HAM-12	CASSQDWRTSGAYNEQFF	NA	NA	NA	NA	NA	NA	179	3	0.03%
HAM-12	CASSGWRGRTDTQYF	NA	NA	NA	377	6	0.01%	180	3	0.03%
HAM-12	CASSSGLAGGPEQYF	NA	NA	NA	1187	1	0.00%	181	3	0.03%
HAM-12	CASSQGQGRNEQFF	NA	NA	NA	NA	NA	NA	182	3	0.03%
HAM-12	CASSPWESYNEQFF	NA	NA	NA	NA	NA	NA	183	3	0.03%
HAM-12	CASSGIGERSYEQYF	NA	NA	NA	NA	NA	NA	184	3	0.03%
HAM-12	CASSSGLAGAGGELFF	NA	NA	NA	NA	NA	NA	185	3	0.03%
HAM-12	CASSVLGGFGNTIYF	33	49	0.13%	23	196	0.44%	186	3	0.03%
HAM-12	CASSQPESMNTEAFF	NA	NA	NA	NA	NA	NA	187	3	0.03%
HAM-12	CASSPHGGGQPQHF	NA	NA	NA	3058	1	0.00%	188	3	0.03%
HAM-12	CASSSPGEGTYEQYF	NA	NA	NA	2758	1	0.00%	189	3	0.03%
HAM-12	CASRGGLGTANTEAFF	NA	NA	NA	NA	NA	NA	190	3	0.03%
HAM-12	CASSPGLAGGTGELFF	NA	NA	NA	NA	NA	NA	191	3	0.03%
HAM-12	CASSFWGQGTDTQYF	26	68	0.18%	20	218	0.49%	192	3	0.03%
HAM-12	CASSPWTLGSFFEQFF	NA	NA	NA	NA	NA	NA	193	3	0.03%
HAM-12	CASSSRDCYEQYF	9020	1	0.00%	NA	NA	NA	194	3	0.03%
HAM-12	CASSPVGGSDEQFF	NA	NA	NA	297	8	0.02%	195	3	0.03%
HAM-12	CASSQEQGLYTEAFF	NA	NA	NA	1639	1	0.00%	196	3	0.03%
HAM-12	CASSLDRVTGNTEAFF	NA	NA	NA	1644	1	0.00%	197	3	0.03%
HAM-12	CASSYPQAGNYEQYF	13861	1	0.00%	1951	1	0.00%	198	3	0.03%
HAM-12	CASSPAGQGTNEQFF	24	69	0.18%	18	226	0.50%	199	3	0.03%
HAM-12	CASIGPDGRGADTQYF	NA	NA	NA	3200	1	0.00%	200	3	0.03%
HAM-12	CASSSPLEGYYEQYF	NA	NA	NA	NA	NA	NA	201	3	0.03%
HAM-12	CATSDLSGPPYNEQFF	NA	NA	NA	NA	NA	NA	202	3	0.03%

HAM-12	CASSYSRDGTSGIEQYF	NA	NA	NA	964	2	0.00%	203	2	0.02%
HAM-12	CASISQNFYEQYF	NA	NA	NA	1765	1	0.00%	204	2	0.02%
HAM-12	CSANAETQYF	NA	NA	NA	NA	NA	NA	205	2	0.02%
HAM-12	CASSLGLAGGVEQFF	NA	NA	NA	822	2	0.00%	206	2	0.02%
HAM-12	CASTPGLAGGNEQYF	NA	NA	NA	2970	1	0.00%	207	2	0.02%
HAM-12	CASSEPLAGDYEQYF	4279	1	0.00%	NA	NA	NA	208	2	0.02%
HAM-12	CSVGATGNTIYF	11	133	0.35%	10	448	1.00%	209	2	0.02%
HAM-12	CASTTGLAGGSYEQYF	NA	NA	NA	NA	NA	NA	210	2	0.02%
HAM-12	CASSSPLRGENGYTF	NA	NA	NA	NA	NA	NA	211	2	0.02%
HAM-12	CASSPGLAAAKNIQYF	NA	NA	NA	467	4	0.01%	212	2	0.02%
HAM-12	CASSPGQGRDTQYF	NA	NA	NA	NA	NA	NA	213	2	0.02%
HAM-12	CASSAGLAGTADTQYF	NA	NA	NA	3035	1	0.00%	214	2	0.02%
HAM-12	CASSPRRLSSYNSPLHF	NA	NA	NA	NA	NA	NA	215	2	0.02%
HAM-12	CASSQDPGLGNYEQYF	13340	1	0.00%	NA	NA	NA	216	2	0.02%
HAM-12	CASSYPLAGEYEQYF	NA	NA	NA	2173	1	0.00%	217	2	0.02%
HAM-12	CASRQGLAGGTGEQFF	NA	NA	NA	1271	1	0.00%	218	2	0.02%
HAM-12	CASSPGTGAQPQHF	6604	1	0.00%	1279	1	0.00%	219	2	0.02%
HAM-12	CASSPFDRGGTEAFF	NA	NA	NA	NA	NA	NA	220	2	0.02%
HAM-12	CASSPGLAIAKNIQYF	NA	NA	NA	NA	NA	NA	221	2	0.02%
HAM-12	CASSSLLAGVYEQYF	NA	NA	NA	791	2	0.00%	222	2	0.02%
HAM-12	CASSPGLAGGPLQYF	NA	NA	NA	NA	NA	NA	223	2	0.02%
HAM-12	CSVEDPLGQGVNEQFF	3874	1	0.00%	NA	NA	NA	224	2	0.02%
HAM-12	CASRPGLASSYNEQFF	NA	NA	NA	NA	NA	NA	225	2	0.02%
HAM-12	CASSLWGSGTEAFF	NA	NA	NA	NA	NA	NA	226	2	0.02%
HAM-12	CASRPGLAGSSYEQYF	NA	NA	NA	NA	NA	NA	227	2	0.02%
HAM-12	CASSYSRGPGTSGRGFQETQYF	NA	NA	NA	NA	NA	NA	228	2	0.02%
HAM-12	CAISEGQGIGAGNTIYF	NA	NA	NA	916	2	0.00%	229	2	0.02%
HAM-12	CASTSWLAGVREQYF	NA	NA	NA	NA	NA	NA	230	2	0.02%
HAM-12	CASSPGLSPLAKNIQYF	NA	NA	NA	NA	NA	NA	231	2	0.02%
HAM-12	CSAGLGAPQYF	2689	2	0.01%	665	3	0.01%	232	2	0.02%
HAM-12	CASSQPLAGGSYEQYF	NA	NA	NA	3203	1	0.00%	233	2	0.02%
HAM-12	CASSLGLAGGIEQFF	NA	NA	NA	NA	NA	NA	234	2	0.02%
HAM-12	CASKQGLSSGANVLTF	NA	NA	NA	NA	NA	NA	235	2	0.02%
HAM-12	CASSTHRDRVGNEQFF	17	104	0.27%	12	371	0.83%	236	2	0.02%
HAM-13	CASSQGQGQETQYF	2333	2	0.01%	45	59	0.22%	1	69	21.84%
HAM-13	CASSHGPPRMNTEAFF	NA	NA	NA	NA	NA	NA	2	24	7.59%
HAM-13	CASRPGLAGARDEQFF	116	16	0.05%	57	47	0.18%	3	21	6.65%
HAM-13	CASRPGLAGAVDTQYF	189	11	0.04%	100	27	0.10%	4	20	6.33%
HAM-13	CASSDPLQGNYGYTF	NA	NA	NA	97	28	0.10%	5	18	5.70%
HAM-13	CASSPGLGSGLHEAFF	NA	NA	NA	156	16	0.06%	6	16	5.06%
HAM-13	CASSPGLAGGLSYNEQFF	NA	NA	NA	NA	NA	NA	7	15	4.75%
HAM-13	CASSWPGQGRNEQFF	NA	NA	NA	NA	NA	NA	8	14	4.43%
HAM-13	CASSSPGEGEETQYF	356	7	0.02%	146	17	0.06%	9	14	4.43%
HAM-13	CASRPGLAGATDTQYF	765	4	0.01%	172	13	0.05%	10	13	4.11%
HAM-13	CASSLTGMVETQYF	415	6	0.02%	128	20	0.07%	11	12	3.80%
HAM-13	CASRPGLAGGSGETQYF	169	12	0.04%	66	39	0.15%	12	12	3.80%
HAM-13	CASSGGLTGSSGANVLTF	2334	2	0.01%	401	4	0.01%	13	4	1.27%

HAM-13	CASSYPLQGIYEQYF	NA	NA	NA	341	5	0.02%	14	4	1.27%
HAM-13	CASSSPLSGNYGYTF	13604	1	0.00%	NA	NA	NA	15	4	1.27%
HAM-13	CASSNPLGGDYGYTF	11111	1	0.00%	2104	1	0.00%	16	4	1.27%
HAM-13	CASTFELGNEKLFF	NA	NA	NA	NA	NA	NA	17	3	0.95%
HAM-13	CASSQGQGNTEAFF	NA	NA	NA	3342	1	0.00%	18	3	0.95%
HAM-13	CASRPGLAGAHDEQYF	2155	2	0.01%	214	9	0.03%	19	3	0.95%
HAM-13	CASSQETRLMNTEAFF	NA	NA	NA	NA	NA	NA	20	3	0.95%
HAM-13	CASSWGSHNEQFF	NA	NA	NA	NA	NA	NA	21	2	0.63%
HAM-13	CASSSPLAGLYEQYF	2344	2	0.01%	790	2	0.01%	22	2	0.63%
HAM-13	CASSTYGGHSDTQYF	NA	NA	NA	NA	NA	NA	23	2	0.63%
HAM-13	CSATGQGYEQYF	NA	NA	NA	NA	NA	NA	24	2	0.63%
HAM-13	CASSPGLAGNADTQYF	NA	NA	NA	857	2	0.01%	25	2	0.63%
HAM-13	CASSPYLEPGSGELFF	NA	NA	NA	356	4	0.01%	26	2	0.63%
HAM-13	CASSEPLAGDYEQYF	NA	NA	NA	2636	1	0.00%	27	2	0.63%
HAM-13	CASSSSTYTEAFF	NA	NA	NA	NA	NA	NA	28	2	0.63%
HAM-13	CASSQGQGTLVGELFF	11479	1	0.00%	NA	NA	NA	29	2	0.63%
HAM-14	CSARAGQQFF	6	326	0.52%	4	1117	1.98%	1	289	40.25%
HAM-14	CASGQGLAGARGDTQYF	11	211	0.34%	6	717	1.27%	2	101	14.07%
HAM-14	CASSPGLAGGRGDTQYF	49	60	0.10%	24	202	0.36%	3	29	4.04%
HAM-14	CASSAGLAGARGDTQYF	58	52	0.08%	54	108	0.19%	4	26	3.62%
HAM-14	CASSKDFLAGGMSSYNEQFF	131	30	0.05%	74	83	0.15%	5	25	3.48%
HAM-14	CSARDLVDRSLNYGYTF	546	11	0.02%	129	53	0.09%	6	21	2.92%
HAM-14	CASSPGLAGSVRGPDTQYF	109	34	0.05%	51	111	0.20%	7	19	2.65%
HAM-14	CASSVGLAGGQGDTQYF	181	25	0.04%	80	78	0.14%	8	14	1.95%
HAM-14	CATSDSNGPLNTEAFF	218	22	0.04%	127	56	0.10%	9	14	1.95%
HAM-14	CASSPGLAGGTGELFF	2920	3	0.00%	530	10	0.02%	10	10	1.39%
HAM-14	CASSNPGTGDNEQFF	1589	5	0.01%	290	21	0.04%	11	10	1.39%
HAM-14	CSARDLGQGGSYEQYF	512	12	0.02%	445	13	0.02%	12	9	1.25%
HAM-14	CSASYGQPQHF	2117	4	0.01%	396	15	0.03%	13	6	0.84%
HAM-14	CASSHAQARGHTEAFF	1093	6	0.01%	278	22	0.04%	14	5	0.70%
HAM-14	CASSYSMEPTSGGYQETQYF	652	9	0.01%	199	32	0.06%	15	5	0.70%
HAM-14	CSARDLGADYGYTF	2940	3	0.00%	NA	NA	NA	16	5	0.70%
HAM-14	CASSWVLAGTYEQYF	508	12	0.02%	164	41	0.07%	17	5	0.70%
HAM-14	CASSEPHTGVTEAFF	NA	NA	NA	475	12	0.02%	18	5	0.70%
HAM-14	CASSTPGGQGAYEQYF	NA	NA	NA	539	10	0.02%	19	4	0.56%
HAM-14	CASSDPLKRGHNEQFF	465	13	0.02%	218	29	0.05%	20	4	0.56%
HAM-14	CASSSGLAGINEQFF	NA	NA	NA	NA	NA	NA	21	4	0.56%
HAM-14	CASSDPLQGDYGYTF	754	8	0.01%	427	13	0.02%	22	4	0.56%
HAM-14	CASSSGLAGVNEQFF	3661	2	0.00%	NA	NA	NA	23	4	0.56%
HAM-14	CASSVAGGGETQYF	868	8	0.01%	338	18	0.03%	24	4	0.56%
HAM-14	CASSYPLGGIYEQYF	NA	NA	NA	1301	3	0.01%	25	3	0.42%
HAM-14	CASSLGLAGGFGDTQYF	NA	NA	NA	1225	3	0.01%	26	3	0.42%
HAM-14	CSAREGTEAFF	NA	NA	NA	194	33	0.06%	27	3	0.42%
HAM-14	CSARAHEQFF	NA	NA	NA	14402	1	0.00%	28	3	0.42%
HAM-14	CASSLPGQGVNEQFF	NA	NA	NA	1847	2	0.00%	29	3	0.42%
HAM-14	CASSPILTGGTEAFF	6482	2	0.00%	424	14	0.02%	30	3	0.42%
HAM-14	CASSYPLGGIYGYTF	392	15	0.02%	185	36	0.06%	31	3	0.42%

HAM-14	CASSYPGQGDNEQFF	2295	3	0.00%	762	6	0.01%	32	3	0.42%
HAM-14	CASSPGLAGGPGDTQYF	730	9	0.01%	242	27	0.05%	33	3	0.42%
HAM-14	CASRPGLAGTDTQYF	22684	1	0.00%	NA	NA	NA	34	2	0.28%
HAM-14	CASSPDPLAGGYNEQFF	1878	4	0.01%	945	5	0.01%	35	2	0.28%
HAM-14	CSAKSAEQYF	NA	NA	NA	1889	2	0.00%	36	2	0.28%
HAM-14	CASSPGLAGGPSGANVLTF	NA	NA	NA	1640	2	0.00%	37	2	0.28%
HAM-14	CASSQVQGTEAFF	2011	4	0.01%	525	10	0.02%	38	2	0.28%
HAM-14	CASSPSGQGQETQYF	NA	NA	NA	NA	NA	NA	39	2	0.28%
HAM-14	CASSPRPEPLHF	1497	5	0.01%	411	14	0.02%	40	2	0.28%
HAM-14	CASSSPYSRGQTDTQYF	NA	NA	NA	NA	NA	NA	41	2	0.28%
HAM-14	CASSPGLAGGNEQFF	25763	1	0.00%	NA	NA	NA	42	2	0.28%
HAM-14	CASSPGLAGEADTQYF	NA	NA	NA	670	7	0.01%	43	2	0.28%
HAM-14	CASSPGLADSRGDTQYF	816	8	0.01%	549	9	0.02%	44	2	0.28%
HAM-14	CASKPGLAGGNTQYF	1276	6	0.01%	510	11	0.02%	45	2	0.28%
HAM-14	CSALSANTGELFF	NA	NA	NA	NA	NA	NA	46	2	0.28%
HAM-15	CSAKSAEQYF	2	230	0.79%	4	1157	1.66%	1	214	45.24%
HAM-15	CASSQAPRFMNTEAFF	9	110	0.38%	8	715	1.03%	2	70	14.80%
HAM-15	CASSQDPRLMNTEAFF	17	69	0.24%	10	517	0.74%	3	55	11.63%
HAM-15	CSAKAHEQFF	12	87	0.30%	11	489	0.70%	4	55	11.63%
HAM-15	CSAREVGRAAQETQYF	160	13	0.04%	88	50	0.07%	5	15	3.17%
HAM-15	CASSHRQGLNQPQHF	146	14	0.05%	82	59	0.08%	6	10	2.11%
HAM-15	CSARRGGTEAFF	186	11	0.04%	207	18	0.03%	7	5	1.06%
HAM-15	CASSVAGPGELFF	449	6	0.02%	150	26	0.04%	8	5	1.06%
HAM-15	CASSPPAGQGSLYEQYF	770	4	0.01%	98	42	0.06%	9	4	0.85%
HAM-15	CSGMGQPSYEQYF	725	4	0.01%	369	9	0.01%	10	4	0.85%
HAM-15	CASSQAPQEMNTEAFF	393	6	0.02%	91	47	0.07%	11	3	0.63%
HAM-15	CASSHPQAGVETQYF	10462	1	0.00%	162	24	0.03%	12	3	0.63%
HAM-15	CASSDPLTSGYNEQFF	NA	NA	NA	964	3	0.00%	13	3	0.63%
HAM-15	CASRPGLAGGSDTQYF	NA	NA	NA	336	10	0.01%	14	3	0.63%
HAM-15	CASSSPLAGAYEQYF	3443	1	0.00%	473	7	0.01%	15	2	0.42%
HAM-15	CSARDEGRGLHTEAFF	15967	1	0.00%	636	5	0.01%	16	2	0.42%
HAM-15	CASRPGLAGAEDTQYF	NA	NA	NA	248	14	0.02%	17	2	0.42%
HAM-15	CASSQEVELMNTEAFF	NA	NA	NA	289	12	0.02%	18	2	0.42%
HAM-15	CASRPGLAGPSIQYF	2145	2	0.01%	461	7	0.01%	19	2	0.42%

PBMCs, peripheral blood mononuclear cells; CDR3, complementary determining region3; NA, not available.

Table S3. The list of TCR-β sequences used for phylogenetic tree cluster analysis of expanded clones in Tax-specific CD8⁺ T cells

Seq ID	Read counts	Patient ID	CDR3 sequence	Cluster
seq323	8	HAM10_Tax	CASSDPGQGRNEQFF	1
seq281	10	HAM12_Tax	CASSESIGDNEQFF	1
seq99	22	HAM10_Tax	CASSFPGRDRGANEQFF	1
seq51	76	HAM10_Tax	CASSHPGTGVNEQFF	1
seq275	11	HAM12_Tax	CASSLGQGKNEQFF	1
seq309	10	HAM14_Tax	CASSNPGTGDNEQFF	1
seq56	70	HAM10_Tax	CASSPGLAALRSYNEQFF	1
seq155	10	HAM10_Tax	CASSPGLAGGNEQFF	1
seq60	63	HAM10_Tax	CASSPGLAGGRGNEQFF	1
seq79	30	HAM10_Tax	CASSPGLAGGRGNEQFF	1
seq37	127	HAM10_Tax	CASSPGLAGSNEQFF	1
seq204	81	HAM12_Tax	CASSSPGQGYNEQFF	1
seq225	38	HAM12_Tax	CASSSPLAGVYEQFF	1
seq135	12	HAM10_Tax	CASSSPLAVGYNEQFF	1
seq140	11	HAM10_Tax	CASSWGLASAGNEQFF	1
seq294	14	HAM13_Tax	CASSWPGQGRNEQFF	1
seq178	12	HAM11_Tax	CASSAGLAGNNEQFF	2
seq12	1475	HAM10_Tax	CASSFGLAGSFNNEQFF	2
seq195	99	HAM12_Tax	CASSIWGYNEQFF	2
seq303	25	HAM14_Tax	CASSKDFLAGGMSSYNEQFF	2
seq134	12	HAM10_Tax	CASSLDPLAGANNEQFF	2
seq191	201	HAM12_Tax	CASSLDPLAGGYNEQFF	2
seq94	24	HAM10_Tax	CASSLGLSGGYNEQFF	2
seq184	403	HAM12_Tax	CASSPLSYNEQFF	2
seq68	39	HAM10_Tax	CASSQDFRLAGAYNEQFF	2
seq257	15	HAM12_Tax	CASSQDPLAGGYNEQFF	2
seq215	60	HAM12_Tax	CASSQDPLASGANNEQFF	2
seq73	37	HAM10_Tax	CASSQDWMASGAYNEQFF	2
seq139	11	HAM10_Tax	CASSQGQGVNEQFF	2
seq5	17	HAM6_Tax	CASSQPHLAGGNEQFF	2
seq72	38	HAM10_Tax	CASSQPLAGYNEQFF	2
seq264	14	HAM12_Tax	CASSSDPLAGGYNEQFF	2
seq237	28	HAM12_Tax	CASSYGLAGGPKPYNEQFF	2
seq152	10	HAM10_Tax	CASSYSRSVGYNEQFF	2
seq198	90	HAM12_Tax	CATLPGLAGGFNEQFF	2
seq211	65	HAM12_Tax	CATREGLAGANVNEQFF	2
seq65	52	HAM10_Tax	CASIPGLAGGYNEQFF	3
seq260	15	HAM12_Tax	CASNKGLAGGPRGNEQFF	3
seq238	24	HAM12_Tax	CASNPGLAGGHEQFF	3
seq214	61	HAM12_Tax	CASRAGLAGGVEQFF	3

seq111	16	HAM10_Tax	CASRGGLAGGLGYNEQFF	3
seq319	8	HAM10_Tax	CASRPGLAGGVQNNEQFF	3
seq293	15	HAM13_Tax	CASSPGLAGGLSYNEQFF	3
seq245	19	HAM12_Tax	CASSYGLAGGIEQFF	3
seq273	11	HAM12_Tax	CASTHGLAGHTRSYNEQFF	3
seq86	27	HAM10_Tax	CASTPGLAGGPPEQFF	3
seq30	173	HAM10_Tax	CASTQGLAGVNEQFF	3
seq192	190	HAM12_Tax	CSAAQETQYF	4
seq231	33	HAM12_Tax	CSADGTSASEQFF	4
seq235	30	HAM12_Tax	CSAKAAEQFF	4
seq314	55	HAM15_Tax	CSAKAHEQFF	4
seq154	10	HAM10_Tax	CSAKAHEQYF	4
seq210	73	HAM12_Tax	CSAKANEQYF	4
seq230	34	HAM12_Tax	CSAKASEQFF	4
seq4	20	HAM6_Tax	CSAKASEQFF	4
seq311	214	HAM15_Tax	CSAKSAEQYF	4
seq247	18	HAM12_Tax	CSALYTGLSYEQYF	4
seq84	29	HAM10_Tax	CSANLNEQFF	4
seq242	21	HAM12_Tax	CSARADRDFF	4
seq299	289	HAM14_Tax	CSARAGQQFF	4
seq218	57	HAM12_Tax	CSARDLAGGAVIGNTQYF	4
seq310	9	HAM14_Tax	CSARDLGQGGSYEQYF	4
seq304	21	HAM14_Tax	CSARDLVDRSLNYGYTF	4
seq27	283	HAM10_Tax	CSARDRAGKETQYF	4
seq23	421	HAM10_Tax	CSARDVGLNYGYTF	4
seq315	15	HAM15_Tax	CSAREVGRAAQETQYF	4
seq106	17	HAM10_Tax	CSARFGAPLHF	4
seq332	8	HAM12_Tax	CSARGVDRVGPGNEQFF	4
seq229	35	HAM12_Tax	CSARSGEDTF	4
seq28	221	HAM10_Tax	CSARSGQDVF	4
seq327	8	HAM12_Tax	CSASQETQYF	4
seq197	98	HAM12_Tax	CSASSGDTQYF	4
seq196	99	HAM12_Tax	CSASTDTQYF	4
seq223	41	HAM12_Tax	CSASYGDTQYF	4
seq110	16	HAM10_Tax	CASSLRSPKGEAQYF	5
seq11	1929	HAM10_Tax	CASSLRSPKGETQYF	5
seq162	9	HAM10_Tax	CASSLRSPKGETQYF	5
seq85	28	HAM10_Tax	CASSLRSPKGETQYF	5
seq42	107	HAM10_Tax	CASSPGQGVMDAQYF	5
seq320	8	HAM10_Tax	CASSPGQGVMDTQYF	5
seq8	5331	HAM10_Tax	CASSPGQGVMDTQYF	5
seq117	14	HAM10_Tax	CASSPGQGVRDTQYF	5
seq75	33	HAM10_Tax	CASSQDFSIRNQETQYF	5
seq107	17	HAM10_Tax	CASSQDVASGGAPDTQYF	5

seq287	69	HAM13_Tax	CASSQGQGQETQYF	5
seq326	8	HAM10_Tax	CVSSPGQGVMDTQYF	5
seq302	26	HAM14_Tax	CASSAGLAGARGDTQYF	6
seq224	39	HAM12_Tax	CASSFLGGGQETQYF	6
seq269	12	HAM12_Tax	CASSGGLAGARETQYF	6
seq58	68	HAM10_Tax	CASSLAGGRASAGGPLGTQYF	6
seq297	12	HAM13_Tax	CASSLTGMVETQYF	6
seq138	11	HAM10_Tax	CASANPLGGGARDTQYF	7
seq136	12	HAM10_Tax	CASAPGLKGAADTQYF	7
seq300	101	HAM14_Tax	CASGQGLAGARGDTQYF	7
seq87	27	HAM10_Tax	CASTPGLKGAADAQYF	7
seq9	2452	HAM10_Tax	CASTPGLKGAADTQYF	7
seq150	10	HAM10_Tax	CASTPGVKGAADTQYF	7
seq330	8	HAM12_Tax	CASTTGDTQYF	7
seq147	11	HAM10_Tax	CASTTGLKGAADTQYF	7
seq190	219	HAM12_Tax	CASNPGLAGGSTDTQYF	8
seq20	480	HAM10_Tax	CASRPGLADGTDTQYF	8
seq141	11	HAM10_Tax	CASRPGLAGALDAQYF	8
seq22	428	HAM10_Tax	CASRPGLAGALDTQYF	8
seq199	89	HAM12_Tax	CASRPGLAGASDTQYF	8
seq296	13	HAM13_Tax	CASRPGLAGATDTQYF	8
seq290	20	HAM13_Tax	CASRPGLAGAVDTQYF	8
seq244	20	HAM12_Tax	CASRPGLAGGEASDTQYF	8
seq38	126	HAM10_Tax	CASTPGLAGGRSTDTQYF	8
seq70	38	HAM10_Tax	CASTSGLAGTDTQYF	8
seq161	9	HAM10_Tax	CASRDPLAGGHTQYF	9
seq156	10	HAM10_Tax	CASRGQGAVGYTF	9
seq165	9	HAM10_Tax	CASRGQGAVGYTF	9
seq32	153	HAM10_Tax	CASRMGLAGGPETQYF	9
seq243	20	HAM12_Tax	CASRPGLAGGPGGDTQYF	9
seq203	81	HAM12_Tax	CASRPGLAGGPRGDTQYF	9
seq298	12	HAM13_Tax	CASRPGLAGGSGETQYF	9
seq220	52	HAM12_Tax	CASRPGLAGGTLGADTQYF	9
seq100	21	HAM10_Tax	CASRTGLAGVGVREFF	9
seq301	29	HAM14_Tax	CASSPGLAGGRGDTQYF	10
seq322	8	HAM10_Tax	CASSPGLAGGRPPDTQYF	10
seq305	19	HAM14_Tax	CASSPGLAGSVRGPDTQYF	10
seq325	8	HAM10_Tax	CASSFGLAGGQGDAQYF	11
seq19	543	HAM10_Tax	CASSFGLAGGQGDTQYF	11
seq176	18	HAM11_Tax	CASSFGLAGGSTDTQYF	11
seq108	16	HAM10_Tax	CASSHGQGLTDAQYF	11
seq21	467	HAM10_Tax	CASSHGQGLTDTQYF	11
seq116	14	HAM10_Tax	CASSLGLAGGTDTQYF	11
seq36	127	HAM10_Tax	CASSPGQGITDTQYF	11

seq317	8	HAM10_Tax	CASSQGQGATDTQYF	11
seq53	74	HAM10_Tax	CASSQGQGITDTQYF	11
seq266	14	HAM12_Tax	CASSQGQGTTDTQYF	11
seq272	11	HAM12_Tax	CASSRWGQGSTDTQYF	11
seq17	853	HAM10_Tax	CASSSSPWPSTDTQYF	11
seq306	14	HAM14_Tax	CASSVGLAGGQGDTQYF	11
seq163	9	HAM10_Tax	CASSPGLAGAPGDAQYF	12
seq13	1272	HAM10_Tax	CASSPGLAGAPGDTQYF	12
seq7	13	HAM6_Tax	CASSYPGQGVWTQYF	12
seq170	77	HAM11_Tax	CASSHPPAGGANTQYF	13
seq148	11	HAM10_Tax	CASSPGLAGGETQYF	13
seq16	857	HAM10_Tax	CASSPGLAGGETQYF	13
seq279	10	HAM12_Tax	CASSPGLAGGETQYF	13
seq263	14	HAM12_Tax	CASSPGLAGGGETQYF	13
seq103	18	HAM10_Tax	CASSPGLAGGGWETQYF	13
seq71	38	HAM10_Tax	CASSPGLAGRQETQYF	13
seq194	103	HAM12_Tax	CASGPGLASAKNIQYF	14
seq182	566	HAM12_Tax	CASRPGLAEAKNIQYF	14
seq336	8	HAM12_Tax	CASRPGLAGGPGNIQYF	14
seq167	9	HAM10_Tax	CASSLDRLFKNIQYF	14
seq3	27	HAM6_Tax	CASSPGLSLAKNIQYF	14
seq276	11	HAM12_Tax	CASSPVTVTGSNIQYF	14
seq119	13	HAM10_Tax	CASSDPGQGSLYGYTF	15
seq212	63	HAM12_Tax	CASSDPLNTGAGGYTF	15
seq291	18	HAM13_Tax	CASSDPLQGNYGYTF	15
seq121	13	HAM10_Tax	CASSDPLQGVGYGYTF	15
seq118	14	HAM10_Tax	CASSESERGDYGYTF	15
seq62	60	HAM10_Tax	CASSFPFQGIYGYTF	15
seq146	11	HAM10_Tax	CASSLGLGQGAPGYTF	15
seq112	15	HAM10_Tax	CASSLVGSGGYTF	15
seq262	14	HAM12_Tax	CASSNPLEGANYGYTF	15
seq253	16	HAM12_Tax	CASSSGLAGDGSEQYF	15
seq173	24	HAM11_Tax	CASSSPFSRGAADTQYF	15
seq295	14	HAM13_Tax	CASSSPGEGEETQYF	15
seq280	10	HAM12_Tax	CASSSPGQGSYEQYF	15
seq241	22	HAM12_Tax	CASSSPGQGTPEQYF	15
seq44	99	HAM10_Tax	CASSSPGTGRETQYF	15
seq172	31	HAM11_Tax	CASSSPLAGDYEQYF	15
seq251	17	HAM12_Tax	CASSSPLDGIYGYTF	15
seq177	14	HAM11_Tax	CASSSPLTGLYGYTF	15
seq43	100	HAM10_Tax	CASSSPREGNYGYTF	15
seq41	109	HAM10_Tax	CASSSPRTGVYGYTF	15
seq160	10	HAM10_Tax	CASSYSIGGSDYGYTF	15
seq127	13	HAM10_Tax	CASRAGLAGVGEQYF	16

seq282	10	HAM12_Tax	CASRQPLRGDYGYTF	16
seq267	13	HAM12_Tax	CASSFPLAGDHEQYF	16
seq333	8	HAM12_Tax	CASSQGLAGDYEQYF	16
seq1	199	HAM6_Tax	CASSQPLAGDYEQYF	16
seq168	792	HAM11_Tax	CASSDPLTGHYEQYF	17
seq206	76	HAM12_Tax	CASSEEVGTPYEQYF	17
seq49	90	HAM10_Tax	CASSGIGDRSYEQYF	17
seq61	62	HAM10_Tax	CASSHGLAGDYEQYF	17
seq132	12	HAM10_Tax	CASSHGLAGSYEQYF	17
seq324	8	HAM10_Tax	CASSPDARTMNYEQYF	17
seq40	114	HAM10_Tax	CASSPLGRGDYEQYF	17
seq164	9	HAM10_Tax	CASSPREGSYEQYF	17
seq67	42	HAM10_Tax	CASSQDPLAGHYEQYF	17
seq34	135	HAM10_Tax	CASSQVLQRGASGEQYF	17
seq248	18	HAM12_Tax	CASSRAVGTGDYEQYF	17
seq268	12	HAM12_Tax	CASSRPLGGVYEQYF	17
seq90	26	HAM10_Tax	CASSSAGANPYEQYF	17
seq15	918	HAM10_Tax	CASSSGLAGGIYEQYF	17
seq137	12	HAM10_Tax	CATSPGLAGFYEQYF	17
seq126	13	HAM10_Tax	CATSTAGGSYEQYF	17
seq124	13	HAM10_Tax	CASIAPRQGIYEQYF	18
seq6	13	HAM6_Tax	CASSDPGQGNYEQYF	18
seq234	31	HAM12_Tax	CASSEPLTGAYEQYF	18
seq239	24	HAM12_Tax	CASSEPLTGSYEQYF	18
seq96	23	HAM10_Tax	CASSEPLTGYYEQYF	18
seq216	58	HAM12_Tax	CASSNPGRGSYEQYF	18
seq284	9	HAM12_Tax	CASSNPLAGLYEQYF	18
seq109	16	HAM10_Tax	CASSPPQGLAQEQYF	18
seq236	29	HAM12_Tax	CASSQGQGQDEQYF	18
seq159	10	HAM10_Tax	CASSQPFTSGSYEQYF	18
seq261	14	HAM12_Tax	CASSRPLAGRYEQYF	18
seq240	24	HAM12_Tax	CASSTPGTGVYEQYF	18
seq252	16	HAM12_Tax	CASSYPGQGAYEQYF	18
seq318	8	HAM10_Tax	CASSYPGTGSFEQYF	18
seq174	23	HAM11_Tax	CASSYPLAGKYEQYF	18
seq95	23	HAM10_Tax	CASSYPQAGTYEQYF	18
seq66	45	HAM10_Tax	CASSEPLAGAYEQYF	19
seq202	82	HAM12_Tax	CASSLPLAGVHEQYF	19
seq128	13	HAM10_Tax	CASSLPLAGVYEQYF	19
seq271	11	HAM12_Tax	CASSPGLAGAGHEQYF	19
seq331	8	HAM12_Tax	CASIGGQLNTEAFF	20
seq120	13	HAM10_Tax	CASRGGLTANTEAFF	20
seq187	309	HAM12_Tax	CASSLPVGEMNTEAFF	20
seq145	11	HAM10_Tax	CASSLSTGEVMNTEAFF	20

seq169	181	HAM11_Tax	CASTDIRFRGLGTEAFF	20
seq307	14	HAM14_Tax	CATSDSNGPLNTEAFF	20
seq76	32	HAM10_Tax	CAWSDPLVGSTEAFF	20
seq274	11	HAM12_Tax	CSASYGAEAFF	20
seq233	31	HAM12_Tax	CSGRLGTEAFF	20
seq205	78	HAM12_Tax	CASSAPPQDMNTEAFF	21
seq288	24	HAM13_Tax	CASSHGPPRMNTEAFF	21
seq39	119	HAM10_Tax	CASSLDPLESGMNTEAFF	21
seq219	56	HAM12_Tax	CASSLDPLFMNTEAFF	21
seq113	15	HAM10_Tax	CASSLPPTDMNTEAFF	21
seq25	293	HAM10_Tax	CASSPAATDMNTEAFF	21
seq255	15	HAM12_Tax	CASSPPAEDMNTEAFF	21
seq180	10	HAM11_Tax	CASSIHANRGDTEAFF	22
seq130	13	HAM10_Tax	CASSSYLGGNVEAFF	22
seq123	13	HAM10_Tax	CASSYTTGRVNTEAFF	22
seq47	92	HAM10_Tax	CASSFEQGSMNTEAFF	23
seq133	12	HAM10_Tax	CASSHPQGLNTEAFF	23
seq185	369	HAM12_Tax	CASSQEQGLNTEAFF	23
seq14	1176	HAM10_Tax	CASSQEQGVNTEAFF	23
seq142	11	HAM10_Tax	CASSQEQGVNTEAFF	23
seq334	8	HAM12_Tax	CASSQGQGNTEAFF	23
seq29	219	HAM10_Tax	CASSQPQGLHTEAFF	23
seq122	13	HAM10_Tax	CASSQPQGLNTEAFF	23
seq179	11	HAM11_Tax	CASSQPQGLNTEAFF	23
seq217	57	HAM12_Tax	CASSQPQGMNTEAFF	23
seq125	13	HAM10_Tax	CASSQQQGHTEAFF	23
seq115	15	HAM10_Tax	CASSPGQGTVAEAFF	24
seq89	26	HAM10_Tax	CASSPPQGRTEAFF	24
seq52	74	HAM10_Tax	CASSPPTAEMNTEAFF	24
seq312	70	HAM15_Tax	CASSQAPRFMNTEAFF	25
seq63	55	HAM10_Tax	CASSQDERVMVNTEAFF	25
seq104	18	HAM10_Tax	CASSQDHREMNTEAFF	25
seq93	24	HAM10_Tax	CASSQDPARMNTEAFF	25
seq2	198	HAM6_Tax	CASSQDPHLQGARTEAFF	25
seq246	18	HAM12_Tax	CASSQDPQVMDTEAFF	25
seq313	55	HAM15_Tax	CASSQDPRLMNTEAFF	25
seq10	2263	HAM10_Tax	CASSQRPEYMNTEAFF	25
seq98	22	HAM10_Tax	CASSQRPEYMNTEAFF	25
seq254	16	HAM12_Tax	CASSQVMGVNTEAFF	25
seq105	18	HAM10_Tax	CAISDLGKGSYNEQFF	26
seq149	11	HAM10_Tax	CARLAGEGCEQYF	26
seq33	145	HAM10_Tax	CASSATGTLIPEAFF	26
seq31	164	HAM10_Tax	CASSITSGRAPEQFF	26
seq69	38	HAM10_Tax	CASSLGLEQYF	26

seq186	329	HAM12_Tax	CASSQEARSLRVEQYF	26
seq270	11	HAM12_Tax	CASSQSWPYHNEQFF	26
seq189	230	HAM12_Tax	CASSQTGLLTHNEQFF	26
seq181	1465	HAM12_Tax	CATSRITSGEPYEQYF	26
seq114	15	HAM10_Tax	CAGQPTTNTGELFF	27
seq259	15	HAM12_Tax	CASRPGQGGELFF	27
seq228	35	HAM12_Tax	CASSEVLISGSAGELFF	27
seq321	8	HAM10_Tax	CASSLHGQGSKAGELFF	27
seq258	15	HAM12_Tax	CASSPPPGGANTGELFF	27
seq175	19	HAM11_Tax	CASSQVLGEGAAGELFF	27
seq166	9	HAM10_Tax	CASSQVLGQGAAGELFF	27
seq256	15	HAM12_Tax	CASSSAGGFGELFF	27
seq91	25	HAM10_Tax	CASSSPRTGTHSGANVLTF	27
seq277	10	HAM12_Tax	CASSVGQGTGELFF	27
seq283	9	HAM12_Tax	CASYNPRVSGANVLTF	27
seq201	86	HAM12_Tax	CASSDWYNGGANSPLHF	28
seq46	94	HAM10_Tax	CASSGTGYSPLHF	28
seq286	9	HAM12_Tax	CASSNPDRGYSPLHF	28
seq249	18	HAM12_Tax	CASSQDTLTGAYNSPLHF	28
seq102	19	HAM10_Tax	CASSQGQGKSPLHF	28
seq207	76	HAM12_Tax	CASSSPLQGGVSSPLHF	28
seq151	10	HAM10_Tax	CASTTLYGAGAGSPLHF	28
seq316	10	HAM15_Tax	CASSHRQGLNQPQHF	29
seq208	75	HAM12_Tax	CASSLWGGAQPQHF	29
seq26	284	HAM10_Tax	CASSPGPGQGHQPQHF	29
seq285	9	HAM12_Tax	CASSQGQGRQPQHF	29
seq57	68	HAM10_Tax	CASSYSSGQAHQPQHF	29
seq226	38	HAM12_Tax	CASSPGLAGGTSGANVLTF	30
seq329	8	HAM12_Tax	CASSQGLAGDPSGANVLTF	30
seq153	10	HAM10_Tax	CASSYGLEPSGANVLTF	30
seq193	115	HAM12_Tax	CASTQGLSGANVLTF	30
seq222	46	HAM12_Tax	CASSLGFGGRGNTIYF	31
seq64	53	HAM10_Tax	CASSPGLRGGLEQFF	31
seq171	56	HAM11_Tax	CATSDAGSGGNTIYF	31
seq213	62	HAM12_Tax	CATSGGLAGGHETQYF	31
seq328	8	HAM12_Tax	CASKQGLAGGSGELFF	32
seq143	11	HAM10_Tax	CASNPGLAGGTGELFF	32
seq50	89	HAM10_Tax	CASNPGLAGVAGELFF	32
seq77	32	HAM10_Tax	CASREGLAGGNRPGELFF	32
seq188	296	HAM12_Tax	CASRPGLAAPTGELFF	32
seq78	31	HAM10_Tax	CASRPGLAGGPGELFF	32
seq35	134	HAM10_Tax	CASRQGLDGGTGELFF	32
seq54	73	HAM10_Tax	CASRSGLAGDTGELFF	32
seq82	29	HAM10_Tax	CASSFGLAGVTGELFF	32

seq209	74	HAM12_Tax	CASSLAGGFGEQFF	32
seq45	98	HAM10_Tax	CASSLGLAGGPGELFF	32
seq129	13	HAM10_Tax	CASSPGLAGGLGTGELFF	32
seq308	10	HAM14_Tax	CASSPGLAGGTGELFF	32
seq18	633	HAM10_Tax	CASSPGLAGRTGELFF	32
seq97	23	HAM10_Tax	CASSPGLAGSSGELFF	32
seq265	14	HAM12_Tax	CASSPGLAGVGEQFF	32
seq158	10	HAM10_Tax	CASSPGLAIATGELFF	32
seq289	21	HAM13_Tax	CASRPGLAGARDEQFF	33
seq232	31	HAM12_Tax	CASSLGLAGPRGDEQFF	33
seq227	37	HAM12_Tax	CASSPGLAAAIVGQFF	33
seq221	49	HAM12_Tax	CASSYPGTGQYEQFF	33
seq80	30	HAM10_Tax	CASSHPLAGANEQFF	34
seq48	92	HAM10_Tax	CASSIGQGSDEQFF	34
seq335	8	HAM12_Tax	CASSISPGQGAQNEQFF	34
seq24	325	HAM10_Tax	CASSLGLAGGIVEQFF	34
seq144	11	HAM10_Tax	CASSLGLAGGVEQFF	34
seq88	26	HAM10_Tax	CASSLGTPEQFF	34
seq250	17	HAM12_Tax	CASSLGVPEQFF	34
seq157	10	HAM10_Tax	CASSPGLATFPEQFF	34
seq81	30	HAM10_Tax	CASSPLGGYEQFF	34
seq59	64	HAM10_Tax	CASSRGQGMYNEQFF	34
seq101	20	HAM10_Tax	CASSDGLAGVYEQFF	NA
seq74	34	HAM10_Tax	CASSDPGQGIFEQFF	NA
seq131	13	HAM10_Tax	CASSPDPLSGAHHEQFF	NA
seq292	16	HAM13_Tax	CASSPGLGSGLHEAFF	NA
seq55	71	HAM10_Tax	CASSQEQGRSEQFF	NA
seq278	10	HAM12_Tax	CASSQPLAGAHEQYF	NA
seq92	24	HAM10_Tax	CASSQVLTGGGNEKLFF	NA
seq83	29	HAM10_Tax	CASSSWGLNEKLFF	NA
seq200	87	HAM12_Tax	CASSVGQGRNEQYF	NA
seq183	447	HAM12_Tax	CASSYGLAGEPEQFF	NA

CDR3, complementary determining region 3

Table S4. List of antibodies for flow cytometric analysis

Antibody	Clone	Catalog number	Manufacturer
CD3	UCHT1	558117	BD Biosciences
CD3	HIT3a	300324	BioLegend
CD4	RPA-T4	557922	BD Biosciences
CD8	SK1	341051	BD Biosciences
CD8	HIT8a	300912	BioLegend
CD27	M-T271	560222	BD Biosciences
CD45	HI30	560777	BD Biosciences
CD45RA	HI100	555488	BD Biosciences
